# Revision of the sixgill sawsharks, genus *Pliotrema* (Chondrichthyes, Pristiophoriformes), with descriptions of two new species and a redescription of *P*. *warreni* Regan

**DOI:** 10.1371/journal.pone.0228791

**Published:** 2020-03-18

**Authors:** Simon Weigmann, Ofer Gon, Ruth H. Leeney, Ellen Barrowclift, Per Berggren, Narriman Jiddawi, Andrew J. Temple

**Affiliations:** 1 Elasmo-Lab, Elasmobranch Research Laboratory, Hamburg, Germany; 2 Center of Natural History, University of Hamburg, Hamburg, Germany; 3 South African Institute for Aquatic Biodiversity, Grahamstown, South Africa; 4 Natural History Museum, London, United Kingdom; 5 School of Natural and Environmental Sciences, Newcastle University, Newcastle-upon-Tyne, United Kingdom; 6 Institute of Fisheries Research, Ministry of Agriculture, Natural Resources, Livestock and Fisheries, Zanzibar, Tanzania; Laboratoire de Biologie du Développement de Villefranche-sur-Mer, FRANCE

## Abstract

Recent sampling efforts in Madagascar and Zanzibar, as well as examinations of six-gilled sawsharks in several museum collections provided evidence for a complex of species within *Pliotrema warreni* Regan. The present manuscript contains a redescription of *P*. *warreni* involving the syntypes and additional material, as well as formal descriptions of two new species of *Pliotrema* Regan. All specimens of both new species were found in the western Indian Ocean. Individuals of the first new species, hereafter referred to as *P*. *kajae* sp. nov., were identified originating from Madagascar and the Mascarene Ridge. Specimens of the second new species, hereafter referred to as *P*. *annae* sp. nov., were only found off Zanzibar. *Pliotrema kajae* sp. nov. appears to inhabit upper insular slopes and submarine ridges at depths of 214–320 m, *P*. *annae* sp. nov. so far is only known from shallow waters (20–35 m). Both new species differ from *P*. *warreni* in a number of characteristics including the known distribution range and fresh coloration. Taxonomical differences include barbels that are situated approximately half way from rostral tip to mouth, with prebarbel length equidistant from barbel origin to symphysis of the upper jaw in *P*. *kajae* sp. nov. and *P*. *annae* sp. nov. (vs. about two thirds way from rostral tip to mouth, with prebarbel length about twice the distance from barbel origin to symphysis of upper jaw in *P*. *warreni*) and rostra that are clearly and slightly constricted between barbel origin and nostrils, respectively (vs. rostrum not constricted). *Pliotrema kajae* sp. nov. differs from *P*. *annae* sp. nov. in a longer snout, more numerous large lateral rostral teeth and upper jaw tooth rows, jaw teeth with (vs. without) sharp basal folds, and coloration, particularly pale to light brown (vs. medium to dark brown) dorsal coloration with (vs. without) two indistinct yellowish stripes. A revised diagnosis of *Pliotrema* and a key to the species are provided.

## Introduction

Pristiophoriform sharks (sawsharks) possess a flat, greatly elongated, and saw-like snout, which bears long ventral barbels and closely-set rows of lateral and ventral teeth [1–3]. Further, the anterior-most basiventral cartilages are laterally expanded and have curved, dorsally reflected margins [4,5]. Based on genetic analyses, previous studies have shown that the Pristiophoriformes form a clade with the Squaliformes and Squatiniformes but with the exclusion of the Hexanchiformes [6–8]. However, Naylor et al. [8] highlighted that the interrelationships between the Echinorhinidae, Pristiophoriformes, and Squatiniformes remain unclear due to weak support in the respective datasets. Interestingly, the current proposed interrelationships appear to differ significantly between published studies [9–11], highlighting the need to clarify understanding of the interrelationships of these groups. Regardless of the lack of detailed empirical data, there is morphological support for the interrelationships reported by Naylor et al. [6–8] for the Pristiophoriformes and Squatiniformes by, e.g. [12] based mainly on skeletal and myological features, as well as for the Pristiophoriformes and Squaliformes by, e.g. [3] based on characteristics of the claspers. However, Weigmann et al. [3] pointed out that, despite sharing many characteristics, the claspers of pristiophoriforms and squatiniforms differ greatly. These authors proposed that the similarity in clasper characters between pristiophoriforms and squaliforms could be regarded as a plesiomorphic character, whereas the differences in clasper morphology between pristiophoriforms and squatiniforms could be considered as autapomorphies, and the similarities in body shape and morphology of the first basiventral cartilages of the latter two orders as evidences of interrelationship.

Members of the monotypic genus *Pliotrema* Regan clearly differ from species of *Pristiophorus* Müller and Henle in their possession of six (vs. five) pairs of gill slits and serrated (vs. smooth-edged) large lateral rostral teeth [3]. Furthermore, Springer and Bullis [4] reported that the bases of the jaw teeth of species from the genus *Pliotrema* have 3 to 6 short but distinct ridges, somewhat stronger on the upper jaw teeth, whereas these ridges are indistinct or absent in *Pristiophorus* species. The only recorded species of *Pliotrema*, *P*. *warreni* Regan, has a known distribution from the southwestern Indian Ocean and southeastern Atlantic, and has been recorded at depths between 26 and 500 m [13,14]. It has been reported to reach a total length (TL) of up to 170 cm, making *P*. *warreni* the largest known species of sawshark [14,15]. *Pristiophorus* species are small to medium-sized, demersal sharks known from continental and insular shelves and upper slopes of tropical and temperate latitudes of all three major oceans, but with the center of distribution in the western Pacific Ocean and only one recently described, poorly known dwarf species in the western Indian Ocean [2,14]. Members of this genus attain maximum sizes of 62–153 cm TL and occur in depths between 0 and 1240 m [14].

While collecting data on elasmobranchs caught in small-scale fisheries in Madagascar, RHL collected two rostra of *Pliotrema*. Subsequently, EB, AJT and PB collected a specimen of *Pliotrema* from off Zanzibar during a 12-month fisheries landings observer program across the coastal regions of Kenya, Zanzibar, and northern Madagascar [16]. The rostra and the specimen from off Zanzibar turned out to represent two undescribed species of *Pliotrema*. During a search of museum collection, SW further identified a number of complete specimens of the same undescribed species represented by the two rostra, NJ and AJT managed to collect an additional specimen of the other new species from off Zanzibar, which was transported to the UK by PB. The availability of all these specimens enabled formal descriptions of both new species based on morphological, morphometric, and meristic data. The formal descriptions and a redescription of *P*. *warreni* based on the syntypes and additional specimens are presented herein; a revised generic diagnosis and a key to the species of *Pliotrema* are also given.

## Materials and methods

Institutional acronyms follow Sabaj [17] except for DMM = Deutsches Meeresmuseum in Stralsund (Germany), ERB = Elasmobranch Research Belgium, Bonheiden (Belgium), RHL = Ruth H. Leeney personal collection, SW = Simon Weigmann personal collection. Specimens were fixed with formalin and stored in 70% ethanol except for dried rostra. Morphometric measurements were taken between perpendicular lines, where relevant, by vernier caliper to one tenth of a millimeter and follow Compagno [18], Yearsley et al. [19] and Weigmann et al. [3]. In measurements involving the ventral origin of the caudal fin, the origin was set anteriorly including the low anterior fin ridge following Kaschner et al. [20], Weigmann et al. [21], Weigmann and Kaschner [22], and Weigmann et al. [23]. Vertebral counts and terminology follow Springer and Garrick [24]. Vertebrae were counted from radiographs, tooth rows from radiographs and directly from specimens. Teeth, rostral teeth, and dermal denticles were examined using a scanning electron microscope (SEM). The map with the catch locations of the examined specimens of all three species of *Pliotrema* was generated based on the Global Relief Model ETOPO1 by NOAA (the National Oceanic and Atmospheric Administration) [25]. Country borders, lakes, and rivers were visualized by means of the shapefiles supplied by ESRI for the ArcExplorer-Java Edition for Education 2.3.2 (AEJEE). None of the specimens analyzed in this study were collected specifically for the purposes of this study. All specimens examined are preserved in scientific collections. No special permissions were required to obtain specimens as the areas fished were not protected.

### Comparative material examined

#### *Pristiophorus cirratus* (Latham) (2 specimens)

**LACM 42620–20**: adult male, 870 mm TL, east of Sydney (33°46’S 151°49’E), collected by C.C Swift and pty on 09 September 1981 with 27 m headrope otter trawl, 421–507 m depth. **ZMH 8503**: juvenile male, 506 mm TL, RV ‘Southern Surveyor’, Station SS 5/94/95 (Bass Strait: 38°42.4’S 148°16.8’E), collected on 26 August 1994 with bottom trawl, 86–87 m depth.

#### *Pristiophorus japonicus* Günther (4 specimens)

Syntype (**BMNH 1862.11.1.37**): juvenile male, 734 mm TL, off Japan (photographs only). Syntype (**BMNH 1867.2.20.1**), female, ~1000 mm TL, off Japan (photographs only). Syntype (**BMNH 1867.2.20.2**), female, ~1200 mm TL, off Japan (photographs only). Syntype (**BMNH 1953.8.10.6**): juvenile female, ~700 mm TL, off Japan (photographs only).

#### *Pristiophorus lanae* Ebert and Wilms (1 specimen)

**ZSM 45812**: presumably adult female, 900 mm TL, off Culasi, Antique province, Panay Island, Philippines, collected on 10 March 2016 by Alexandra Bagarinar and Wilfredo L. Campos.

#### *Pristiophorus nancyae* Ebert and Cailliet (23 specimens)

Holotype (**SAM 34013/MB-F034013**): adult male, 616 mm TL, RV ‘Algoa’, Mozambique Scad Survey, Station C00840 014 037 3074 (Mozambique Channel: 22°07’S 35°45’E), collected on 19 June 1994 with bottom trawl, 500 m depth (photographs only). Paratype (**SAM 33477/MB-F033477**): subadult male, 440 mm TL, same data as holotype (photographs only). Paratype (**SAM 33502/MB-F033502**): subadult female, 573 mm TL, RV ‘Algoa’, Mozambique Scad Survey, Station C00848 014 045 3179 (off South Mozambique: 25°21’S 34°30’E), collected on 21 June 1994 with bottom trawl, 286 m depth (photographs only). Five paratypes (**SAM 33511/MB-F033511**): two juvenile males (314 and 358 mm TL), one juvenile female (391 mm TL), two adult females (522 and 550 mm TL), RV ‘Algoa’, Mozambique Scad Survey, Station C00841 014 038 3118 (Mozambique Channel: 23°32’S 35°51’E), collected on 20 June 1994 with bottom trawl, 490 m depth (photographs only). **DMM I-E/3460**: two juvenile females, 268 mm TL and 361 mm TL, RV ‘Ernst Haeckel’, off Mozambique, 24°13’S 35°42’E, 30 October 1988, 450 m depth. **DMM I-E/4506**: adult female, 640 mm TL, RV ‘Ernst Haeckel’ Cruise 51, Haul 89/80, off Mozambique, 23°56’S 35°48’E, 15 June 1980 (photographs only). **DMM I-E/4817**: juvenile male, 351 mm TL, RV ‘Ernst Haeckel’ Cruise 51, Haul 567/80, off Mozambique, 23°56’S 35°48’E, 21 September 1980. **DMM I-E/4872**: adult male, 555 mm TL, off Mozambique, February to March 1983. **DMM I-E/4902**: adult female, 700 mm TL, off Mozambique, February to March 1983. **ZMH 25963**: adult male, 581 mm TL fresh, 578 mm TL 70% ethanol preserved, RV ‘Vityaz’ Cruise 17, Station 2560 (off Socotra Islands: 12°16’6”N 53°08’2”E–12°14’7”N 53°06’2”E), collected on 27 October 1988 with 29 m shrimp trawl, trawl no. 2, on the bottom for 45 min, 375–380 m depth. **ZMH 25964**: adult male, 547 mm TL fresh, 540 mm TL 70% ethanol preserved, same data as ZMH 25963. **ZMH 25965**: adult male, 563 mm TL fresh, 558 mm TL 70% ethanol preserved, same data as ZMH 25963. **ZMH 25966**: juvenile male, 334 mm TL fresh, 328 mm TL 70% ethanol preserved, same data as ZMH 25963. **ZMH 25967**: juvenile female, 327 mm TL fresh, 324 mm TL 70% ethanol preserved, same data as ZMH 25963. **ZMH 25968**: adult female, 574 mm TL fresh, 568 mm TL 70% ethanol preserved, RV ‘Vityaz’ Cruise 17, Station 2631 (off South Mozambique: 25°30’4”S 35°08’2”E–25°34’2”S 35°01’5”E), collected on 23 November 1988 with 29 m shrimp trawl, trawl no. 28, on the bottom for 78 min, 500–570 m depth. **ZMH 25969**: juvenile female, 283 mm TL fresh, 275 mm TL 70% ethanol preserved, RV ‘Vityaz’ Cruise 17, Station 2830 (off Socotra Islands: 12°14’8”N 53°06’2”E–12°17’8”N 53°08’9”E), collected on 16 January 1989 with 29 m shrimp trawl, trawl no. 101, on the bottom for 80 min, 395–420 m depth. **ZMH 25970**: juvenile female, 374 mm TL fresh, 367 mm TL 70% ethanol preserved, same data as ZMH 25969. **ZMMU P 14847**: adult female, 621 mm TL, RV ‘Prof. Mesyatsev’ Cruise 5, Station 10 (off Kenya: 02°59’5”S 40°30’E), collected by A.D. Druzhinin on 22 December 1975, 287–300 m depth.

#### *Pristiophorus nudipinnis* Günther (1 specimen)

**ZMH 8504**: juvenile male, 474 mm TL, RV ‘Southern Surveyor’, Station SS 5/94/30 (Bass Strait: 39°00.1’S 146°35.8’E), collected on 24 August 1994 with bottom trawl, 43–44 m depth.

#### *Pristiophorus schroederi* Springer and Bullis (4 specimens)

Holotype (**USNM 185946**): juvenile female, 383 mm TL, RV ‘Combat’, Station 449 (east of Dog Rocks, Cay Sal Bank: 24°05’N 79°46’W), collected on 24 June 1957 with beam trawl, 640 m depth (radiographs only). Two paratypes (**USNM 185947**): juvenile male, 645 mm TL and female, 805 mm TL, RV ‘Silver Bay’, Station 445 (north of Little Bahama Bank: 28°03’N 78°46’W), collected on 09 June 1958, 914–951 m depth (radiographs only). **USNM 202479**: subadult male, 766 mm TL, RV ‘Silver Bay’, Station 2458 (Santaren Channel off Cuba: 23°40’N 79°18’W), collected by S. Springer on 05 November 1960 with bottom trawl, 530 m depth.

### Nomenclatural Acts

The electronic edition of this article conforms to the requirements of the amended International Code of Zoological Nomenclature, and hence the new names contained herein are available under that Code from the electronic edition of this article. This published work and the nomenclatural acts it contains have been registered in ZooBank, the online registration system for the ICZN. The ZooBank LSIDs (Life Science Identifiers) can be resolved and the associated information viewed through any standard web browser by appending the LSID to the prefix “http://zoobank.org/”. The LSID for this publication is: urn:lsid:zoobank.org:pub:6281D5F4-DC2B-4E6D-B2F9-97734E545220. The electronic edition of this work was published in a journal with an ISSN, and has been archived and is available from the following digital repositories: PubMed Central, LOCKSS.

## Results

### Systematic account

**Order Pristiophoriformes Berg**.

**Family Pristiophoridae Bleeker**.

**Genus *Pliotrema* Regan**.

**Type species**. *Pliotrema warreni* Regan by original designation.

#### Revised generic diagnosis of *Pliotrema* Regan

Pristiophoriform sharks are characterized by a flat, greatly elongated, and saw-like snout with long ventral barbels and closely-set rows of lateral teeth and ventral spines, as well as anterior-most basiventral cartilages that are laterally expanded and have curved, dorsally reflected margins. The teeth on the lateral edges of the snout are alternately large and small in juveniles but the number of small teeth in the interspaces between large teeth increases ontogenetically. The individual rostral teeth are fixed to the dermis and not embedded in sockets. The ventral spines are more or less pronounced and decrease in size and partially get lost with age, in some species they are colored black. In embryos, the lateral rostral teeth and ventral rostral spines are folded back beneath the skin. Oral teeth small, with conical cusp on broad base, arranged in several series.

The genus *Pliotrema* is characterized by the possession of six gill slits and serrated large lateral rostral teeth. Like *Pristiophorus*, *Pliotrema* lacks nictitating lower eyelids, has large spiracles situated behind the eyes, two dorsal fins without spines–the first in front of pelvic fins, no anal fin, no precaudal pit, and a caudal fin with subterminal notch and strongly reduced lower lobe. In *Pliotrema*, barbel origin to anterior nostrils 1.4–2.3 times anterior nostrils to symphysis upper jaw; prenarial length 1.3–1.7 times prebarbel length; preoral length 1.5–2.7 times interdorsal space; pectoral-fin anterior margin 1.2–1.6 times dorsal–caudal space; mouth width 2.7–6.6 times spiracle length. First dorsal fin originates about opposite pectoral-fin free rear tips. Lateral trunk dermal denticles tricuspidate, rather flat and imbricated. Color uniform pale to dark brown dorsally, sometimes with one or two weak to rather pronounced yellowish longitudinal stripes, partially with whitish fin margins (particularly caudal and pectoral fins); ventrally whitish, partially with sparse darker mottling; dorsal rostrum surface with two distinct longitudinal dark stripes, lateral rostral teeth edged dark. Monospondylous centra 52–57; precaudal diplospondylous centra 46–56; total vertebral centra 151–164.

#### *Pliotrema kajae* Weigmann, Gon, Leeney & Temple sp. nov.

urn:lsid:zoobank.org:act:D4EF80CA-3448-4015-A96C-279C5A0A7970.

Proposed English vernacular name: Kaja’s sixgill sawshark.

Proposed German vernacular name: Kajas Sechskiemer-Sägehai.

Local name: vae vae.

Figs 1–14; Tables 1–2.

*Poliotrema warreni* (misspelling for *Pliotrema*): Séret [26]: 1.

*Pliotrema warreni*: Compagno et al. [27]: 73 (in part), based on Séret [26].

Assuming that this new species does not occur off the southern African continent (South Africa, Mozambique), no records of *Pliotrema kajae* sp. nov. (as *Pliotrema warreni*) before Séret [26] have been found. All subsequent records of *Pliotrema* from off Madagascar are apparently based on Séret [26]. The holotype is deposited in the Muséum national d’Histoire naturelle, Paris (MNHN), 10 paratypes are deposited in the South African Institute for Aquatic Biodiversity (SAIAB), and one paratype in each of the Natural History Museum, London (BMNH), Ruth H. Leeney personal collection (RHL), and Simon Weigmann personal collection (SW), National Museum of Natural History, Smithsonian Institution, Washington D.C. (USNM), and Zoological Museum Hamburg (ZMH).

Holotype **MNHN 1987–1266**, juvenile female, 560 mm TL, off Tulear (Madagascar), 23°19’58.8” S 43°31’1.2” E, 320 m depth, Dec 1985.

Paratypes (15) **SAIAB 84039**, gravid female, 1170 mm TL fresh, 1143 mm TL 70% ethanol preserved, RV ‘Dr. Fridtjof Nansen’, Survey 2008407, Station 7, Mascarene Ridge, 16°27.62’S 60°16.84’E, 214–219 m depth, bottom trawl # 22, duration 27.3 minutes, 14 Oct 2008 (taken together with 1 further specimen, which was not retained); **SAIAB 84096**, adult male, 970 mm TL fresh, 940 mm TL 70% ethanol preserved, RV ‘Dr. Fridtjof Nansen’, Survey 2008407, Station 11, Mascarene Ridge, 15°41.11’S 61°4.54’E, 302–305 m depth, bottom trawl # 22, duration 34.3 minutes, 18 Oct 2008 (taken together with 1 further specimen, which was not retained); **SAIAB 189447**, 1 gravid female, 3 of 6 mid- to late-term embryos (1 male: 246 mm TL; 2 females, 320 mm TL, 324 mm TL; three embryos of 243 mm TL, 318 mm TL, and 329 mm TL were donated to the ZMH, BMNH and USNM collections, respectively), and 4 early embryos (71+ mm TL with tail broken off, 95 mm TL, 103 mm TL, 110 mm TL), RV ‘Dr. Fridtjof Nansen’, Survey 2009408, Station 24, off western Madagascar, 21°58.79’S 43°8.38’E, 235–239 m depth, trawl, duration 30.1 minutes, 07 Sep 2009; **BMNH 2019.1.28.1 (ex SAIAB 189447)**, male late-term embryo, 318 mm TL, data the same as SAIAB 189447; **RHL-Mad-01** (dried rostrum), presumably adult female, estimated TL 1100 mm (TL estimated based on the prebarbel length [160 mm] in comparison to the ratios prebarbel length to TL in other type specimens), taken off southwestern Madagascar by local fishermen; **SW 01–2016** (dried rostrum), presumably adult female, estimated TL 1300 mm (prebarbel length 191.1 mm), taken off southwestern Madagascar by local fishermen, **USNM 443683 (ex SAIAB 189447)**, male late-term embryo, 329 mm TL, data the same as SAIAB 189447; **ZMH 26360 (ex SAIAB 189447)**, female mid-term embryo, 243 mm TL, data the same as SAIAB 189447.

#### Diagnosis

A large six-gilled sawshark with the following characters: barbel origin to anterior nostrils 1.4–2.3 times anterior nostrils to symphysis upper jaw; prenarial length 1.5–1.7 times prebarbel length; preoral length 2.0–2.7 times interdorsal space; pectoral-fin anterior margin 1.2–1.6 times dorsal–caudal space; mouth width 2.8–6.6 times spiracle length. First dorsal fin originates about opposite pectoral-fin free rear tips. Lateral trunk dermal denticles tricuspidate, rather flat and imbricated. Color pale to light brown dorsally with two thin yellowish longitudinal stripes; uniform white ventrally; fins with rather indistinct white posterior fin margins; dorsal rostrum surface with two distinct longitudinal dark stripes, lateral rostral teeth dark-edged. Monospondylous centra 52–57; precaudal diplospondylous centra 48–56; total vertebral centra 151–164. This new species is distinguished from its two congeners, *Pliotrema warreni* and the second new species, by a combination of characteristics, including most notably, a rostrum that is clearly constricted between barbel origin and nostrils. Furthermore, *P*. *kajae* has sharp folds in both upper and lower jaw teeth, as well as a posteriorly notched, teardrop-shaped dorsal fenestra of the precerebral fossa. *Pliotrema kajae* is further distinguished from *P*. *warreni* by barbels that are situated about half way from rostral tip to mouth, with prebarbel length about equidistant from barbel origin to symphysis of upper jaw (vs. barbels about two thirds way from rostral tip to mouth, with prebarbel length about twice distance from barbel origin to symphysis of upper jaw) and the presence of two indistinct, yellowish longitudinal stripes on the dorsal surface (vs. one pronounced yellowish longitudinal stripe). *Pliotrema kajae* also clearly differs from the second new species in a generally longer snout, more upper and lower jaw tooth rows, higher total large lateral rostral tooth and ventral rostral spine counts, and a pale to light brown dorsal coloration with two indistinct yellowish stripes, uniform white ventral coloration, and posterior fin margins with narrow white edges (vs. uniform medium to dark brown dorsally without longitudinal stripes, white ventrally but with few indistinct dark blotches on belly, posterior fin margins conspicuously white-edged).

#### Description of the holotype

Values of the paratypes are presented in parentheses, more complex differences between holotype and paratypes are described separately. Where relevant, ratios are based on horizontal measurements unless otherwise stated. Morphometric measurements and meristics are given in Table 1.

**Table 1:** *Pliotrema kajae* sp. nov., morphometrics and meristics. Individual values for the juvenile holotype (MNHN 1987–1266), gravid female paratype SAIAB 84039, and adult male paratype SAIAB 84096, as well as ranges for six embryonic paratypes and means for the holotype and eight paratypes. Proportional values are expressed as percentages of total length (TL) 70% ethanol preserved except for minimum, maximum, and mean of TL in mm.

	*Pliotrema kajae* sp. nov., juvenile female holotype, MNHN 1987–1266		*Pliotrema kajae* sp. nov., gravid female paratype, SAIAB 84039		*Pliotrema kajae* sp. nov., adult male paratype, SAIAB 84096		Minimum (n = 6)	Maximum (n = 6)	Mean (n = 9)
	mm	% TL	mm	% TL	mm	% TL	% TL	% TL	% TL
TL, total length	560.0	100.0	1143.0	100.0	940.0	100.0	243.0	329.0	491.4
PRC, precaudal length, dorsally	453.0	80.9	955.0	83.6	780.0	83.0	78.3	81.3	80.8
PRVC, precaudal length, ventrally	458.0	81.8	975.0	85.3	800.0	85.1	80.2	82.2	82.3
PD2, pre-D2-length	380.0	67.9	806.0	70.5	655.0	69.7	64.9	69.7	68.1
PD1, pre-D1-length	273.0	48.8	555.0	48.6	450.0	47.9	47.2	50.6	48.9
HDL, head length (to end of last gill slit), horizontally	216.4	38.6	438.0	38.3	380.0	40.4	38.4	40.0	39.2
HDL, head length (to end of last gill slit), point to point	219.1	39.1	440.0	38.5	375.0	39.9	38.9	40.7	39.6
PG1, prebranchial length, horizontally	198.9	35.5	391.0	34.2	335.0	35.6	34.7	36.6	35.4
PG1, prebranchial length, point to point	201.4	36.0	394.0	34.5	340.0	36.2	35.0	37.2	35.8
PSP, prespiracular length, horizontally	172.8	30.9	332.0	29.0	300.0	31.9	30.7	31.6	31.0
PSP, prespiracular length, point to point	174.8	31.2	335.0	29.3	303.0	32.2	30.6	32.2	31.3
POB, preorbital length, horizontally	149.9	26.8	295.0	25.8	257.0	27.3	25.7	27.1	26.3
POB, preorbital length, point to point	152.7	27.3	300.0	26.2	260.0	27.7	26.0	27.6	26.7
PP1, prepectoral length, horizontally	213.7	38.2	438.0	38.3	367.0	39.0	38.5	40.5	39.3
PP2, prepelvic length, horizontally	319.0	57.0	676.0	59.1	545.0	58.0	56.0	59.8	57.8
SVL, snout–anterior vent length	334.0	59.6	707.0	61.9	575.0	61.2	59.0	61.6	60.3
IDS, interdorsal space	73.2	13.1	164.0	14.3	138.0	14.7	11.6	13.8	13.0
DCS, dorsal (D2)–caudal space	43.4	7.7	91.0	8.0	75.5	8.0	6.7	9.1	7.9
PPS, pectoral–pelvic space	90.0	16.1	204.0	17.8	157.2	16.7	13.5	18.5	16.5
PCA, pelvic–caudal space	118.2	21.1	256.0	22.4	220.0	23.4	17.3	21.4	20.5
VCL, anterior vent–caudal tip length	226.0	40.4	445.0	38.9	375.0	39.9	38.7	40.2	39.6
PRN, prenarial length, horizontally	146.3	26.1	280.0	24.5	246.0	26.2	24.1	26.3	25.2
POR, preoral length	171.2	30.6	327.0	28.6	288.0	30.6	29.8	31.3	30.2
EYL, eye length	18.6	3.3	32.2	2.8	27.9	3.0	4.1	5.2	4.0
EYH, eye height	8.0	1.4	14.9	1.3	11.3	1.2	1.8	2.9	1.9
ING, intergill length 1st to last slit	14.7	2.6	45.0	3.9	29.9	3.2	4.0	4.9	4.0
GS1, 1st gill slit height (unspread)	7.7	1.4	15.8	1.4	10.5	1.1	1.2	2.1	1.5
GS2, 2nd gill slit height	8.0	1.4	16.1	1.4	12.6	1.3	1.3	2.1	1.6
GS3, 3rd gill slit height	8.1	1.4	16.4	1.4	12.6	1.3	1.2	2.5	1.6
GS4, 4th gill slit height	7.9	1.4	16.1	1.4	11.2	1.2	1.2	2.3	1.5
GS5, 5th gill slit height	7.4	1.3	15.5	1.4	11.6	1.2	0.9	2.2	1.4
GS6, 6th gill slit height	7.7	1.4	14.7	1.3	10.1	1.1	0.8	2.0	1.3
P1A, pectoral anterior margin length	63.8	11.4	140.0	12.2	104.0	11.1	10.3	11.3	11.1
P1B, pectoral base length	19.1	3.4	42.9	3.7	32.3	3.4	3.0	3.7	3.4
P1I, pectoral inner margin length	42.9	7.7	71.2	6.2	60.1	6.4	6.8	7.6	7.0
P1P, pectoral posterior margin length	51.9	9.3	113.3	9.9	79.0	8.4	6.7	8.5	8.2
P1H, pectoral height, base end to tip	63.6	11.4	122.8	10.7	97.6	10.4	8.9	10.1	10.0
P1L, P length anterior base to posterior tip	58.3	10.4	116.7	10.2	88.9	9.5	8.8	10.3	9.6
CDM, dorsal caudal margin length	105.3	18.8	195.0	17.1	163.8	17.4	18.5	19.9	18.6
CST, subterminal caudal margin length	15.2	2.7	22.3	2.0	16.6	1.8	2.4	3.7	2.8
CSW, subterminal caudal width	14.9	2.7	26.6	2.3	23.1	2.5	2.1	3.1	2.6
CTR, terminal caudal margin length	20.5	3.7	46.4	4.1	38.3	4.1	2.4	5.0	3.8
CTL, terminal caudal lobe length	29.0	5.2	55.5	4.9	48.7	5.2	4.1	6.0	5.0
D1L, D1 total length	51.7	9.2	108.7	9.5	82.5	8.8	7.2	9.8	8.8
D1A, D1 anterior margin length	59.9	10.7	105.2	9.2	84.4	9.0	10.0	11.4	10.3
D1B, D1 base length	35.0	6.3	78.7	6.9	61.8	6.6	4.9	6.5	6.1
D1H, D1 vertical height	37.4	6.7	72.8	6.4	60.2	6.4	5.9	7.1	6.4
D1I, D1 inner margin length	17.7	3.2	28.5	2.5	22.5	2.4	2.6	3.4	2.9
D1P, D1 posterior margin length	29.0	5.2	72.8	6.4	54.3	5.8	5.3	6.8	6.0
D2L, D2 total length	47.1	8.4	97.9	8.6	73.1	7.8	7.6	8.6	8.2
D2A, D2 anterior margin length	54.7	9.8	89.5	7.8	79.7	8.5	8.7	10.0	9.1
D2B, D2 base length	31.6	5.6	65.2	5.7	52.8	5.6	5.1	6.3	5.6
D2H, D2 vertical height	34.6	6.2	66.5	5.8	58.6	6.2	4.5	5.8	5.5
D2I, D2 inner margin length	15.8	2.8	26.0	2.3	20.4	2.2	2.2	2.9	2.5
D2P, D2 posterior margin length	32.3	5.8	66.2	5.8	53.0	5.6	3.7	5.1	5.0
P2L, pelvic total length	43.6	7.8	85.5	7.5	79.4	8.4	6.8	7.9	7.5
P2A, pelvic anterior margin length	36.6	6.5	65.4	5.7	49.4	5.3	5.7	6.7	6.0
P2B, pelvic base length	24.4	4.4	42.8	3.7	45.0	4.8	3.3	4.7	4.1
P2H, pelvic height = max. width (excl. clasper)	26.6	4.8	46.5	4.1	40.0	4.3	4.1	4.8	4.5
P2I, pelvic inner margin length	19.0	3.4	42.5	3.7	37.0	3.9	3.1	4.3	3.7
P2P, pelvic posterior margin length	26.7	4.8	62.0	5.4	51.0	5.4	3.0	4.9	4.6
HDH, head height at P origin	29.7	5.3	75.2	6.6	71.2	7.6	4.5	7.4	6.1
TRH, trunk height at P base end	33.7	6.0	83.4	7.3	73.5	7.8	4.6	7.7	6.4
ABH, abdomen height at D1 base end	29.4	5.2	82.9	7.3	72.4	7.7	3.7	8.2	6.4
TAH, tail height at pelvic base end	26.8	4.8	57.9	5.1	45.3	4.8	3.3	4.7	4.2
CPH, caudal peduncle height at dorsal caudal-fin origin	12.3	2.2	22.5	2.0	19.3	2.1	2.1	2.5	2.2
DPI, D1 midpoint–pectoral base end	45.2	8.1	128.2	11.2	114.8	12.2	10.3	14.7	11.6
DPO, D1 midpoint–pelvic origin	32.8	5.9	82.6	7.2	92.8	9.9	5.4	8.9	7.3
PDI, pelvic midpoint–D1 base end	28.4	5.1	78.4	6.9	83.6	8.9	5.8	6.9	6.5
PDO, pelvic midpoint–D2 origin	44.3	7.9	88.9	7.8	90.5	9.6	6.0	9.7	8.1
MOL, mouth length (arc radius)	4.9	0.9	9.9	0.9	8.7	0.9	1.0	1.3	1.1
MOW, mouth width	25.0	4.5	49.9	4.4	43.2	4.6	4.7	5.4	4.9
ULA, upper labial furrow length	0.0	0.0	0.0	0.0	0.0	0.0	0.0	0.0	0.0
LLA, lower labial furrow length	2.4	0.4	6.0	0.5	3.9	0.4	0.4	0.5	0.4
NOW, nostril width	4.4	0.8	9.4	0.8	7.6	0.8	0.7	1.0	0.9
INW, internarial width	19.8	3.5	35.9	3.1	31.3	3.3	3.8	4.3	3.9
ANF, anterior nasal flap length	5.1	0.9	8.0	0.7	6.9	0.7	1.2	1.6	1.2
INOI, interorbital space, integumental	24.9	4.5	47.3	4.1	39.2	4.2	5.2	5.9	5.0
INOS, interorbital space, skeletal	16.1	2.9	31.1	2.7	27.9	3.0	3.3	4.2	3.3
SPL, spiracle length	7.6	1.4	18.0	1.6	13.4	1.4	0.8	1.4	1.2
ESL, eye–spiracle space	3.6	0.6	4.7	0.4	5.8	0.6	0.3	0.9	0.6
HDW, head width at middle gill slits	37.3	6.7	83.3	7.3	70.2	7.5	6.8	8.7	7.9
TRW, trunk width at P base ends	37.0	6.6	86.4	7.6	68.7	7.3	5.7	7.1	6.6
ABW, abdomen width at D1 base end	34.8	6.2	82.3	7.2	70.4	7.5	4.0	7.2	6.0
TAW, tail width at pelvic base ends	23.6	4.2	63.3	5.5	45.6	4.9	3.5	4.3	4.1
CPW, C peduncle width at dorsal caudal-fin origin	12.5	2.2	26.7	2.3	21.3	2.3	1.6	2.7	2.1
CLO, clasper outer margin length	-	-	-	-	26.2	2.8	-	-	2.8
CLI, clasper inner margin length	-	-	-	-	65.4	7.0	-	-	7.0
CLB, clasper base width	-	-	-	-	14.2	1.5	-	-	1.5
BAL, Barbel length	55.4	9.9	75.7	6.6	65.9	7.0	15.3	18.1	13.8
PBL, Prebarbel length, horizontally	87.5	15.6	169.6	14.8	148.4	15.8	15.1	16.2	15.6
BSJ, Barbel origin to symphysis upper jaw	83.7	15.0	157.4	13.8	135.9	14.5	13.7	15.0	14.4
BAN, Barbel origin to anterior nostrils	58.0	10.4	109.7	9.6	98.9	10.5	8.5	9.7	9.5
ANJ, Anterior nostrils to symphysis upper jaw	25.0	4.5	53.7	4.7	47.0	5.0	4.9	6.0	5.1
INS, Interspiracular space	26.2	4.7	47.4	4.1	42.5	4.5	4.9	5.7	5.0
RWN, Rostral width at anterior nostrils	33.8	6.0	60.6	5.3	54.6	5.8	6.9	7.5	6.7
RWB, Rostral width at origin of barbels	23.7	4.2	40.7	3.6	35.1	3.7	4.5	5.1	4.5
RTAL, Rostral tooth length (anterior of nostrils): Length of longest tooth immediately anterior to barbel	6.1	1.1	10.7	0.9	lost	lost	2.1	2.3	1.9
RTAW, Rostral tooth width (anterior of nostrils): Width of exposed base of above tooth	1.2	0.2	2.8	0.2	lost	lost	0.3	0.4	0.3
RTIS, 1° rostral tooth interspace: First complete interspace anterior to barbels	7.2	1.3	11.9	1.0	lost	lost	0.9	1.9	1.2
RTIL, 2° rostral tooth length: Longest complete tooth within above primary interspace	2.8	0.5	5.5	0.5	lost	lost	-	-	0.5
RTPL, Rostral tooth length (posterior of nostrils): Longest rostral tooth in this region	3.3	0.6	3.0	0.3	2.0	0.2	-	-	0.4
spiracle folds left/right	12/13		12/14		13/13		12/12	15/15	13.0/13.6
total large lateral rostral teeth l./r.	21/21		23/23		22/22		28/28	31/31	26.1/26.3
large lateral rostral teeth anterior to barbels l./r.	13/13		13/13		13/13		12/13	14/14	13.0/13.3
large lateral rostral teeth posterior to barbels l./r.	8/8		10/10		9/9		15/15	17/17	13.1/13.0
ventral rostral spines anterior to nostrils l./r.	23/23		23/23		23/23		19/19	24/22	21.4/21.6
ventral rostral spines anterior to barbel origin l./r.	13/12		13/13		13/13		11/12	14/13	12.7/12.7
tooth rows, upper jaw	43		38		41		-	-	40.7
tooth rows, lower jaw	35		37		37		-	-	36.3
Vtr, monospondylous trunk vertebrae centra	57		53		56		52	55	54.4
dipl. VprC, diplospondylous precaudal vertebrae centra	49		48		54		51	56	52.3
VprC, total precaudal vertebrae centra	106		101		110		105	110	106.6
VtermC, caudal vertebrae centra	54		50		54		47	53	51.5
total vertebrae centra	160		151		164		152	162	158.1

**Fig 1:**
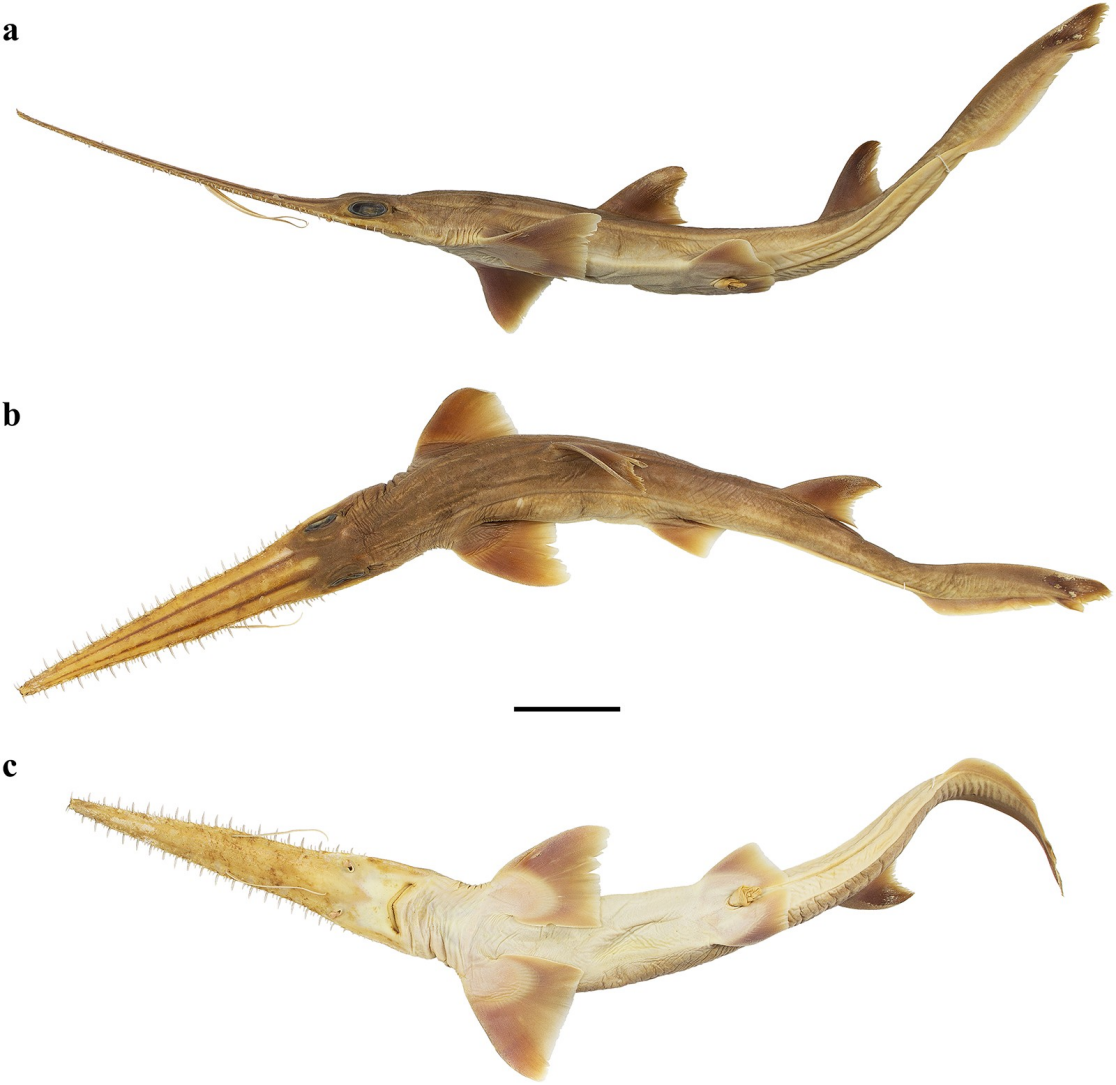
*Pliotrema kajae* sp. nov., holotype, MNHN 1987–1266, juvenile female, 560 mm TL, preserved. **a** lateral, **b** dorsal, and **c** ventral views. Scale bar: 5 cm.

**Fig 2:**
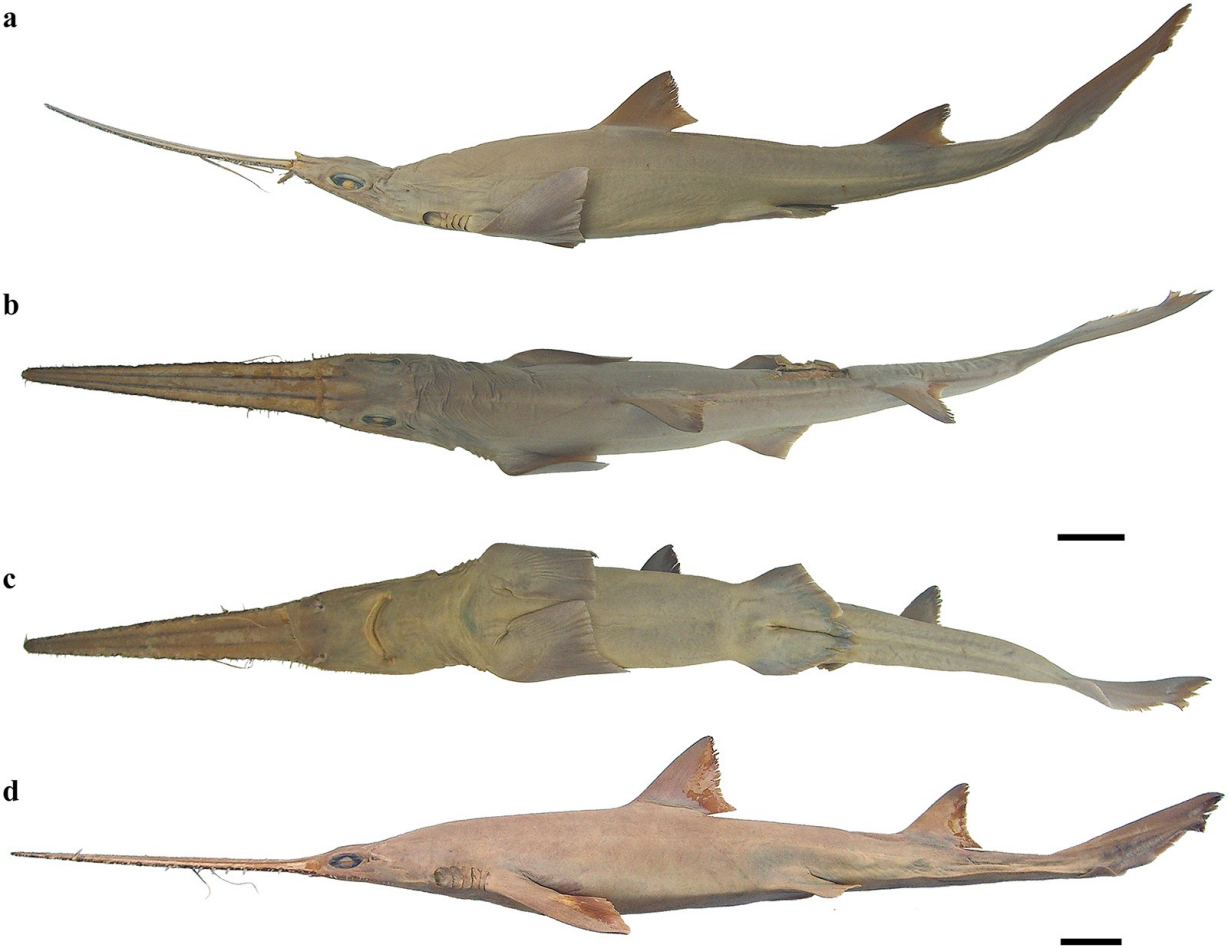
*Pliotrema kajae* sp. nov. paratypes, preserved. **a**,**b**,**c** paratype SAIAB 84096, adult male, 940 mm TL, in **a** lateral, **b** dorsal, and **c** ventral views; **d** paratype SAIAB 84039, gravid female, 1143 mm TL, in lateral view. Scale bars: 5 cm.

External morphology. Body firm and slender, depressed forward of gills, abdomen subcircular in cross-section, tail subtriangular in cross-section, deepest at abdomen; not tapering gradually and evenly beyond pectoral fins; snout flattened, greatly extended, saw-like; abdomen elongate, horizontal head length 0.6 (0.6–0.7) times snout–anterior vent length, pectoral–pelvic space 16.1 (13.5–18.5)% TL; pelvic–caudal space 2.7 (2.5–3.0) times pelvic-fin length; tail flattened ventrally, elongate, snout–anterior vent length 1.5 (1.5–1.6) times anterior vent–caudal tip length; caudal peduncle short, dorsal–caudal space 7.7 (6.7–9.1)% TL, caudal peduncle height 3.5 (2.8–4.0) times in dorsal–caudal space and width 1.0 (0.8–1.6) times in height; ventrolateral keels well developed, extending from slightly behind level of free rear tip of pelvic fins (from about level to slightly behind level) to beyond origin of ventral lobe of caudal fin, converging strongly near their posterior extremity; no precaudal pit; no median predorsal, postdorsal or preventral caudal grooves (Figs 1 and 2).

**Fig 3:**
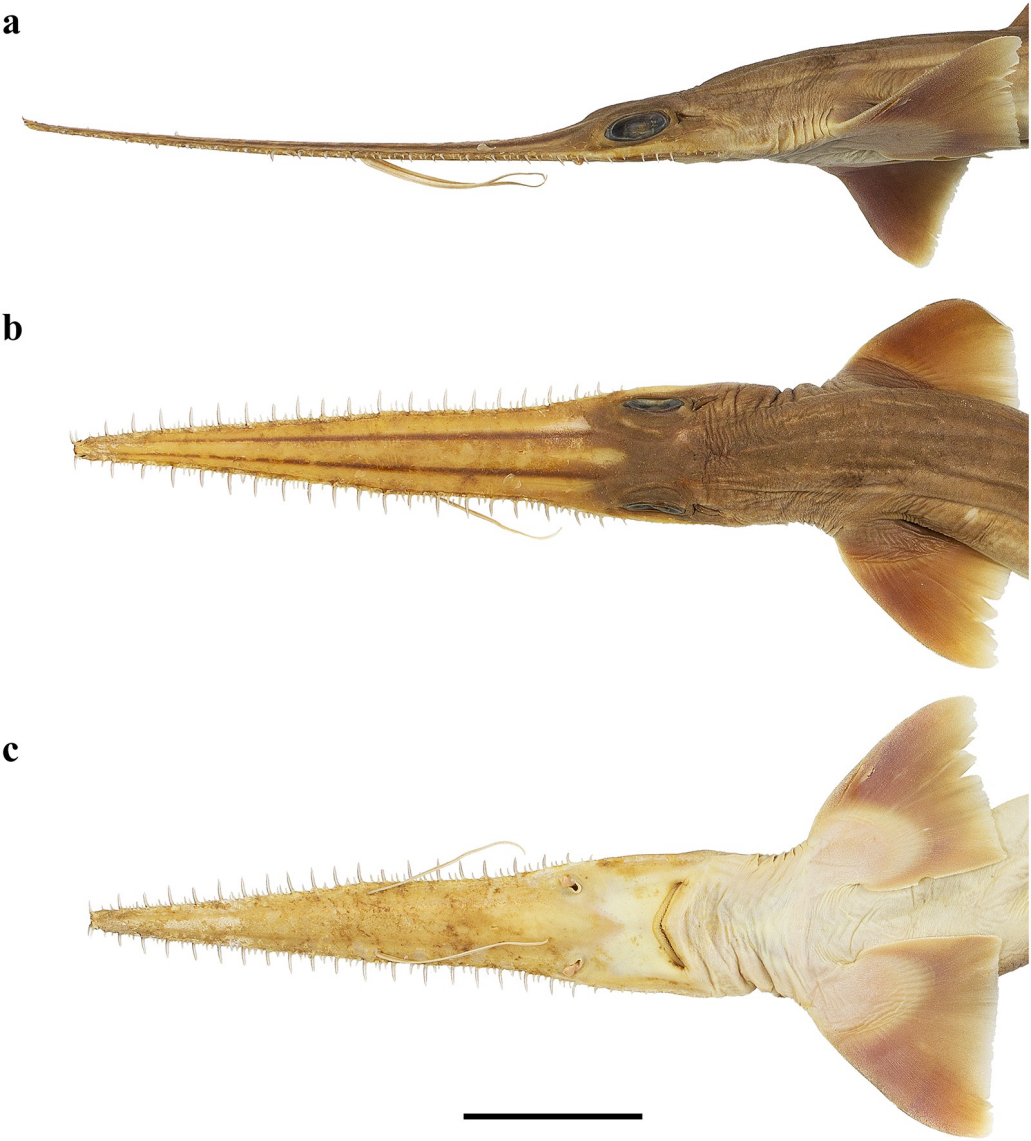
*Pliotrema kajae* sp. nov., holotype, MNHN 1987–1266, juvenile female, 560 mm TL, preserved. Head in **a** lateral, **b** dorsal, and **c** ventral views. Scale bar: 5 cm.

Head narrow, subtriangular and deepest at sixth gill slit, strongly depressed above eyes, head width 6.7 (6.8–8.7)% TL, 1.3 (0.9–1.9) times head height. Snout forming a very elongate, blade-like rostrum. Rostrum triangular in dorsal view; constricted between barbel origin and nostrils, sides of rostrum nearly straight from tip to barbel origin but concave in posterior part from barbel origin to origin of orbit; tip narrowly rounded; rostrum extending laterally below eyes as a well-defined suborbital ridge along ventrolateral edge of head, terminating somewhat behind level of posterior edge of spiracle (Fig 3).

A slender, filamentous, dorsoventrally flattened barbel originating on the ventrolateral margin about half way from rostral tip to mouth on each side, with prebarbel length 1.0 (1.0–1.1) times distance from barbel origin to symphysis of upper jaw, 51.1 (49.4–52.9)% of preoral length and 15.6 (14.8–16.2)% TL. Barbel length 1.6 (0.8–2.3) times in prebarbel length and 1.5 (0.8–2.1) times in length from barbel origin to symphysis of upper jaw. Preorbital length, horizontally 6.0 (4.8–6.0) times mouth width, 19.7 (16.4–31.5) times spiracle length, 2.9 (2.7–3.6) times first dorsal-fin length, 4.4 (3.4–4.9) times rostral width at anterior nostrils; extremely narrow in lateral view; preoral length 30.6 (28.6–31.3)% TL, 4.6 (3.4–4.4) times head width, 5.1 (4.0–5.4) times rostral width at anterior nostrils, 7.2 (6.0–8.2) times rostral width at origin of barbels, 2.0 (1.9–2.0) times prebarbel length, 1.2 (1.2–1.3) times prenarial length, and 2.3 (2.0–2.7) times interdorsal space (Fig 3).

**Fig 4:**
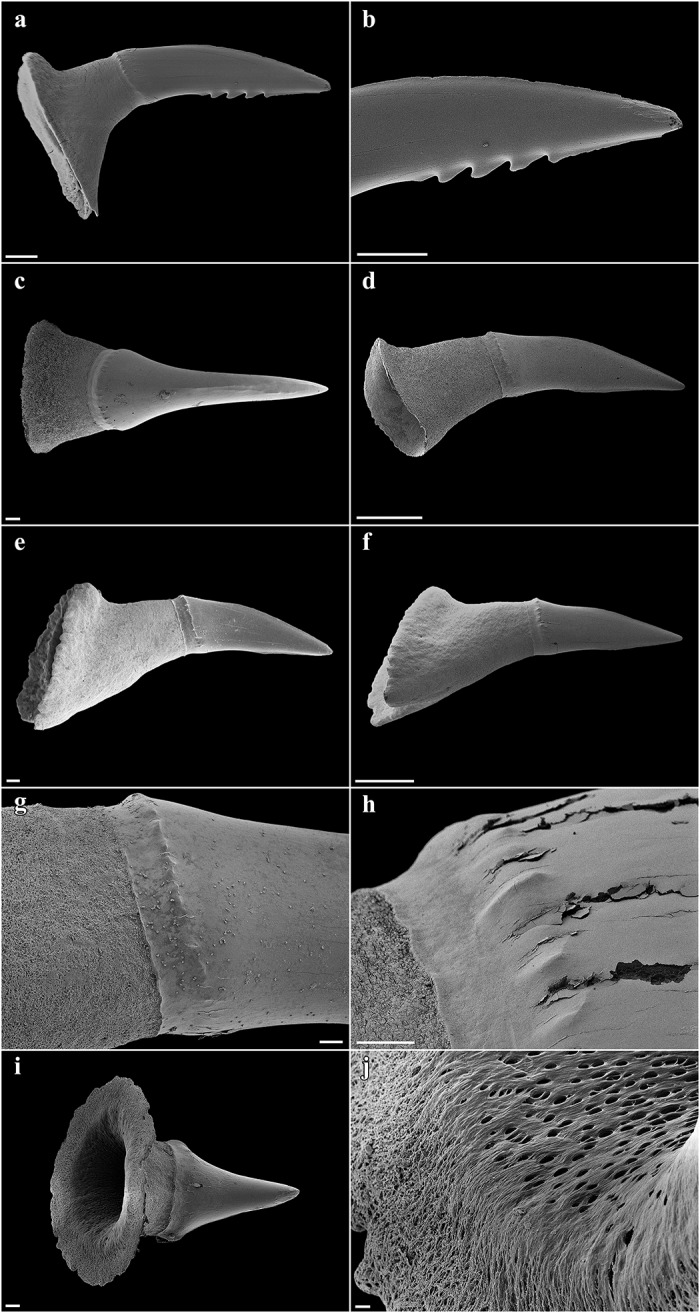
*Pliotrema kajae* sp. nov., paratype, RHL-Mad-01, presumably adult female, estimated TL 1100 mm, SEM images of rostral teeth. **a**,**b** large lateral rostral tooth in **a** total and **b** close-up views; **c**–**h** small interstitial lateral rostral teeth in **c–f** total and **g–h** close-up views; **i**,**j** ventral rostral spine in **i** total and **j** close-up views. Scale bars: **a**,**b**,**d**,**f** 1 mm, **c**,**e** 200 μm, **g**–**i** 100 μm, **j** 20 μm.

Large lateral rostral teeth of prenarial portion of rostrum variable in length, curved, rather stout, serrated, longest near barbel origin and near apex of rostrum posterior to anteriormost two teeth; longest tooth immediately anterior to barbels only slightly shorter than spiracle length, length 1.1 (0.9–2.3)% TL and 0.8 (0.9–2.6) times first complete interspace anterior to barbels, width 0.2 (0.2–0.4)% TL; anteriormost two large rostral teeth on each side of the rostrum very close to snout tip, without interstitial tooth between or anterior to them; large teeth shortest near nostrils, longest rostral tooth posterior to nostrils 0.6 (0.2–0.3)% TL; large teeth absent behind nostrils but interstitial-like teeth present, short to very short and closely set, partially directed almost ventrally, particularly near mouth. Interspaces between large rostral teeth rather regularly sized, about as long as adjacent teeth, with 2–4 (1–4) smaller, variable interstitial teeth. Rostral tooth counts mostly symmetrical between left and right hand sides; left side with 21 (22–31) large teeth, right side with 21 (22–31); anterior to barbels left side with 13 (12–14) large rostral teeth, right side with 13 (13–14), posterior to barbels left side with 8 (9–17) large rostral teeth, right side with 8 (9–17); anterior to nostrils left side with 23 (~19–~24) ventral spines, right side with 23 (~19–~23), anterior to barbel origin left side with 13 (~11–~14) ventral spines, right side with 12 (~12–~13); one enlarged ventral spine, distinctly larger than the other ventral spines, present just in front of each nostril. Large rostral teeth (Fig 4a and 4b) with elongated crown and oval-shaped base, slightly bent to the rear and flattened towards the apex, forming anterior and posterior cutting edges at front and rear, the latter serrated by barbed hooks. Crown base with numerous short longitudinal ridges forming a pronounced transversal crest. Both, anterior and posterior faces of the root are curved outwards from the junction of crown and root towards the base of the root. The basal face shows a deep v-shaped median groove that is antero-posteriorly directed and has an oval-shaped cavity in the center. Interstitial rostral teeth (Fig 4c–4h) with blade-shaped crown and without serration (large interstitial rostral teeth serrated and similar to large lateral rostral teeth in all specimens larger than the holotype). Crown of ventral spines (Fig 4i and 4j) elongated cone-shaped with a pronounced transversal basal ridge, root with roundish and pedestal-like base. The basal face has a large and deep roundish foramen in the center.

**Fig 5:**
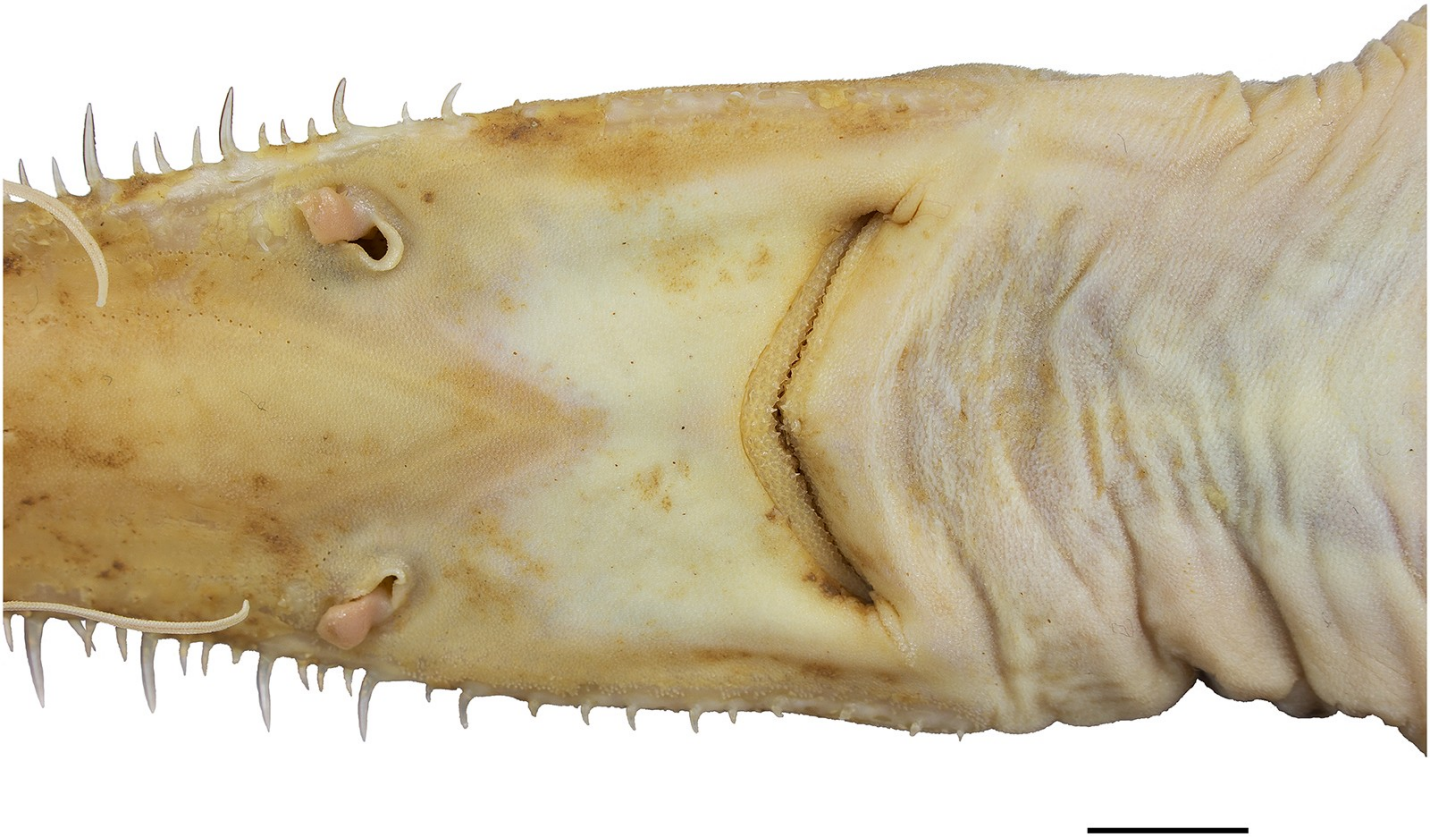
*Pliotrema kajae* sp. nov., holotype, MNHN 1987–1266, juvenile female, 560 mm TL, preserved, mouth-nasal region. Scale bar: 1 cm.

**Fig 6:**
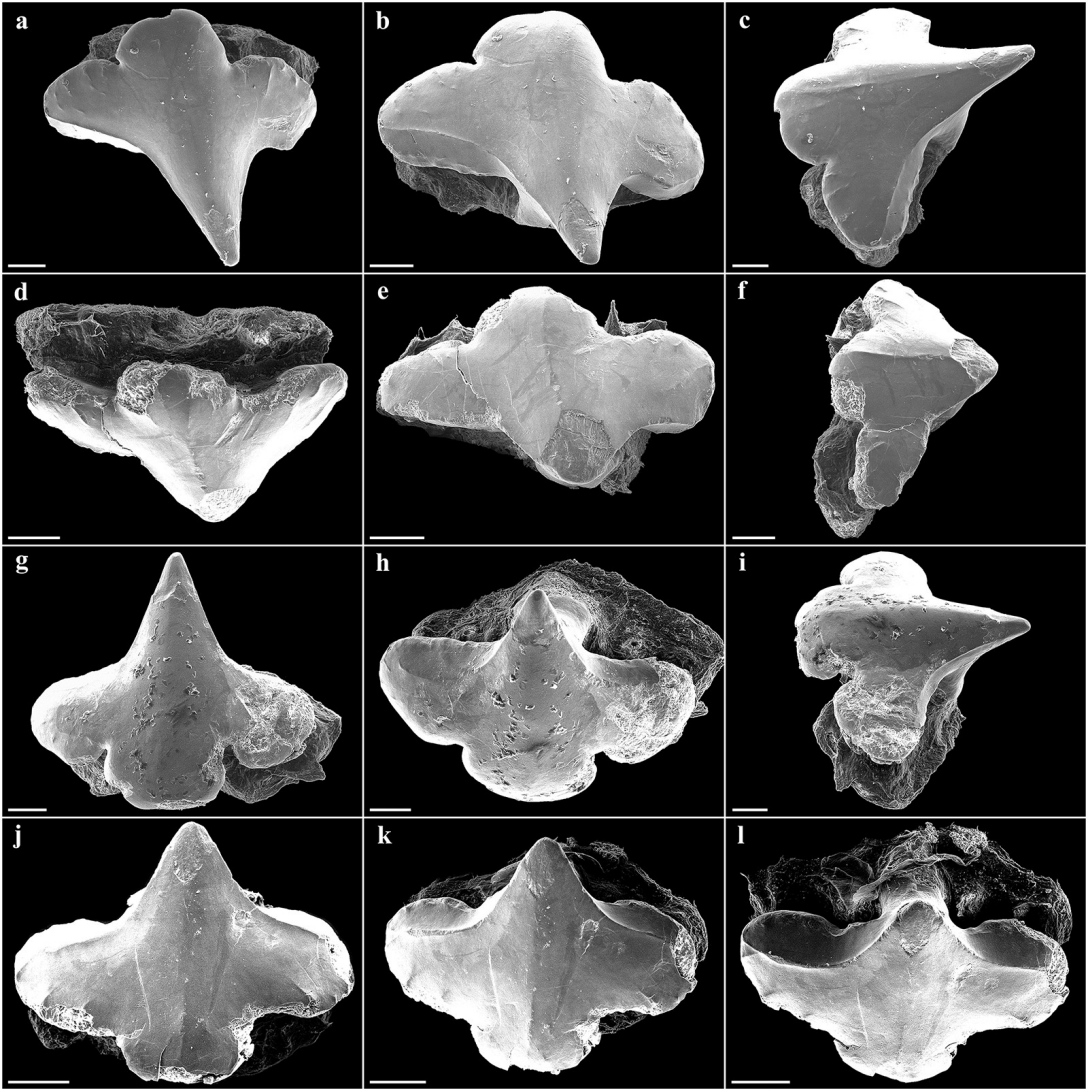
*Pliotrema kajae* sp. nov., paratype, SAIAB 84096, adult male, 940 mm TL, SEM images of oral teeth. **a**–**c** upper anterolateral tooth in **a** oblique-labial and **b**,**c** occlusal views; **d**–**f** upper posterolateral tooth in **d** oblique-labial and **e**,**f** occlusal views; **g**–**i** lower anterolateral tooth in **g** oblique-labial and **h**,**i** occlusal views; **j**–**l** lower posterolateral tooth in **j** oblique-labial and **k**,**l** occlusal views. Scale bars: 200 μm.

**Fig 7:**
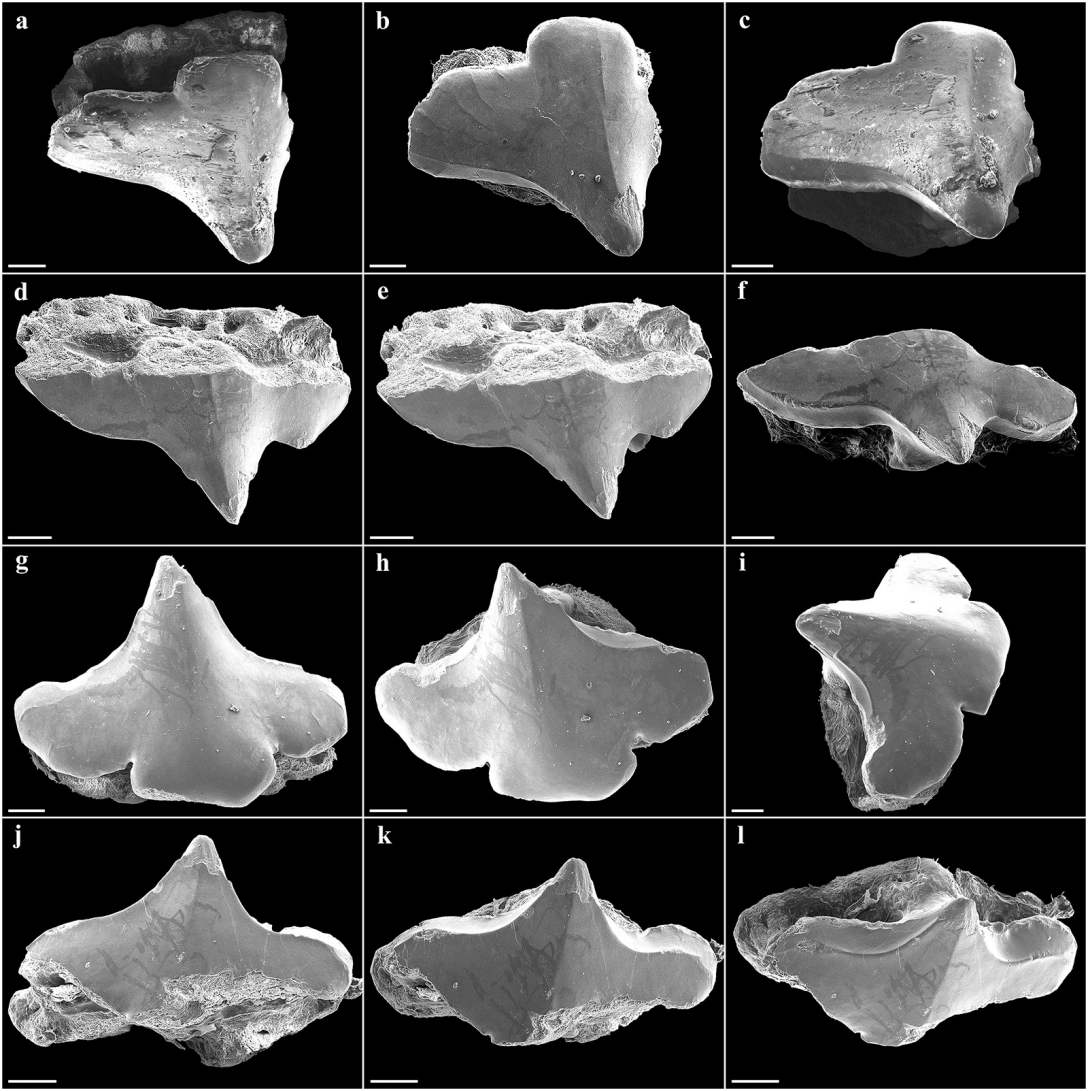
*Pliotrema kajae* sp. nov., paratype, SAIAB 84039, gravid female, 1143 mm TL, SEM images of oral teeth. **a**–**c** upper anterolateral tooth in **a** oblique-labial and **b**,**c** occlusal views; **d**–**f** upper posterolateral tooth in **d**,**e** oblique-labial and **f** occlusal views; **g**–**i** lower anterolateral tooth in **g** oblique-labial and **h**,**i** occlusal views; **j**–**l** lower posterolateral tooth in **j** oblique-labial and **k**,**l** occlusal views. Scale bars: 200 μm.

Eyes lateral on head, large, oval, length 3.3 (2.8–5.2)% TL; skeletal interorbital space 0.9 (0.7–1.0) times eye length, 9.3 (6.1–9.5) times in horizontal preorbital length; posterior eye notches and suborbital grooves present. Spiracles moderately large, length 1.4 (0.8–1.6)% TL and 0.4 (0.2–0.6) times eye length, left spiracle with 12 (12–15) folds, right one with 13 (12–15); spiracles strongly crescentic, oblique, directed posteroventrally from top to bottom, located just posterior to posterior eye notch, separated by a narrow but deep vertical groove along posterior margin of orbit, shorter than eye; upper edge below level of top of eye. Gill slits small, upright, weakly pleated, lateral on head, close to ventral surface, extending slightly onto ventral surface, subequal in length, sixth slit arches around pectoral-fin origin. Mouth large, strongly inferior, broadly arched, symphysis about level with posterior edge of eye, width 4.5 (4.4–5.4)% TL and 1.5 (1.3–1.8) times in head width; upper labial furrows absent, lower furrows short, 0.4 (0.4–0.5)% TL; corner of mouth partly concealed by lateral muscles of jaw (Fig 5). Teeth unicuspidate, in well-defined series, bases oval and flattened with short but pronounced, narrow median cusp near middle of jaw, no lateral cusps (cusps similar in paratypes except for adult male SAIAB 84096, which has distinctly longer cusps); cusps diminishing in height towards jaw angles, indistinct near jaw corners; about 4–5 series of functional teeth (Figs 6 and 7). Median cusp with labial face slightly convex and with both mesial and distal cutting edges weakly bent mesially and distally in occlusal view, respectively. The mesial and distal crown base parts somewhat curve apically. A pronounced and broad, irregularly shaped apron overlaps the junction of crown and root, building a notch at the junction with both mesial and distal crown base parts. Basal ornamentation, striae or reticulations absent, but sharp folds present in both upper and lower jaw teeth. The lingual face of the cusp is strongly convex, a well-developed uvula is present at the central crown base. The mesial/distal latero-lingual crown faces curve strongly towards the apex of the crown, partially forming a sharp notch with the uvula (Fig 6k and 6l). The root is anaulacorhizid and slightly arched without lobation. The outer surface of the root shows up to five large basal foramina, which are mostly oval-shaped. The inner face of the root shows up to six well-developed foramina along the crown-root junction at each side of the uvula. The basal face of the root is flat, partly showing some outer foramina.

Nostrils small, widely separated, subcircular; nostril width 0.8 (0.7–1.0)% TL, 4.5 (3.8–5.6) times in internarial width, 5.7 (4.8–7.0) times in mouth width, 7.7 (6.4–9.6) times in width of rostrum at nostrils; located distinctly forward of level of anterior margin of eye; distance from anterior nostrils to symphysis of upper jaw 1.3 (1.2–1.5) times internarial space, distance from barbel origin to anterior nostrils 10.4 (8.5–10.5)% TL. Anterior nasal flaps well developed, leaf-like, extended ventrally beyond nostrils; incurrent and excurrent apertures surrounded by pronounced marginal lobes; no nasoral or circumnarial grooves; no dermal lobes (Fig 5).

**Fig 8:**
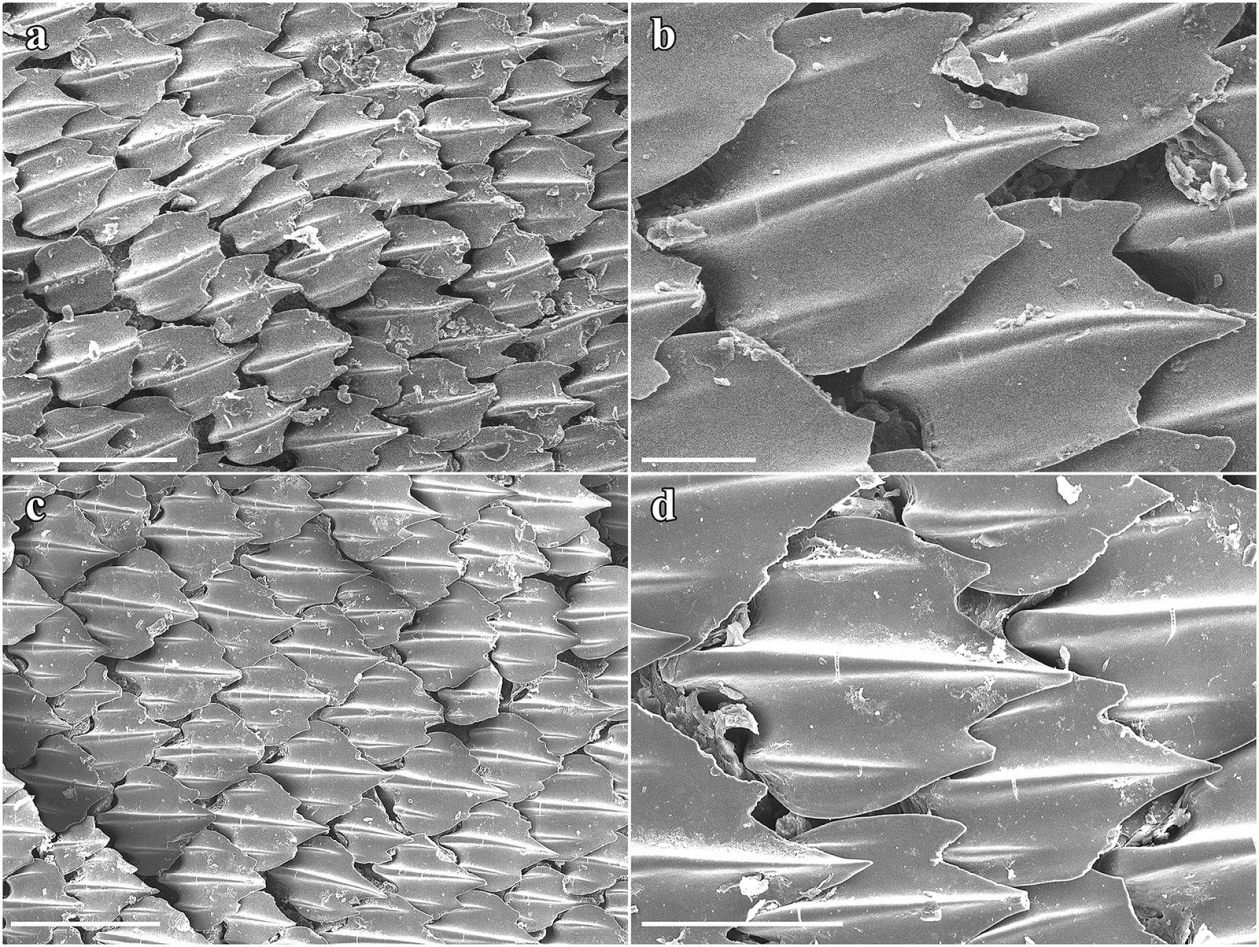
*Pliotrema kajae* sp. nov., SEM images of lateral trunk dermal denticles in apical views. **a**,**b** paratype, SAIAB 84096, adult male, 940 mm TL, **c**,**d** paratype, SAIAB 84039, gravid female, 1143 mm TL. Scale bars: **a**,**c** 500 μm, **b** 100 μm, **d** 200 μm.

**Fig 9:**
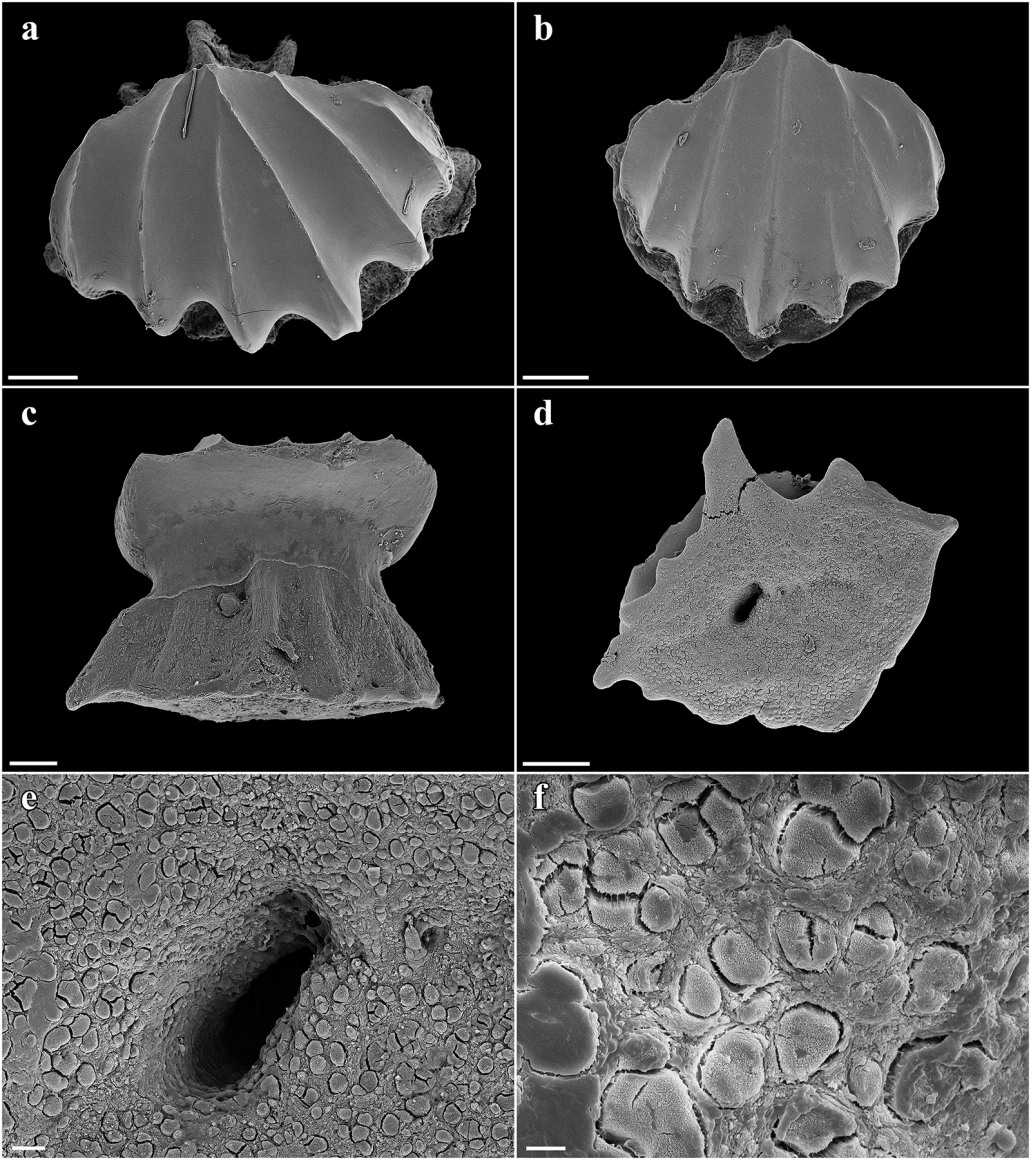
*Pliotrema kajae* sp. nov., paratype, RHL-Mad-01, presumably adult female, estimated TL 1100 mm, SEM images of single rostral dermal denticles. **a**,**b** apical views, **c** lateral view, **d**–**f** basal **d** total and **e**,**f** close-up views. Scale bars: **a**,**b**,**d** 100 μm, **c** 50 μm, **e** 10 μm, **f** 3 μm.

Lateral trunk dermal denticles densely set and overlapping, with flat, tricuspidate crowns (Fig 8). The lateral cusps are rather weakly pronounced but situated quite far anteriorly so that the median cusp is not much longer than the lateral cusps. The median ridge is strongly pronounced and reaches the tip of the median cusp. The lateral ridges are less pronounced and do not reach the tips of the lateral cusps. The surface of the denticles is only weakly structured by reticulations very close to base. No sexual dimorphism detectable in the morphology of the trunk dermal denticles. Dermal denticles on rostrum fan-shaped, with an obtusely angled, weakly pronounced median cusp and no lateral cusps but with 6–7 strongly pronounced ridges (Fig 9). The surface of the rostral dermal denticles is only weakly structured by reticulations very close to base (Fig 9a–9c).

**Fig 10:**
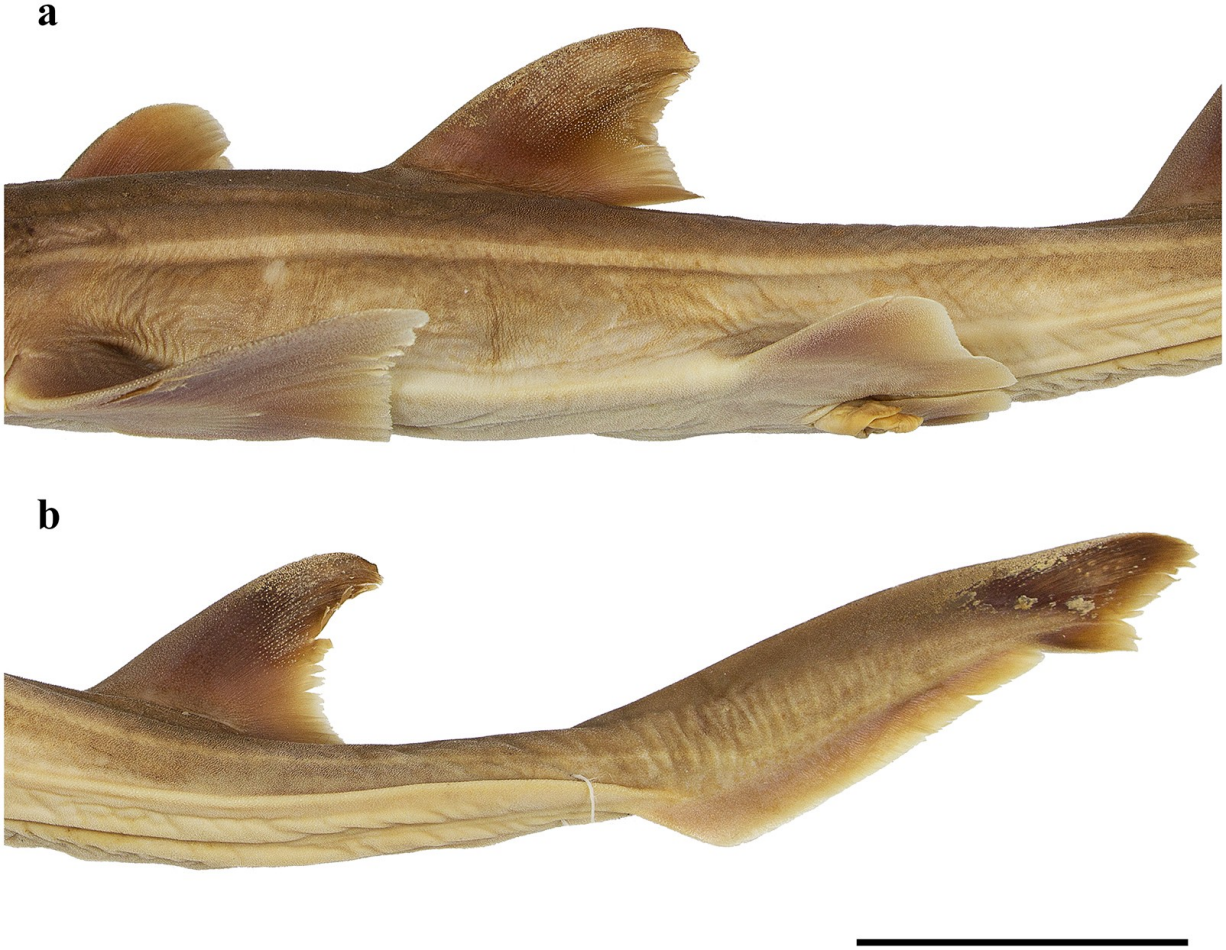
*Pliotrema kajae* sp. nov., holotype, MNHN 1987–1266, juvenile female, 560 mm TL, preserved, lateral views of fins. **a** pectoral, first dorsal, and pelvic fins, **b** second dorsal and caudal fins. Scale bar: 5 cm. Note ventral precaudal ridge in **b**.

Pectoral fins large, anterior margin weakly convex, 11.4 (10.3–12.2)% TL and 1.5 (1.4–2.0) times inner margin; apex narrowly rounded; posterior margin weakly concave, directed across horizontal axis at about origin of first dorsal fin; inner margin convex and strongly notched basally; free rear tip angular (Figs 3 and 10a). Pelvic fins moderately large, anterior margin almost straight to slightly convex, 6.5 (5.3–6.7)% TL, 1.6 (1.6–1.9) times in first dorsal-fin anterior margin, and 1.5 (1.3–1.7) times in second dorsal-fin anterior margin; apex narrowly rounded; posterior margin concave; inner margin weakly convex and slightly notched basally; free rear tip broadly rounded; origin distinctly posterior to level free tip of first dorsal fin and well forward of level second dorsal fin origin (Fig 10a).

First dorsal fin broad, semifalcate, anterior margin slightly convex; apex narrowly rounded; posterior margin slanting posteroventrally, slightly convex distally, strongly concave in basal three quarters; inner margin straight, free rear tip narrowly pointed; origin about opposite pectoral-fin free rear tips; insertion and free rear tip clearly anterior to level pelvic-fin origins (Fig 10a). Second dorsal fin somewhat smaller than first but of similar shape, anterior margin weakly convex, apex very narrowly rounded; posterior margin weakly convex distally, strongly concave near basal three quarters; inner margin straight, free rear tip narrowly pointed; origin clearly behind level pelvic insertions; interdorsal space 1.4 (1.3–1.7) times first dorsal-fin length, 1.7 (1.3–1.9) times dorsal–caudal space; second dorsal-fin inner margin 1.0 (0.7–1.2) times subterminal caudal-fin margin (Fig 10b).

Caudal fin short, dorsal margin slightly convex, length 18.8 (17.1–19.9)% TL, 1.1 (0.9–1.3) times in pelvic–caudal space and 5.1 (3.9–7.9) times terminal caudal margin; lower post-ventral lobe absent, upper post-ventral margin slightly convex; terminal lobe well developed, caudal terminal margin slightly concave, apices angular (Fig 10b). Ventral origin of caudal fin situated anteriorly due to low anterior fin ridge (Fig 10b).

**Fig 11:**
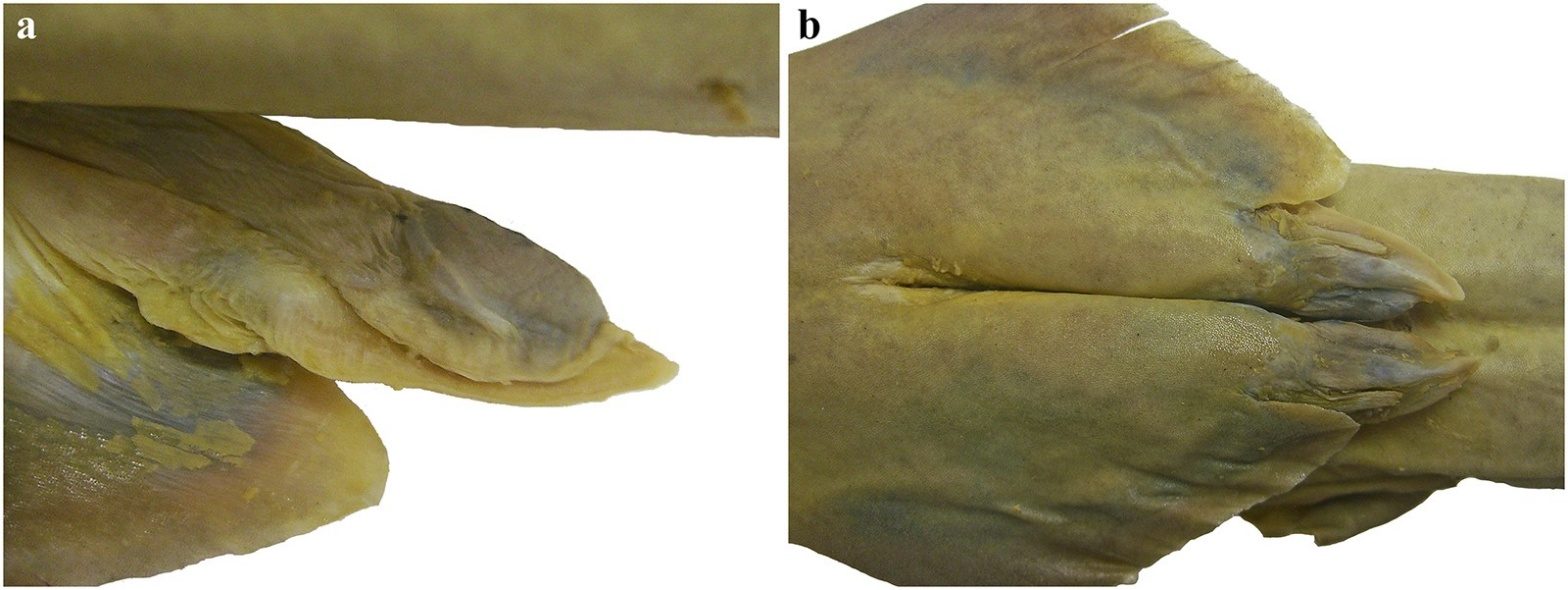
*Pliotrema kajae* sp. nov., paratype, SAIAB 84096, adult male, 940 mm TL, preserved, claspers. **a** left clasper in dorsal view, **b** right and left claspers in ventral view.

Clasper morphology: Fig 11 provides photographs of the claspers of adult male SAIAB 84096. The claspers of adult males extend posteriorly to clearly posterior to level of pelvic-fin free rear tips. Clasper shaft flattened rod-shaped. Glans broad and flattened, with long, straight and thorn-like spur. Due to the fragile condition of the specimen, it was not possible to open one of the claspers for further examination.

**Fig 12:**
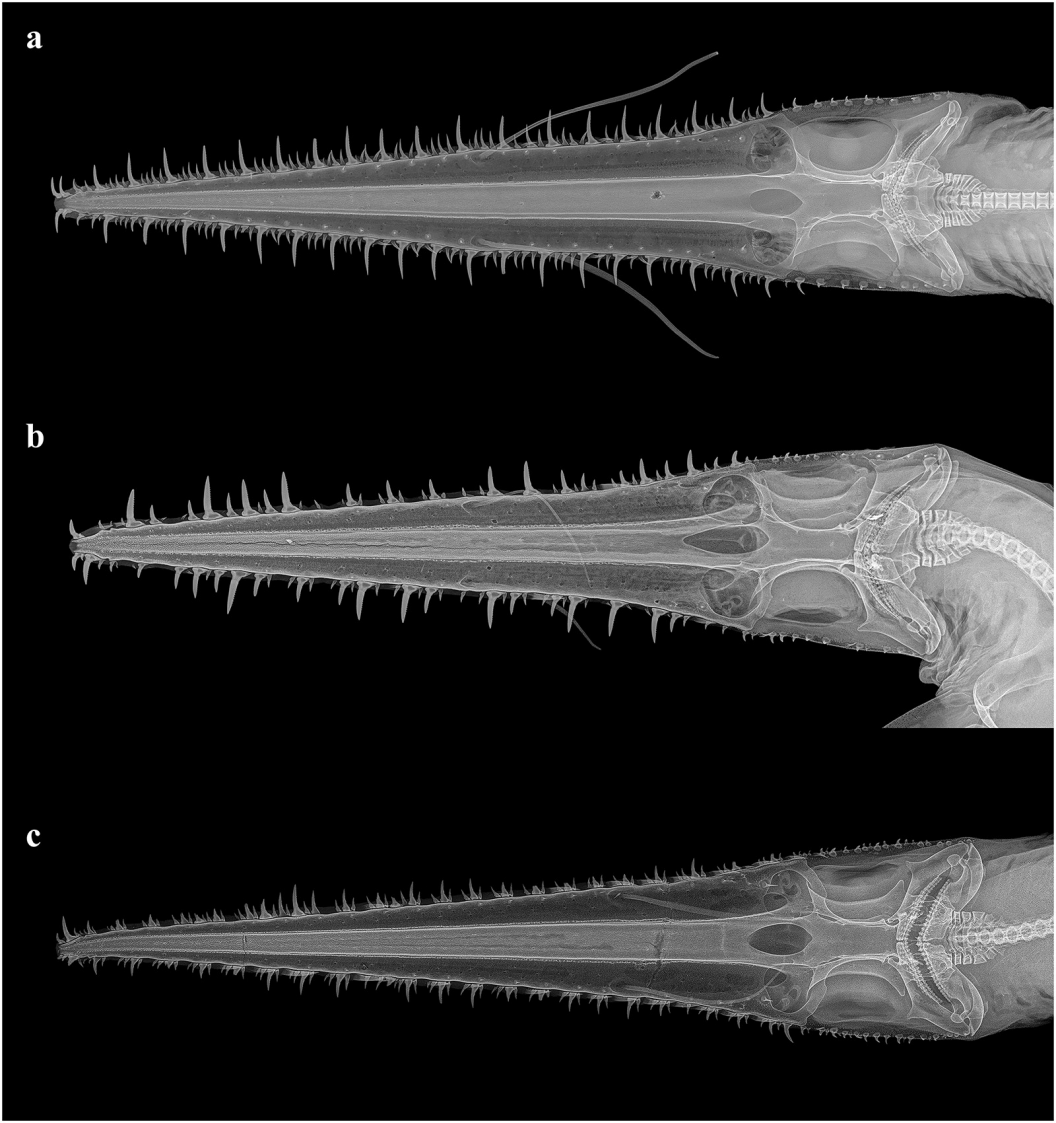
Radiographs of the crania of the three species of *Pliotrema* in dorsal views. **a** *P*. *kajae* sp. nov., holotype, MNHN 1987–1266, juvenile female, 560 mm TL (image merged from two radiographs), **b** *P*. *annae* sp. nov., holotype, ZMH 26361, presumably adult female, 981 mm TL, **c** *P*. *warreni*, DMM I-E/4946, female, 785 mm TL.

Cranium: five anterior-most basiventral cartilages laterally expanded, with curved, dorsally reflected margins. Chondrocranium and cranial nerves highly modified to accomodate the elongated rostrum. Foramen magnum surrounded by crescent-shaped occipital condyles. Dorsal fenestra of the precerebral fossa teardrop-shaped, with posterior notch (Fig 12a).

Skeletal meristics (from radiographs): monospondylous trunk vertebral centra: 57 (52–56); diplospondylous precaudal centra: 49 (48–56); total precaudal centra: 106 (101–110); caudal centra: 54 (47–54); total centra: 160 (151–164).

**Fig 13:**
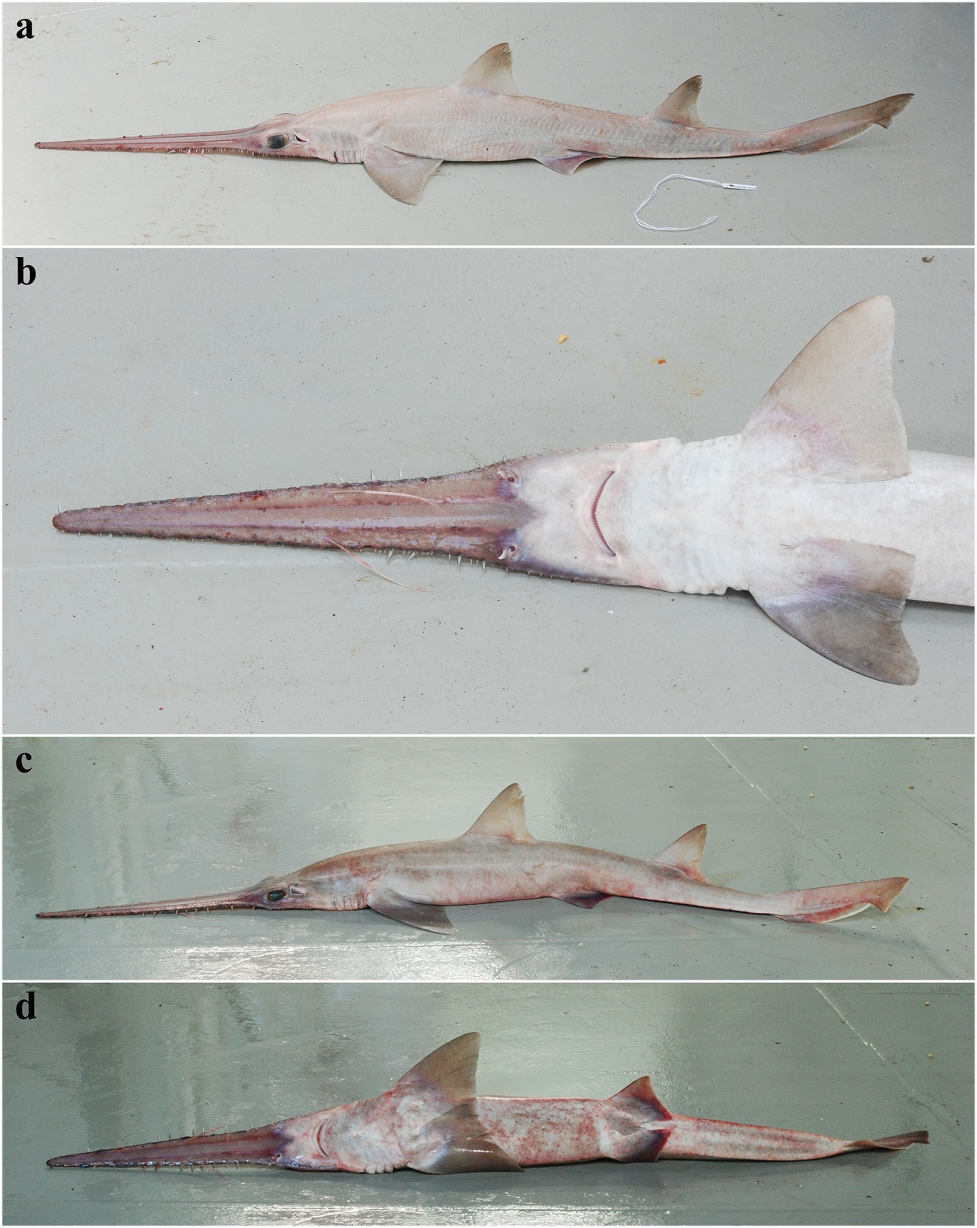
*Pliotrema kajae* sp. nov., fresh images of paratypes. **a**,**b** paratype SAIAB 84096, adult male, 970 mm TL fresh, **c**,**d** paratype SAIAB 84039, gravid female, 1170 mm TL fresh. The photographs were taken and kindly provided by Oddgeir Berg Alvheim, Institute of Marine Research, Bergen, Norway.

Coloration. Fresh, prior to preservation (paratypes SAIAB 84039 and SAIAB 84096; Fig 13): ground color pale (SAIAB 84096) to light brown (SAIAB 84039) dorsally with two thin yellowish longitudinal stripes (hardly detectable in paratype SAIAB 84096); uniform white ventrally; fins translucent dusky, upper post-ventral caudal-fin and pelvic-fin posterior margins narrowly edged white, weak white edges also present at posterior margins of pectoral and dorsal fins, as well as terminal caudal-fin margin; rostrum translucent dusky, dark edged and with two distinct longitudinal stripes dorsally; lateral rostral teeth dark-edged; ventrolateral keels white. Color in preservative (holotype and paratypes; Figs 1 and 2): coloration similar to fresh coloration but specimen SAIAB 84096 with formerly pale dorsal coloration somewhat darker dorsally, ventral coloration uniform yellowish instead of white as usual, ventrolateral keels also yellowish; dark edging of rostrum and lateral rostral teeth, as well as longitudinal dorsal rostral stripes still conspicuous.

#### Size

A large sawshark species reaching about 1430 mm TL based on photographs of an adult female kindly provided by Blue Ventures along with photographs of an adult male of 1020 mm TL. The female was caught on 19 July 2015 and landed at Andranombala, Madagascar, whilst the male was caught on 22 September 2015 and landed at Andavadoaka, Madagascar. Both specimens were not preserved. The male paratype SAIAB 84096 is adult at 970 mm TL fresh, 940 mm TL preserved, the female paratype SAIAB 84039 is gravid at 1170 mm TL fresh, 1143 mm TL preserved, containing about six eggs (based on radiographs). The size at birth is estimated at around 350 mm TL based on the four near-term embryos of 318 to 329 mm TL.

#### Distribution

**Fig 14:**
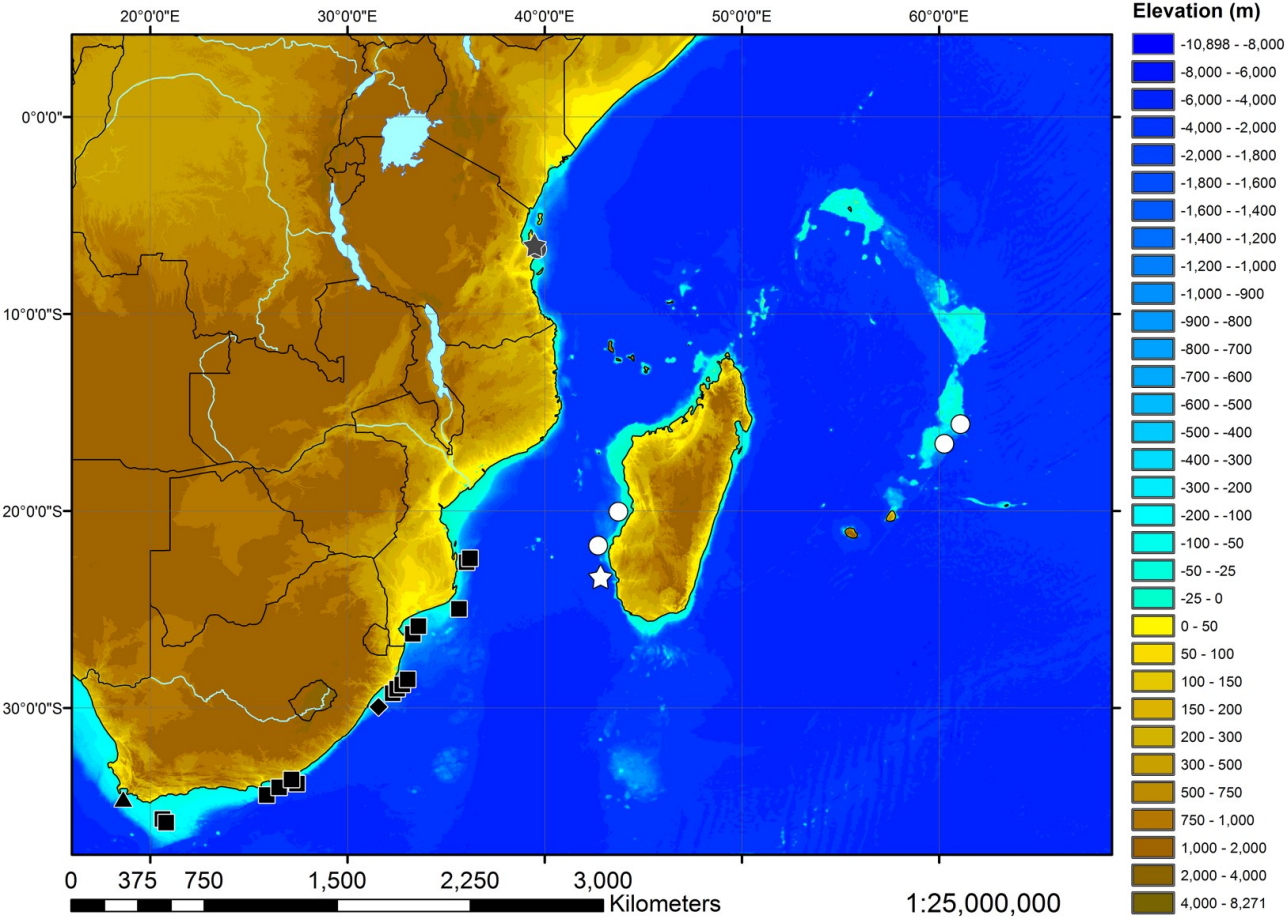
Map of the southwestern Indian Ocean depicting the catch locations of the examined specimens of all three species of *Pliotrema*. Holotype (white star) and paratypes (white circles) of *Pliotrema kajae* sp. nov., holotype (gray star) and paratype (gray circle) of *Pliotrema annae* sp. nov., and intact (black diamond) and dissected (black triangle) syntypes, as well as other specimens (black squares) of *Pliotrema warreni*.

Known from off Madagascar and the Mascarene Ridge in depths from 214 to 320 m (Fig 14). The depth range of 425–500 m, given for the holotype of *Pliotrema kajae* sp. nov. by Séret [26] and Compagno et al. [27] is erroneous. *Pliotrema kajae* sp. nov. is apparently the only species of the genus occurring in this area.

#### Etymology

The new species is named after Kaja Magdalena Weigmann, the daughter of the first author, who had her first contact with chondrichthyan taxonomy when observing with great interest the examination of *Pliotrema* specimens for the present study. The name “Kaja” also has the Frisian meaning “warrior”, referring to the saw-like rostrum.

#### Remarks

There are several morphometric differences between the embryos and the larger type specimens of *Pliotrema kajae* sp. nov., which might be of ontogenetic nature, most notably, the barbel length. The differences are demonstrated in Table 2. Further ontogenetic differences can be found in the morphology of the lateral interstitial rostral teeth. In specimens larger than the juvenile holotype, larger interstitial teeth are serrated, similar to the large lateral rostral teeth. In smaller specimens up to at least 560 mm TL all interstitial teeth are unserrated.

**Table 2:** *Pliotrema kajae* sp. nov., morphometric (in % TL) and meristic differences between embryonic (n = 6) and larger (n = 3) type specimens (most pronounced differences marked in bold).

	Range embryos (n = 6)	Range larger types (n = 3)	Mean embryos (n = 6)	Mean larger types (n = 3)
**EYL, eye length**	**4.1–5.2**	**2.8–3.3**	**4.5**	**3.0**
EYH, eye height	1.8–2.9	1.2–1.4	2.2	1.3
ING, intergill length 1st to last slit	4.0–4.9	2.6–3.9	4.3	3.2
P1P, pectoral posterior margin length	6.7–8.5	8.4–9.9	7.7	9.2
P1H, pectoral height, base end to tip	8.9–10.1	10.4–11.4	9.7	10.8
D2H, D2 vertical height	4.5–5.8	5.8–6.2	5.1	6.1
D2P, D2 posterior margin length	3.7–5.1	5.6–5.8	4.6	5.7
MOL, mouth length (arc radius)	1.0–1.3	0.9–0.9	1.2	0.9
MOW, mouth width	4.7–5.4	4.4–4.6	5.1	4.5
INW, internarial width	3.8–4.3	3.1–3.5	4.1	3.3
ANF, anterior nasal flap length	1.2–1.6	0.7–0.9	1.4	0.8
INOI, interorbital space, integumental	5.2–5.9	4.1–4.5	5.4	4.3
INOS, interorbital space, skeletal	3.3–4.2	2.7–3.0	3.5	2.9
SPL, spiracle length	0.8–1.4	1.4–1.6	1.1	1.5
**BAL, Barbel length**	**15.3–18.1**	**6.6–9.9**	**16.8**	**7.8**
INS, Interspiracular space	4.9–5.7	4.1–4.7	5.3	4.4
RWN, Rostral width at anterior nostrils	6.9–7.5	5.3–6.0	7.2	5.7
RWB, Rostral width at origin of barbels	4.5–5.1	3.6–4.2	4.8	3.8
**RTAL, Rostral tooth length (anterior of nostrils): Length of longest tooth immediately anterior to barbel**	**2.1–2.3**	**0.9–1.1**	**2.2**	**1.0**
RTAW, Rostral tooth width (anterior of nostrils): Width of exposed base of above tooth	0.3–0.4	0.2–0.2	0.3	0.2
**total large lateral rostral teeth l./r**.	**28–31/28–31**	**21–23/21–23**	**28.6/28.8**	**22.0/22.0**
**large lateral rostral teeth posterior to barbels l./r**.	**15–17/15–17**	**8–10/8–10**	**15.6/15.4**	**9.0/9.0**

#### *Pliotrema annae* Weigmann, Gon, Leeney & Temple sp. nov.

urn:lsid:zoobank.org:act:DE91D0E9-3AED-41FC-8CB6-09A53E50EEBD.

Proposed English vernacular name: Anna’s sixgill sawshark.

Proposed German vernacular name: Annas Sechskiemer-Sägehai.

Local name: Papa Unguja.

Figs 15–25; Table 3.

*Pliotrema warreni*: Gubanov [28]: 221 (in part)?

The holotype and paratype are deposited in the Zoological Museum Hamburg (ZMH).

Holotype **ZMH 26361**, presumably adult female, 981 mm TL fresh, caught on or near Kobela Reef (~6°29’35”S 39°22’21”E) in Menai Bay, Unguja Island, Zanzibar, in a demersal longline at ~20–25 m (i.e. set depth of the gear; water depth unknown but probably only 2–5 m deeper than this), haul time ~8 am, time of catch unknown but likely during hours of darkness, date 07 March 2019.

Paratype **ZMH 26362**, presumably adult female, 980 mm TL fresh, 950 mm TL 70% ethanol preserved, caught off Zanzibar in a longline at ~25–35 m depth during hours of darkness, landed on 24 Feb 2017 in Kizimkazi-Dimbani, Zanzibar (two further specimens were landed at the same place but not retained on 23 Jan 2017: gravid female, ~980 mm TL, with six eggs and on 25 Jan 2017: female with saw cut off, ~580 mm to beginning of saw; both these specimens were also caught in a longline at ~25–35 m depth during hours of darkness). Measurements taken from the fresh photographs of the not retained gravid female show that this specimen can be assigned to *P*. *annae* sp. nov. based on the main morphometric characteristics, particularly the generally shorter snout.

#### Diagnosis

A medium-sized six-gilled sawshark with the following characters: barbel origin to anterior nostrils 1.9–2.0 times anterior nostrils to symphysis upper jaw; prenarial length 1.6–1.7 times prebarbel length; preoral length 1.5–1.7 times interdorsal space; pectoral-fin anterior margin 1.4–1.5 times dorsal–caudal space; mouth width 2.7–3.2 times spiracle length. First dorsal fin originates about opposite pectoral-fin free rear tips. Lateral trunk dermal denticles tricuspidate, rather flat and imbricated. Color uniform medium to dark brown dorsally without longitudinal stripes; white ventrally but with few indistinct dark blotches on belly; fins with pronounced white posterior fin margins, particularly caudal and pectoral fins; dorsal rostrum surface with two distinct longitudinal dark stripes, lateral rostral teeth dark-edged. Monospondylous centra 53–54; precaudal diplospondylous centra 46–49; total vertebral centra 154. *Pliotrema annae* is distinguished from its two congeners by a combination of characteristics, including a generally shorter snout, with head length 34.2–34.5% TL, preorbital length 21.7–22.0% TL, preoral length 24.6–25.1% TL, prebarbel length 12.6–12.7% TL, and barbel origin to symphysis upper jaw 12.1–12.3% TL. *Pliotrema annae* further differs from its two congeners in lower total large lateral rostral tooth and ventral rostral spine counts, and a rostrum that is slightly constricted between barbel origin and nostrils. Like in *P*. *kajae*, the barbels are situated about half way from rostral tip to mouth, with prebarbel length about equidistant from barbel origin to symphysis of upper jaw. In contrast, the barbels are situated about two thirds way from rostral tip to mouth, with prebarbel length about twice distance from barbel origin to symphysis of upper jaw in *P*. *warreni*.

#### Description of the holotype

Values of the paratype are presented in parentheses, more complex differences between holotype and paratype are described separately. Where relevant, ratios are based on horizontal measurements unless otherwise stated. Morphometric measurements and meristics are given in Table 3.

**Table 3:** *Pliotrema annae* sp. nov., morphometrics and meristic of the presumably adult female holotype (ZMH 26361, measured in fresh, i.e. defrozen condition) and presumably adult female paratype (ZMH 26362, measured in 70% ethanol preserved). Proportional values are expressed as percentages of total length (TL).

	*Pliotrema annae* sp. nov., presumably adult female holotype, ZMH 26361		*Pliotrema annae* sp. nov., presumably adult female paratype, ZMH 26362	
	mm	% TL	mm	% TL
TL, total length	981.0	100.0	950.0	100.0
PRC, precaudal length, dorsally	795.0	81.0	765.0	80.5
PRVC, precaudal length, ventrally	790.0	80.5	760.0	80.0
PD2, pre-D2-length	645.0	65.7	620.0	65.3
PD1, pre-D1-length	425.0	43.3	415.0	43.7
HDL, head length (to end of last gill slit), horizontally	338.0	34.5	325.0	34.2
HDL, head length (to end of last gill slit), point to point	340.0	34.7	328.0	34.5
PG1, prebranchial length, horizontally	290.5	29.6	290.0	30.5
PG1, prebranchial length, point to point	293.1	29.9	292.0	30.7
PSP, prespiracular length, horizontally	242.8	24.7	243.1	25.6
PSP, prespiracular length, point to point	245.2	25.0	244.1	25.7
POB, preorbital length, horizontally	213.3	21.7	208.9	22.0
POB, preorbital length, point to point	215.1	21.9	210.1	22.1
PP1, prepectoral length, horizontally	329.0	33.5	319.0	33.6
PP2, prepelvic length, horizontally	532.0	54.2	525.0	55.3
SVL, snout–anterior vent length	554.0	56.5	542.0	57.1
IDS, interdorsal space	157.9	16.1	138.1	14.5
DCS, dorsal (D2)–caudal space	89.1	9.1	85.6	9.0
PPS, pectoral–pelvic space	186.0	19.0	189.4	19.9
PCA, pelvic–caudal space	225.3	23.0	201.7	21.2
VCL, anterior vent–caudal tip length	426.0	43.4	411.0	43.3
PRN, prenarial length, horizontally	201.4	20.5	200.4	21.1
POR, preoral length	241.2	24.6	238.0	25.1
EYL, eye length	27.3	2.8	26.1	2.7
EYH, eye height	14.7	1.5	13.4	1.4
ING, intergill length 1st to last slit	39.9	4.1	36.1	3.8
GS1, 1st gill slit height (unspread)	11.4	1.2	11.4	1.2
GS2, 2nd gill slit height	12.0	1.2	12.3	1.3
GS3, 3rd gill slit height	12.1	1.2	12.5	1.3
GS4, 4th gill slit height	11.1	1.1	11.9	1.3
GS5, 5th gill slit height	11.3	1.1	11.8	1.2
GS6, 6th gill slit height	12.7	1.3	12.4	1.3
P1A, pectoral anterior margin length	131.5	13.4	120.3	12.7
P1B, pectoral base length	29.3	3.0	28.2	3.0
P1I, pectoral inner margin length	70.4	7.2	64.6	6.8
P1P, pectoral posterior margin length	105.1	10.7	89.8	9.5
P1H, pectoral height, base end to tip	120.6	12.3	109.3	11.5
P1L, P length anterior base to posterior tip	95.7	9.8	90.1	9.5
CDM, dorsal caudal margin length	187.3	19.1	188.4	19.8
CST, subterminal caudal margin length	23.6	2.4	24.7	2.6
CSW, subterminal caudal width	26.2	2.7	23.7	2.5
CTR, terminal caudal margin length	41.1	4.2	37.9	4.0
CTL, terminal caudal lobe length	55.0	5.6	55.8	5.9
D1L, D1 total length	103.0	10.5	101.6	10.7
D1A, D1 anterior margin length	109.5	11.2	109.0	11.5
D1B, D1 base length	72.7	7.4	74.8	7.9
D1H, D1 vertical height	71.4	7.3	68.3	7.2
D1I, D1 inner margin length	29.1	3.0	29.8	3.1
D1P, D1 posterior margin length	67.8	6.9	62.9	6.6
D2L, D2 total length	87.2	8.9	88.7	9.3
D2A, D2 anterior margin length	96.7	9.9	96.3	10.1
D2B, D2 base length	62.2	6.3	60.8	6.4
D2H, D2 vertical height	67.1	6.8	65.2	6.9
D2I, D2 inner margin length	25.7	2.6	27.6	2.9
D2P, D2 posterior margin length	61.9	6.3	58.4	6.1
P2L, pelvic total length	82.7	8.4	74.7	7.9
P2A, pelvic anterior margin length	69.3	7.1	66.5	7.0
P2B, pelvic base length	46.8	4.8	41.9	4.4
P2H, pelvic height = max. width (excl. clasper)	54.8	5.6	51.1	5.4
P2I, pelvic inner margin length	37.7	3.8	33.2	3.5
P2P, pelvic posterior margin length	51.3	5.2	47.9	5.0
HDH, head height at P origin	59.1	6.0	65.4	6.9
TRH, trunk height at P base end	64.4	6.6	65.7	6.9
ABH, abdomen height at D1 base end	74.5	7.6	75.4	7.9
TAH, tail height at pelvic base end	47.7	4.9	54.2	5.7
CPH, caudal peduncle height at dorsal caudal-fin origin	18.1	1.8	19.1	2.0
DPI, D1 midpoint–pectoral base end	108.6	11.1	111.5	11.7
DPO, D1 midpoint–pelvic origin	74.3	7.6	70.6	7.4
PDI, pelvic midpoint–D1 base end	61.6	6.3	57.5	6.1
PDO, pelvic midpoint–D2 origin	88.5	9.0	75.3	7.9
MOL, mouth length (arc radius)	9.9	1.0	7.8	0.8
MOW, mouth width	39.9	4.1	41.1	4.3
ULA, upper labial furrow length	0.0	0.0	0.0	0.0
LLA, lower labial furrow length	3.4	0.3	2.8	0.3
NOW, nostril width	6.5	0.7	6.3	0.7
INW, internarial width	30.6	3.1	28.6	3.0
ANF, anterior nasal flap length	5.1	0.5	5.6	0.6
INOI, interorbital space, integumental	38.4	3.9	37.4	3.9
INOS, interorbital space, skeletal	23.6	2.4	24.1	2.5
SPL, spiracle length	14.7	1.5	12.7	1.3
ESL, eye–spiracle space	4.5	0.5	5.1	0.5
HDW, head width at middle gill slits	63.3	6.4	64.6	6.8
TRW, trunk width at P base ends	62.3	6.3	63.3	6.7
ABW, abdomen width at D1 base end	62.2	6.3	66.9	7.0
TAW, tail width at pelvic base ends	43.6	4.4	39.2	4.1
CPW, C peduncle width at dorsal caudal-fin origin	16.7	1.7	17.8	1.9
CLO, clasper outer margin length	-	-	-	-
CLI, clasper inner margin length	-	-	-	-
CLB, clasper base width	-	-	-	-
BAL, Barbel length	55.3	5.6	53.4	5.6
PBL, Prebarbel length, horizontally	123.3	12.6	120.7	12.7
BSJ, Barbel origin to symphysis upper jaw	118.8	12.1	117.1	12.3
BAN, Barbel origin to anterior nostrils	77.3	7.9	78.6	8.3
ANJ, Anterior nostrils to symphysis upper jaw	41.1	4.2	38.5	4.1
INS, Interspiracular space	38.4	3.9	37.1	3.9
RWN, Rostral width at anterior nostrils	49.7	5.1	48.4	5.1
RWB, Rostral width at origin of barbels	32.5	3.3	32.1	3.4
RTAL, Rostral tooth length (anterior of nostrils): Length of longest tooth immediately anterior to barbel	9.3	1.0	9.6	1.0
RTAW, Rostral tooth width (anterior of nostrils): Width of exposed base of above tooth	2.1	0.2	2.3	0.2
RTIS, 1° rostral tooth interspace: First complete interspace anterior to barbels	11.2	1.1	10.3	1.1
RTIL, 2° rostral tooth length: Longest complete tooth within above primary interspace	5.6	0.6	4.9	0.5
RTPL, Rostral tooth length (posterior of nostrils): Longest rostral tooth in this region	4.0	0.4	3.2	0.3
spiracle folds left/right	10/10		11/11	
total large lateral rostral teeth l./r.	17/17		17/16	
large lateral rostral teeth anterior to barbels l./r.	10/10		11/10	
large lateral rostral teeth posterior to barbels l./r.	7/7		6/6	
ventral rostral spines anterior to nostrils l./r.	15/15		15/15	
ventral rostral spines anterior to barbel origin l./r.	9/9		10/10	
tooth rows, upper jaw	37		35	
tooth rows, lower jaw	34		32	
Vtr, monospondylous trunk vertebrae centra	53		54	
dipl. VprC, diplospondylous precaudal vertebrae centra	49		46	
VprC, total precaudal vertebrae centra	102		100	
VtermC, caudal vertebrae centra	52		54	
total vertebrae centra	154		154	

**Fig 15:**
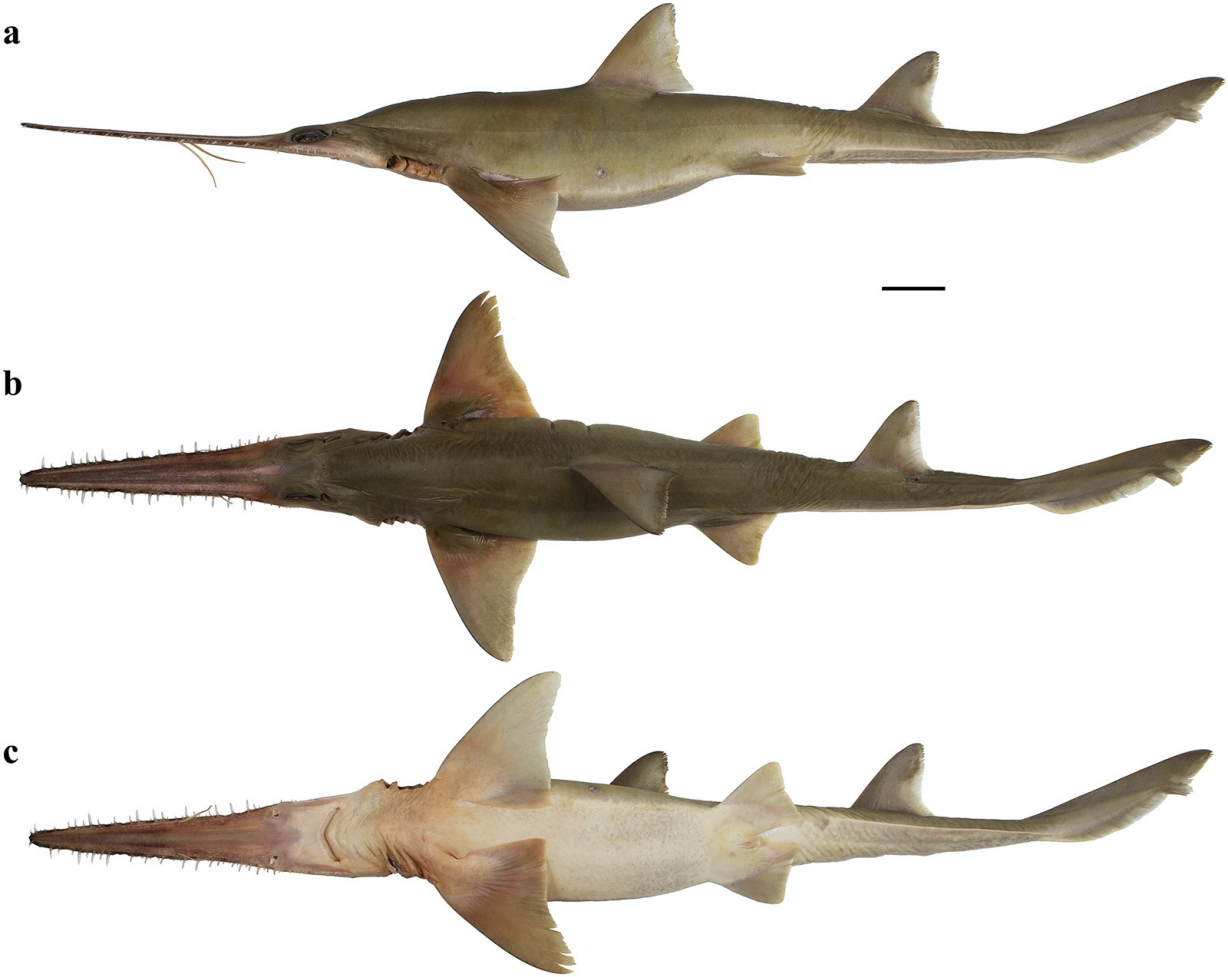
*Pliotrema annae* sp. nov., holotype, ZMH 26361, presumably adult female, 981 mm TL, in fresh (defrozen) condition. **a** lateral, **b** dorsal, and **c** ventral views. Scale bar: 5 cm.

**Fig 16:**
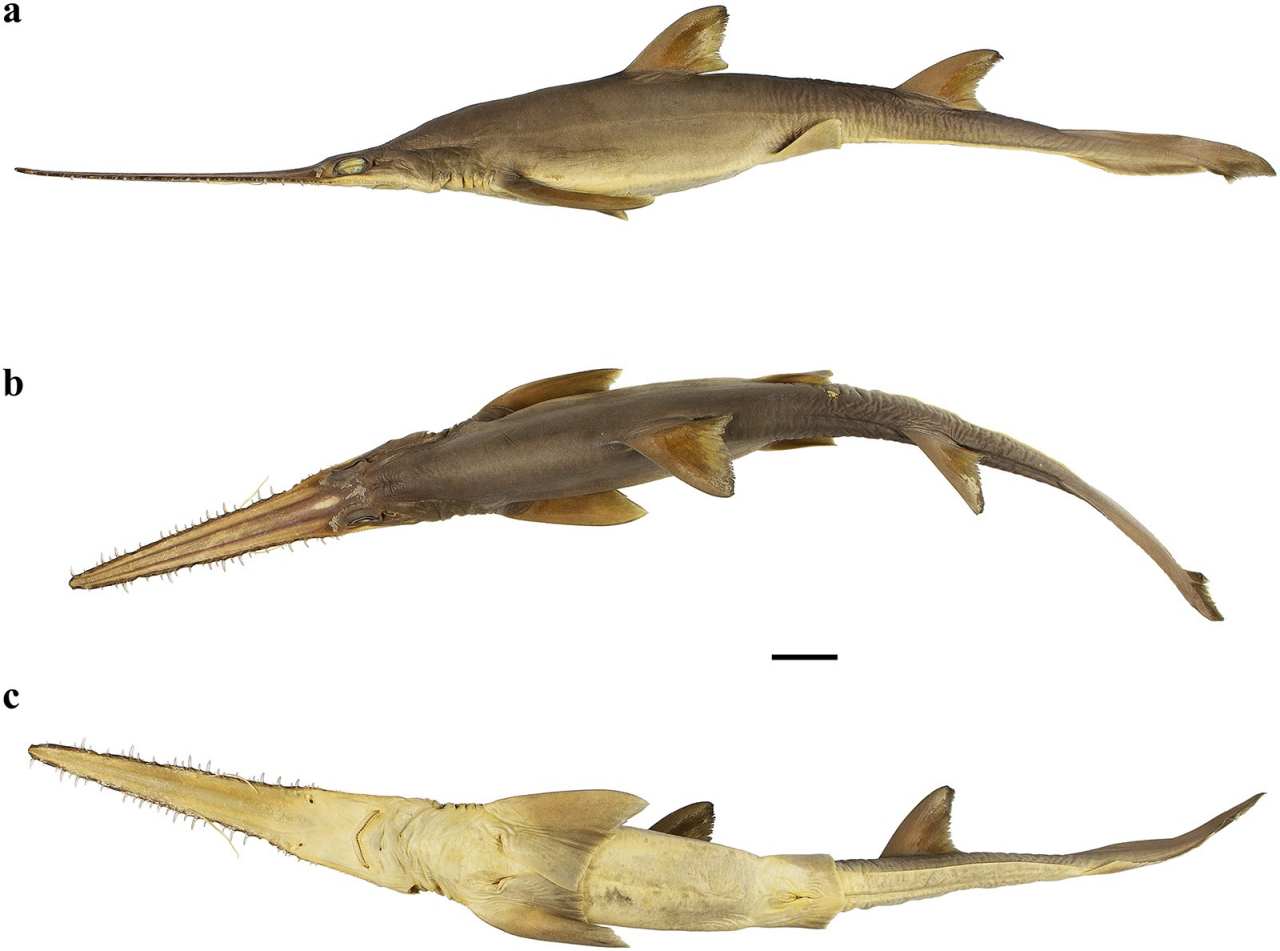
*Pliotrema annae* sp. nov., paratype, ZMH 26362, presumably adult female, 950 mm TL, preserved. **a** lateral, **b** dorsal, and **c** ventral views. Scale bar: 5 cm.

External morphology. Body firm and slender, depressed forward of gills, abdomen subcircular in cross-section, tail subtriangular in cross-section, deepest at abdomen; not tapering gradually and evenly beyond pectoral fins; snout flattened, greatly extended, saw-like; abdomen elongate, horizontal head length 0.6 (0.6) times snout–anterior vent length, pectoral–pelvic space 19.0 (19.9)% TL; pelvic–caudal space 2.7 (2.7) times pelvic-fin length; tail flattened ventrally, elongate, snout–anterior vent length 1.3 (1.3) times anterior vent–caudal tip length; caudal peduncle short, dorsal–caudal space 9.1 (9.0)% TL, caudal peduncle height 4.9 (4.5) times in dorsal–caudal space and width 1.1 (1.1) times in height; ventrolateral keels well developed, extending from somewhat behind level of free rear tip of pelvic fins to beyond origin of ventral lobe of caudal fin, converging strongly near their posterior extremity; no precaudal pit; no median predorsal, postdorsal or preventral caudal grooves (Figs 15 and 16).

**Fig 17:**
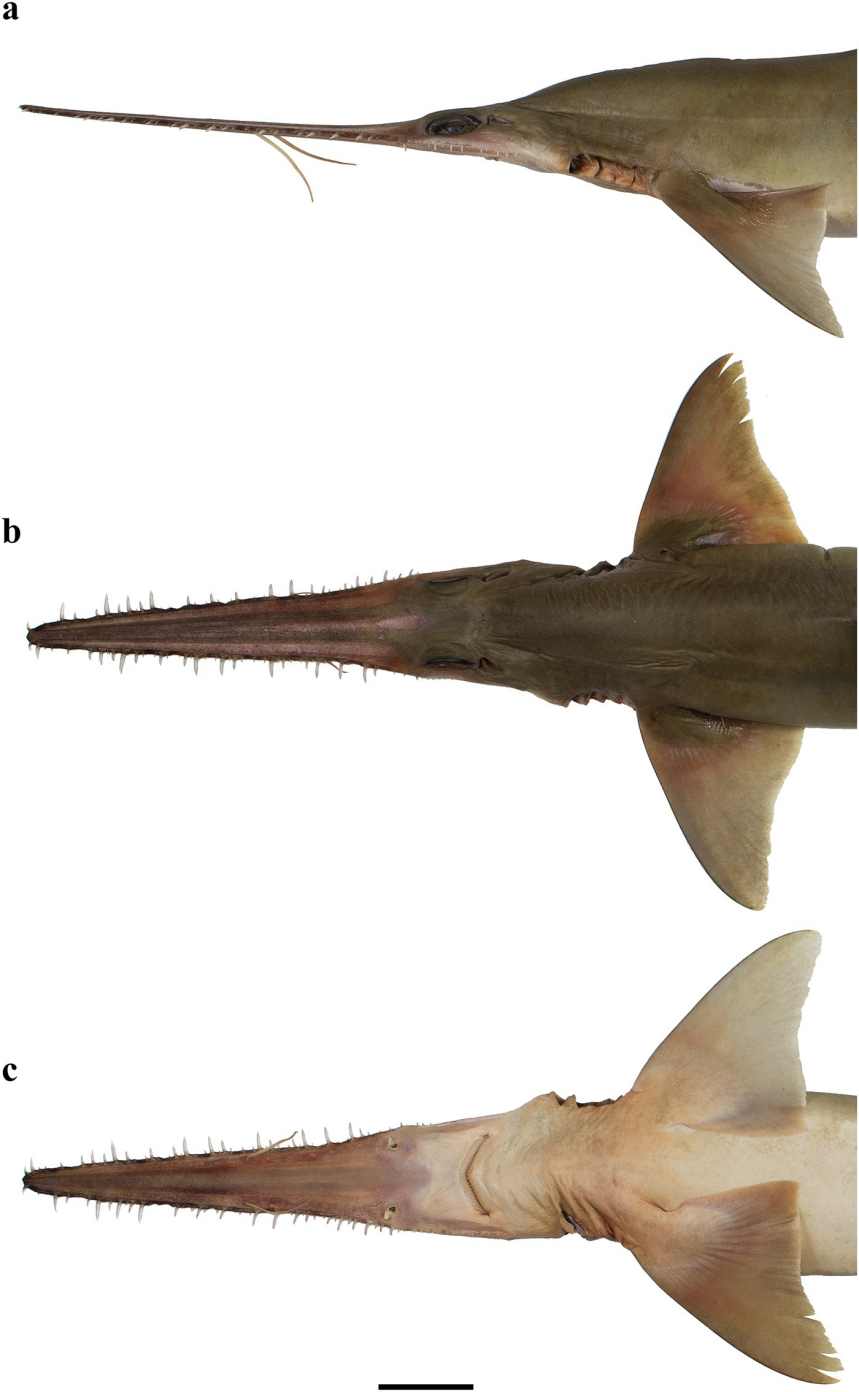
*Pliotrema annae* sp. nov., holotype, ZMH 26361, presumably adult female, 981 mm TL, in fresh (defrozen) condition. Head in **a** lateral, **b** dorsal, and **c** ventral views. Scale bar: 5 cm.

Head narrow, subtriangular and deepest at sixth gill slit, strongly depressed above eyes, head width 6.4 (6.8)% TL, 1.1 (1.0) times head height. Snout forming a very elongate, blade-like rostrum. Rostrum triangular in dorsal view; slightly constricted between barbel origin and nostrils, sides of rostrum nearly straight from tip to barbel origin but slightly concave in posterior part from barbel origin to origin of orbit; tip narrowly rounded; rostrum extending laterally below eyes as a well-defined suborbital ridge along ventrolateral edge of head, terminating somewhat behind level of posterior edge of spiracle (Fig 17).

A slender, filamentous, dorsoventrally flattened barbel originating on the ventrolateral margin about half way from rostral tip to mouth on each side, with prebarbel length 1.0 (1.0) times distance from barbel origin to symphysis of upper jaw, 51.1 (50.7)% of preoral length and 12.6 (12.7)% TL. Barbel length 2.2 (2.3) times in prebarbel length and 2.1 (2.2) times in length from barbel origin to symphysis of upper jaw. Preorbital length, horizontally 5.3 (5.1) times mouth width, 14.5 (16.4) times spiracle length, 2.1 (2.1) times first dorsal-fin length, 4.3 (4.3) times rostral width at anterior nostrils; extremely narrow in lateral view; preoral length 24.6 (25.1)% TL, 3.8 (3.7) times head width, 4.9 (4.9) times rostral width at anterior nostrils, 7.4 (7.4) times rostral width at origin of barbels, 2.0 (2.0) times prebarbel length, 1.2 (1.2) times prenarial length, and 1.5 (1.7) times interdorsal space (Fig 17).

**Fig 18:**
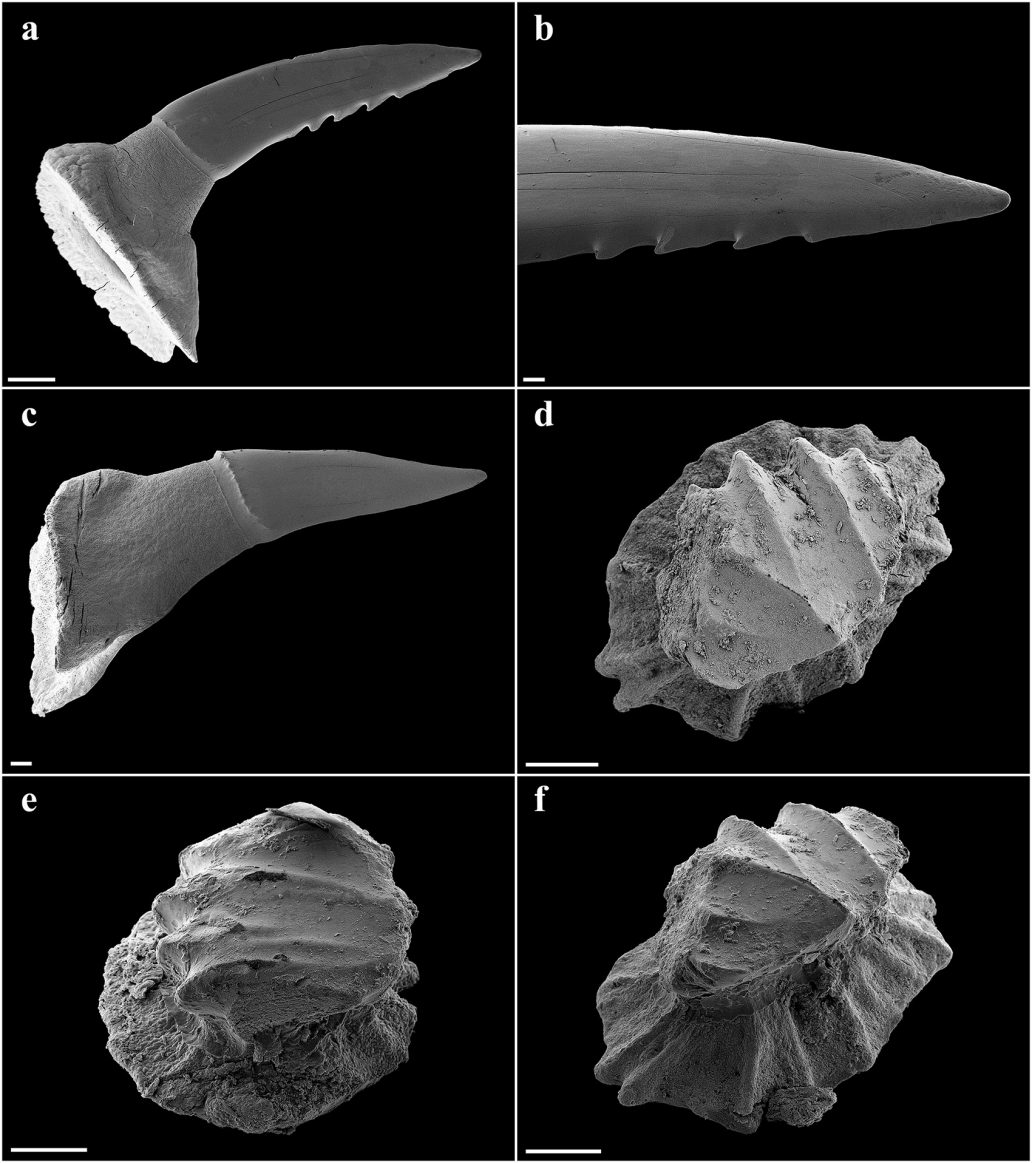
*Pliotrema annae* sp. nov., paratype, ZMH 26362, presumably adult female, 950 mm TL, SEM images of rostral teeth and rostral dermal denticles. **a**,**b** large lateral rostral tooth (image reversed) in **a** total and **b** close-up views; **c** small interstitial lateral rostral tooth in total view (image reversed); **d**–**f** rostral dermal denticles in **d** apical and **e**,**f** apico-lateral views. Scale bars: **a** 1 mm, **b**,**c** 200 μm, **d–f** 100 μm.

Large lateral rostral teeth of prenarial portion of rostrum variable in length, curved, rather stout, serrated, longest near barbel origin and near apex of rostrum posterior to anteriormost two teeth; longest tooth immediately anterior to barbels only slightly shorter than spiracle length, length 1.0 (1.0)% TL and 0.8 (0.9) times first complete interspace anterior to barbels, width 0.2 (0.2)% TL; anteriormost tooth close to tip of rostrum medium-sized, followed by a tiny tooth and the first large tooth; large teeth shortest near nostrils, longest rostral tooth posterior to nostrils 0.4 (0.3)% TL; large teeth absent behind nostrils but interstitial-like teeth present, short to very short and closely set, partially directed almost ventrally, particularly near mouth. Interspaces between large rostral teeth rather regularly sized, about as long as adjacent teeth, with 0–3 (1–3) smaller, variable interstitial teeth. Rostral tooth counts mostly symmetrical between left and right hand sides; left side with 17 (17) large teeth, right side with 17 (16); anterior to barbels left side with 10 (11) large rostral teeth, right side with 10 (10), posterior to barbels left side with 7 (6) large rostral teeth, right side with 7 (6); anterior to nostrils left side with 15 (15) ventral spines, right side with 15 (15), anterior to barbel origin left side with 9 (10) ventral spines, right side with 9 (10); one enlarged ventral spine, distinctly larger than the other ventral spines, present just in front of each nostril. Large rostral teeth (Fig 18a and 18b) with elongated crown and oval-shaped base, slightly bent to the rear and flattened towards the apex, forming anterior and posterior cutting edges at front and rear, the latter serrated by barbed hooks. Crown base with numerous short longitudinal ridges forming a pronounced transversal crest. Both, anterior and posterior faces of the root are curved outwards from the junction of crown and root towards the base of the root. The basal face shows a deep v-shaped median groove that is antero-posteriorly directed and has an oval-shaped cavity in the center. Large interstitial rostral teeth similar but with somewhat less pronounced serration. Small interstitial rostral teeth (Fig 18c) with blade-shaped crown and without serration. Crown of ventral spines elongated cone-shaped with a pronounced transversal basal ridge, root with roundish and pedestal-like base.

**Fig 19:**
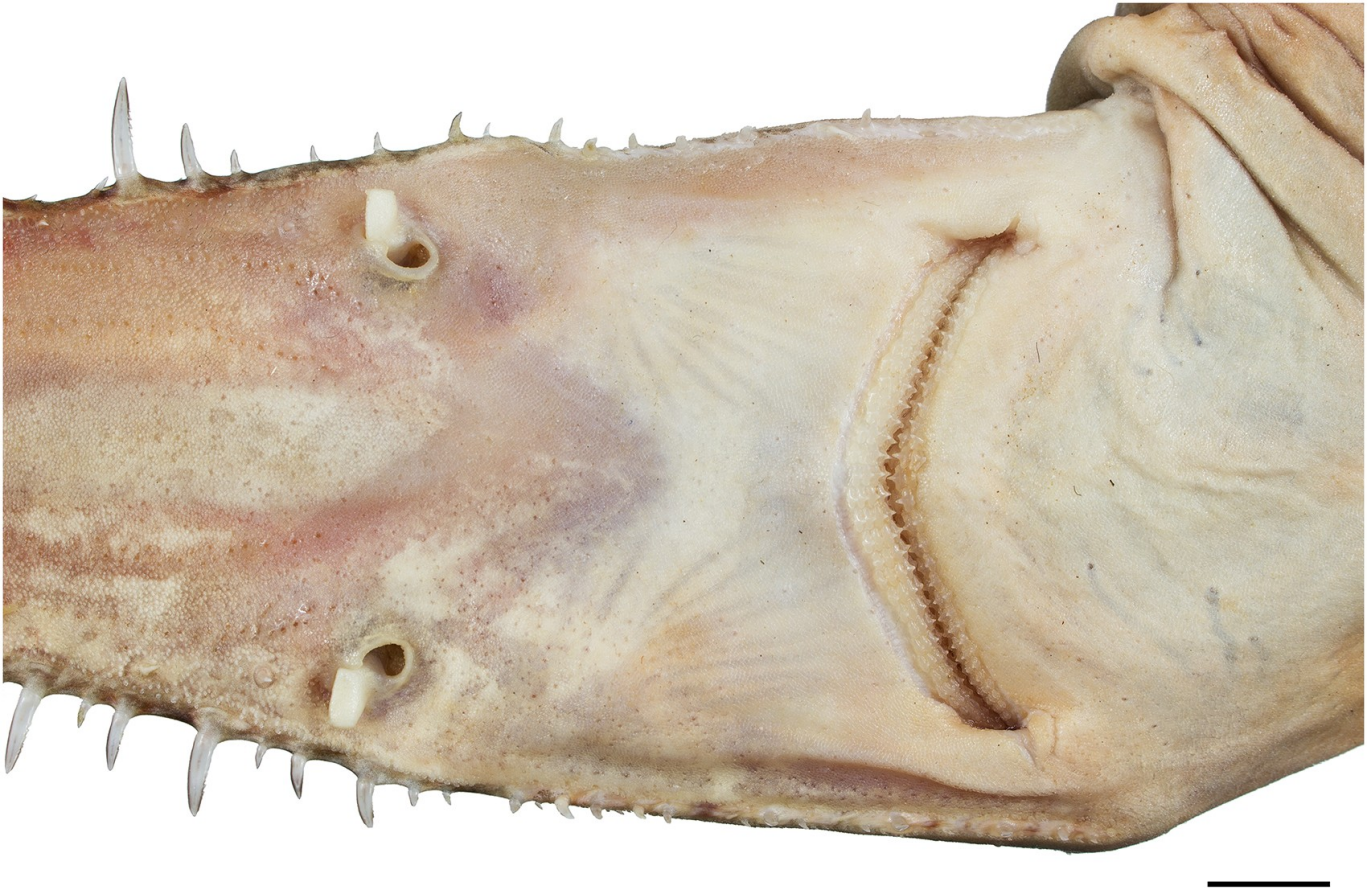
*Pliotrema annae* sp. nov., holotype, ZMH 26361, presumably adult female, 981 mm TL, in fresh (defrozen) condition, mouth-nasal region. Scale bar: 1 cm.

**Fig 20:**
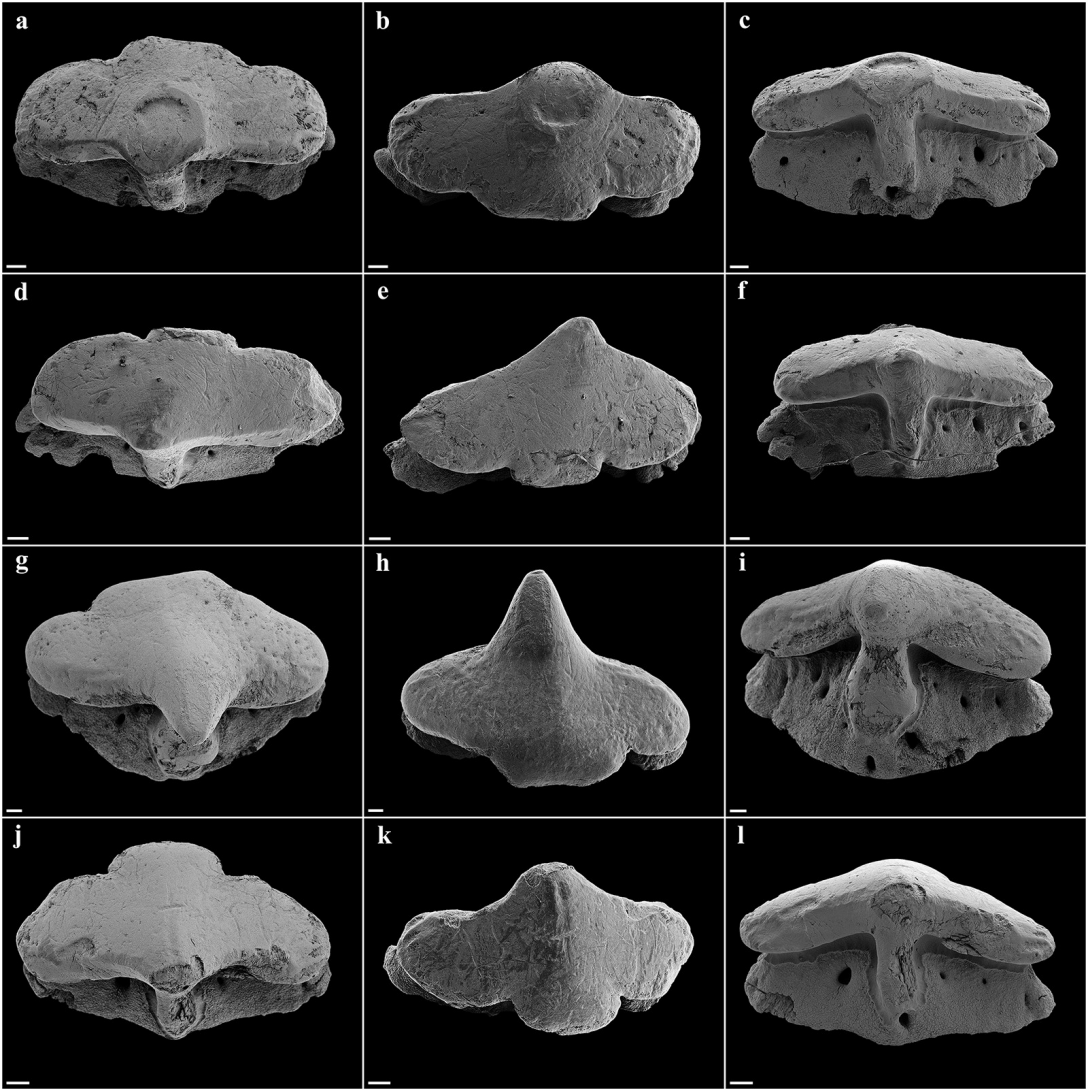
*Pliotrema annae* sp. nov., paratype, ZMH 26362, presumably adult female, 950 mm TL, SEM images of oral teeth. **a**–**c** upper anterolateral tooth of 4th file in **a** occlusal, **b** oblique-lateral, and **c** lingual views; **d**–**f** upper posterolateral tooth of 10th file in **d** occlusal, **e** oblique-lateral, and **f** lingual views; **g**–**i** lower anterolateral tooth of 1st file in **g** occlusal, **h** oblique-lateral, and **i** lingual views; **j**–**l** lower posterolateral tooth from 10th file in **j** occlusal, **k** oblique-lateral, and **l** lingual views. Scale bars: 100 μm.

Eyes lateral on head, moderately large, oval, length 2.8 (2.7)% TL; skeletal interorbital space 0.9 (0.9) times eye length, 9.0 (8.7) times in horizontal preorbital length; posterior eye notches and suborbital grooves present. Spiracles moderately large, length 1.5 (1.3)% TL and 0.5 (0.5) times eye length, left spiracle with 10 (11) folds, right one with 10 (11); spiracles strongly crescentic, oblique, directed posteroventrally from top to bottom, located just posterior to posterior eye notch, separated by a narrow but deep vertical groove along posterior margin of orbit, shorter than eye; upper edge below level of top of eye. Gill slits small, upright, weakly pleated, lateral on head, close to ventral surface, extending slightly onto ventral surface, subequal in length, sixth slit arches around pectoral-fin origin. Mouth moderately large, strongly inferior, broadly arched, symphysis about level with posterior edge of eye, width 4.1 (4.3)% TL and 1.6 (1.6) times in head width; upper labial furrows absent, lower furrows very short, 0.3 (0.3)% TL; corner of mouth partly concealed by lateral muscles of jaw (Fig 19). Teeth unicuspidate, in well-defined series, bases oval and flattened with short but pronounced, narrow median cusp near middle of jaw, no lateral cusps; cusps diminishing in height towards jaw angles, indistinct near jaw corners; about 4–5 series of functional teeth (Fig 20). Median cusp with labial face slightly convex and with both mesial and distal cutting edges weakly bent mesially and distally in occlusal view, respectively. The mesial and distal crown base parts somewhat curve apically. A pronounced and broad, irregularly shaped apron overlaps the junction of crown and root, building a notch at the junction with both mesial and distal crown base parts. Basal ornamentation, striae, reticulations and folds absent in both upper and lower jaw teeth. The lingual face of the cusp is strongly convex, a well-developed uvula is present at the central crown base. The mesial/distal latero-lingual crown faces curve strongly towards the apex of the crown, forming a sharp notch with the uvula. The root is anaulacorhizid and slightly arched without lobation. The outer surface of the root shows large basal foramina, which are mostly oval-shaped. The inner face of the root shows well-developed foramina along the crown-root junction at each side of the uvula. The basal face of the root is flat, partly showing some outer foramina.

Nostrils small, widely separated, subcircular; nostril width 0.7 (0.7)% TL, 4.7 (4.5) times in internarial width, 6.2 (6.5) times in mouth width, 7.7 (7.7) times in width of rostrum at nostrils; located distinctly forward of level of anterior margin of eye; distance from anterior nostrils to symphysis of upper jaw 1.3 (1.3) times internarial space, distance from barbel origin to anterior nostrils 7.9 (8.3)% TL. Anterior nasal flaps well developed, leaf-like, extended ventrally beyond nostrils; incurrent and excurrent apertures surrounded by pronounced marginal lobes; no nasoral or circumnarial grooves; no dermal lobes (Fig 19).

**Fig 21:**
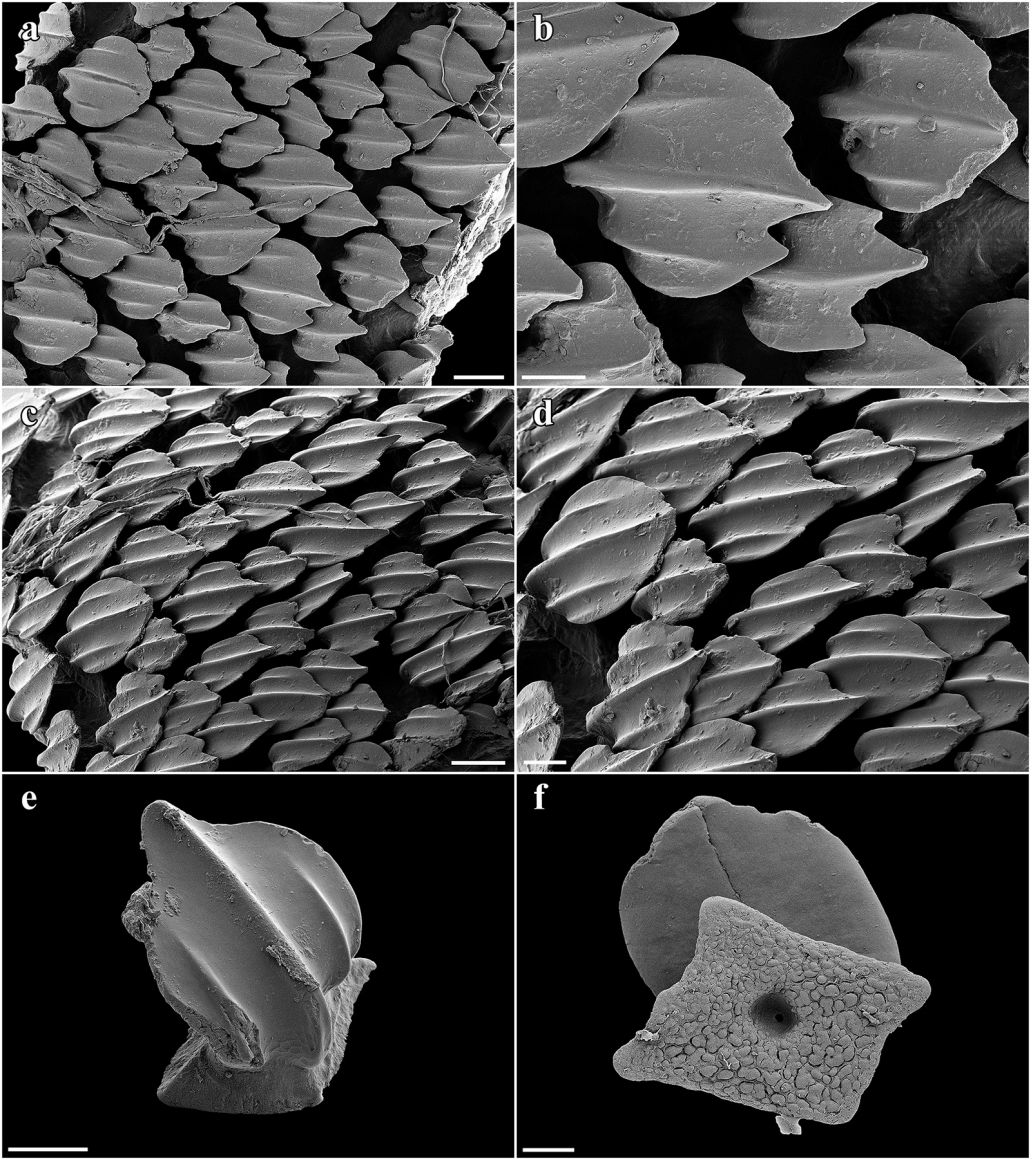
*Pliotrema annae* sp. nov., paratype, ZMH 26362, presumably adult female, 950 mm TL, SEM images of lateral trunk dermal denticles. **a**,**b** dermal denticles in apical views; **c**,**d** dermal denticles in apico-lateral views (image reversed); **e**,**f** single dermal denticles in **e** apico-lateral and **f** basal views. Scale bars: **a**,**c** 200 μm, **b**,**d**,**e** 100 μm, **f** 50 μm.

Lateral trunk dermal denticles densely set and overlapping, with flat, tricuspidate crowns (Fig 21). The lateral cusps are rather weakly pronounced but situated quite far anteriorly so that the median cusp is not much longer than the lateral cusps. The median ridge is strongly pronounced and reaches the tip of the median cusp. The lateral ridges are less pronounced and do not reach the tips of the lateral cusps. The surface of the denticles is only weakly structured by reticulations very close to base. Dermal denticles on rostrum fan-shaped, with an obtusely angled, weakly pronounced median cusp and no lateral cusps but with 6–7 strongly pronounced ridges (Fig 18d–18f). The surface of the rostral dermal denticles is only weakly structured by reticulations very close to base.

**Fig 22:**
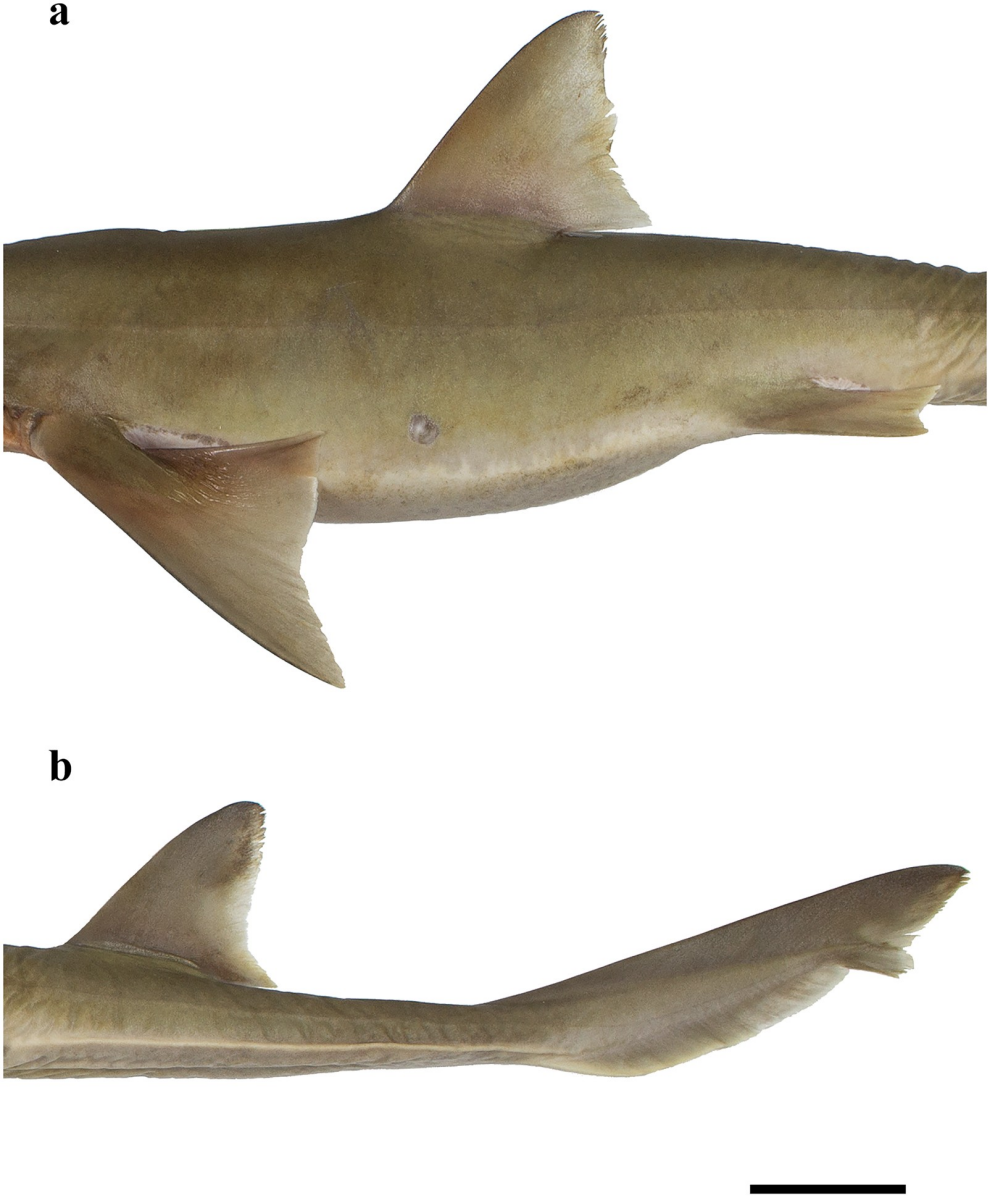
*Pliotrema annae* sp. nov., holotype, ZMH 26361, presumably adult female, 981 mm TL, in fresh (defrozen) condition, lateral views of fins. **a** pectoral, first dorsal, and pelvic fins, **b** second dorsal and caudal fins. Scale bar: 5 cm. Note ventral precaudal ridge in **b**.

Pectoral fins very large, anterior margin weakly convex, 13.4 (12.7)% TL and 1.9 (1.9) times inner margin; apex narrowly rounded; posterior margin weakly concave, directed across horizontal axis at about origin of first dorsal fin; inner margin convex and strongly notched basally; free rear tip angular (Figs 17 and 22a). Pelvic fins large, anterior margin almost straight to slightly convex, 7.1 (7.0)% TL, 1.6 (1.6) times in first dorsal-fin anterior margin, and 1.4 (1.4) times in second dorsal-fin anterior margin; apex narrowly rounded; posterior margin concave; inner margin weakly convex and slightly notched basally; free rear tip broadly rounded; origin distinctly posterior to level free tip of first dorsal fin and well forward of level second dorsal fin origin (Fig 22a).

First dorsal fin broad, semifalcate, anterior margin slightly convex; apex narrowly rounded; posterior margin slanting posteroventrally, slightly convex distally, strongly concave in basal three quarters; inner margin straight, free rear tip narrowly pointed; origin about opposite pectoral-fin free rear tips; insertion and free rear tip clearly anterior to level pelvic-fin origins (Fig 22a). Second dorsal fin somewhat smaller than first but of similar shape, anterior margin weakly convex, apex very narrowly rounded; posterior margin weakly convex distally, strongly concave near basal three quarters; inner margin straight, free rear tip narrowly pointed; origin clearly behind level pelvic insertions; interdorsal space 1.5 (1.4) times first dorsal-fin length, 1.8 (1.6) times dorsal–caudal space; second dorsal-fin inner margin 1.1 (1.1) times subterminal caudal-fin margin (Fig 22b).

Caudal fin short, dorsal margin slightly convex, length 19.1 (19.8)% TL, 1.2 (1.1) times in pelvic–caudal space and 4.6 (5.0) times terminal caudal margin; lower post-ventral lobe absent, upper post-ventral margin slightly convex; terminal lobe well developed, caudal terminal margin slightly concave, apices angular (Fig 22b). Ventral origin of caudal fin situated anteriorly due to low anterior fin ridge (Fig 22b).

Cranium: four anterior-most basiventral cartilages laterally expanded, with curved, dorsally reflected margins. Chondrocranium and cranial nerves highly modified to accomodate the elongated rostrum. Foramen magnum surrounded by crescent-shaped occipital condyles. Dorsal fenestra of the precerebral fossa spindle-shaped, elongate and long, notched anteriorly and posteriorly (Fig 12b).

Skeletal meristics (from radiographs): monospondylous trunk vertebral centra: 53 (54); diplospondylous precaudal centra: 49 (46); total precaudal centra: 102 (100); caudal centra: 52 (54); total centra: 154 (154).

**Fig 23:**
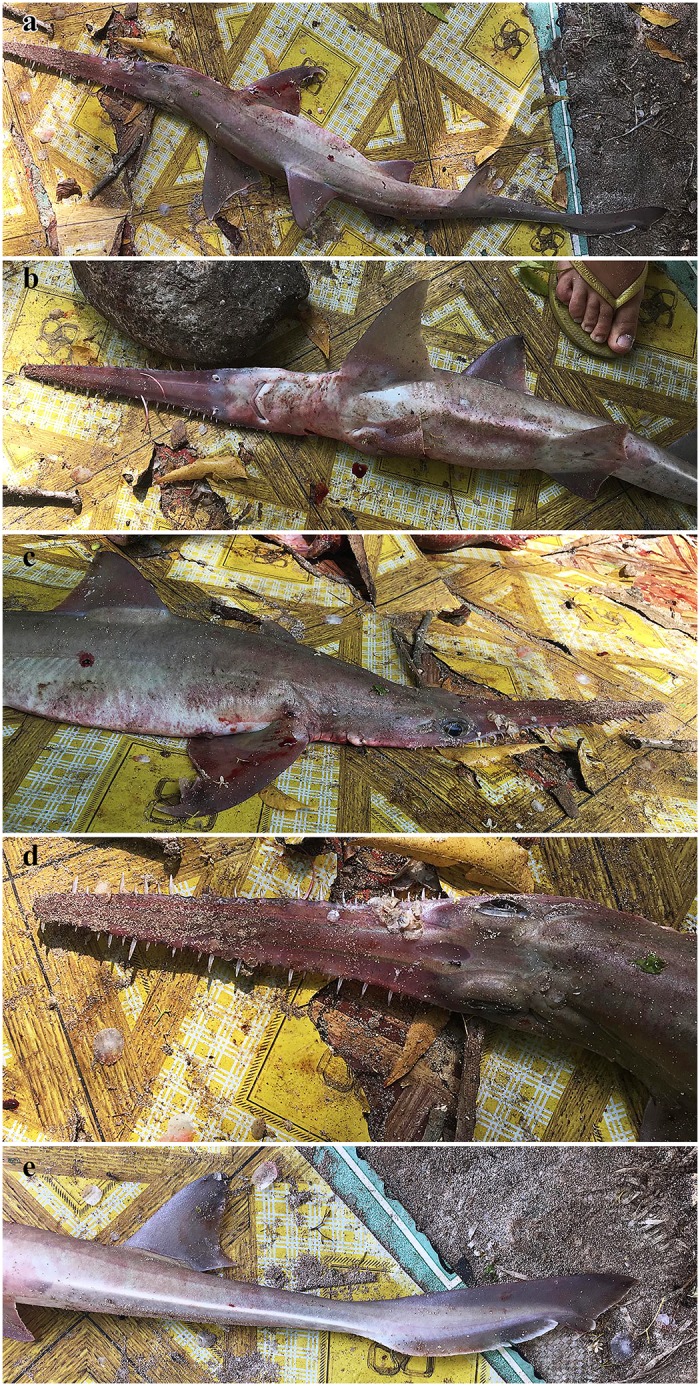
*Pliotrema annae* sp. nov., holotype, ZMH 26361, presumably adult female, 981 mm TL, in fresh condition, images taken directly after landing.

**Fig 24:**
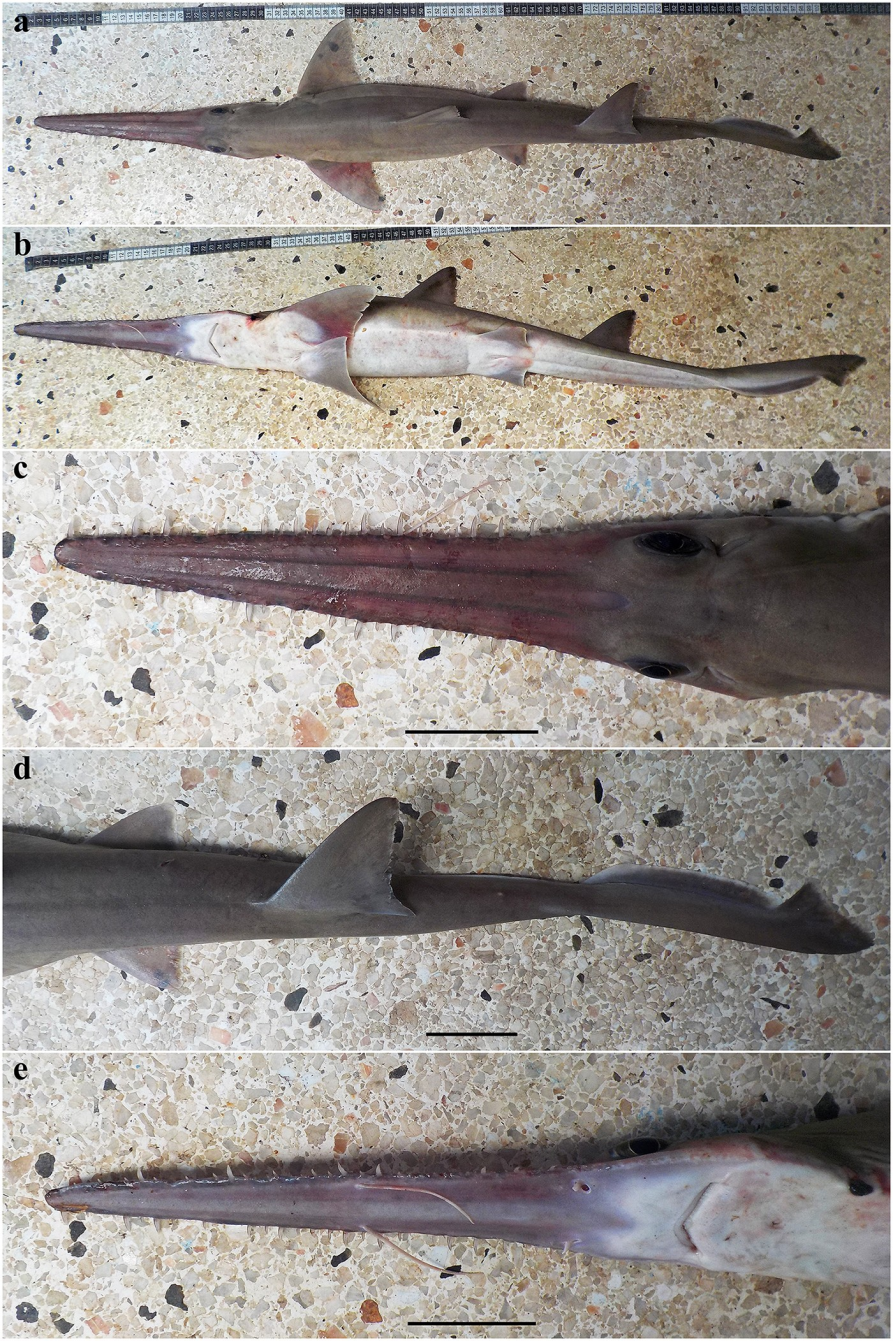
*Pliotrema annae* sp. nov., paratype, ZMH 26362, presumably adult female, 980 mm TL fresh, in fresh condition, images taken directly after landing. Scale bars: 5 cm.

**Fig 25:**
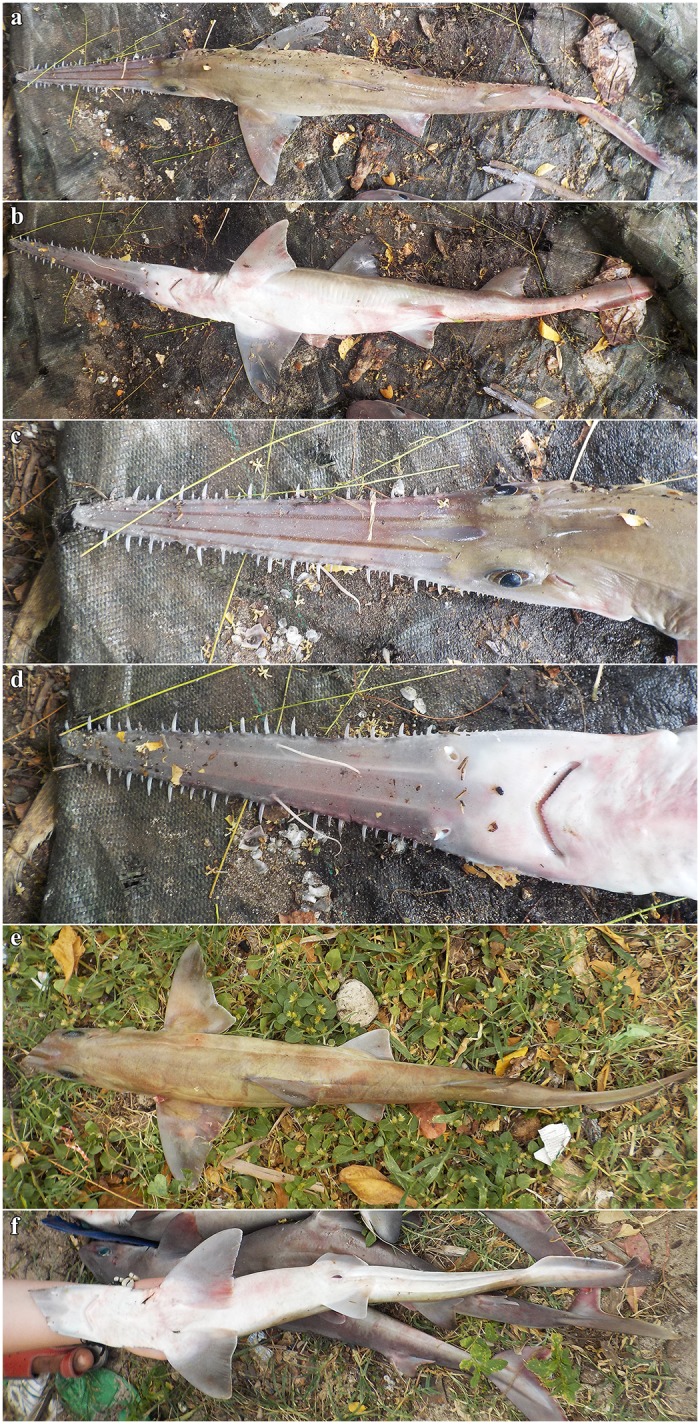
*Pliotrema annae* sp. nov., unretained specimens in fresh condition, images taken directly after catching. **a–d** gravid female, ~980 mm TL, **e**,**f** female with saw cut off, ~580 mm to beginning of saw.

Coloration. Fresh, prior to preservation (types and unretained specimens, Figs 23, 24 and 25): color uniform medium to dark brown dorsally without longitudinal stripes, white ventrally but with few indistinct dark blotches on belly; fins translucent dusky but with white posterior fin margins, particularly pronounced at the posterior pectoral-fin margin and the upper post-ventral and terminal caudal-fin margins; rostrum translucent dusky, dark edged and with two distinct longitudinal stripes dorsally; lateral rostral teeth dark-edged; ventrolateral keels white. Color in preservative (type specimens, Fig 16): coloration similar to fresh coloration, ventral ground coloration yellowish instead of white as usual but dark blotches still present, ventrolateral keels also yellowish; dark edging of rostrum and lateral rostral teeth, as well as longitudinal dorsal rostral stripes still conspicuous.

#### Size

A medium-sized sawshark species reaching about 981 mm TL. As one specimen of ~980 mm TL (not retained) contained six eggs, the holotype and paratype are presumably also adult.

#### Distribution

Known only from off Zanzibar in depths of 20 to 35 m (Fig 14). All four known specimens of this new species were caught in these depths during hours of the darkness / twilight. As both other species of *Pliotrema* usually occur in much deeper waters, *P*. *annae* sp. nov. possibly also occurs in deeper waters during the day but enters shallow water during the night. The area in which the specimens were caught is directly adjacent to a drop-off along the southern tip of Unguja Island. The water depth descends rapidly from ~20 m to >200 m. Accordingly, deep-water sharks such as sixgill sharks and spurdogs are caught, alongside oceanic species such as mako and silky sharks and coastal species such as tiger and bull sharks, smoothhounds, and reef sharks all in the same fishery.

*Pliotrema annae* sp. nov. possibly also occurs off Kenya and/or Somalia following the short description of *P*. *warreni* in Gubanov [28]. *Pliotrema annae* sp. nov. is apparently the only species of the genus occurring in this area.

#### Etymology

The new species is named after Anna Weigmann Huerta, the niece of the first author, to express its relationship to *Pliotrema kajae*, named after the first author’s daughter Kaja Magdalena Weigmann.

#### *Pliotrema warreni* Regan

Proposed English vernacular name: Warren’s sixgill sawshark.

Proposed German vernacular name: Warrens Sechskiemer-Sägehai.

**Fig 26:**
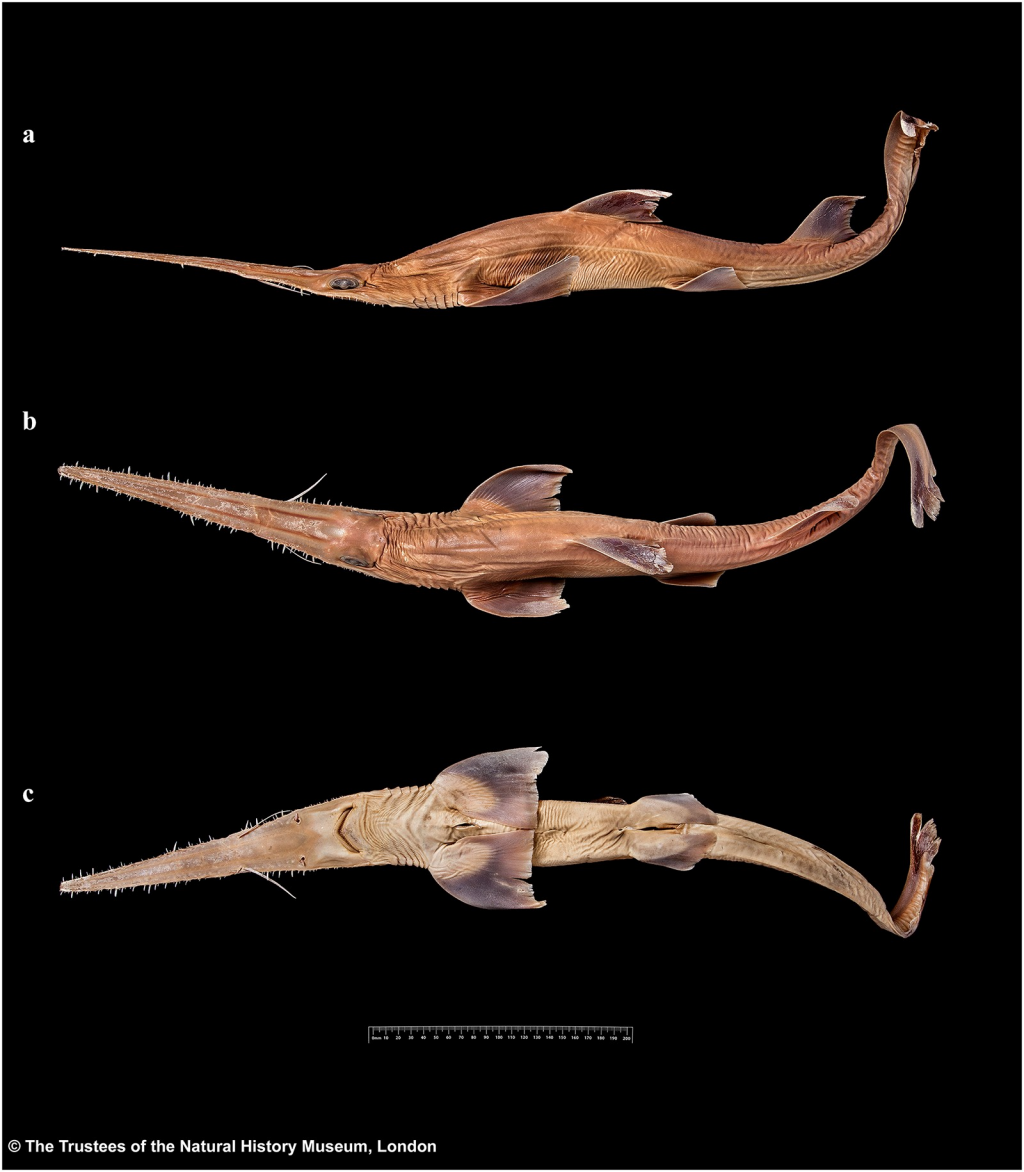
*Pliotrema warreni*, syntype, BMNH 1905.6.8.9, female, 805 mm TL, preserved. **a** lateral, **b** dorsal, and **c** ventral views. The photographs were taken and kindly provided by Kevin Webb.

**Fig 27:**
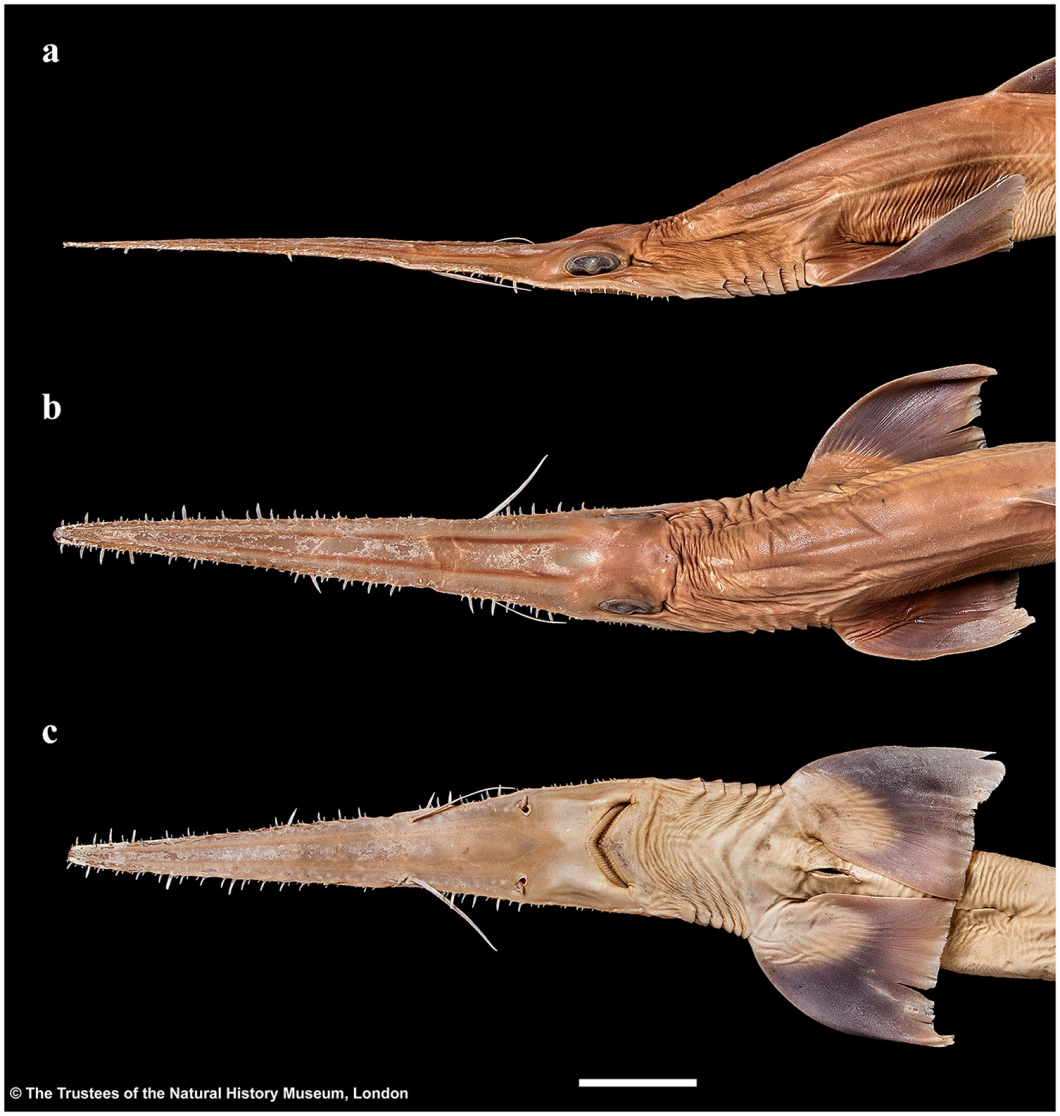
*Pliotrema warreni*, syntype, BMNH 1905.6.8.9, female, 805 mm TL, preserved. Head in **a** lateral, **b** dorsal, and **c** ventral views. Scale bar: 5 cm. The photographs were taken and kindly provided by Kevin Webb.

**Fig 28:**
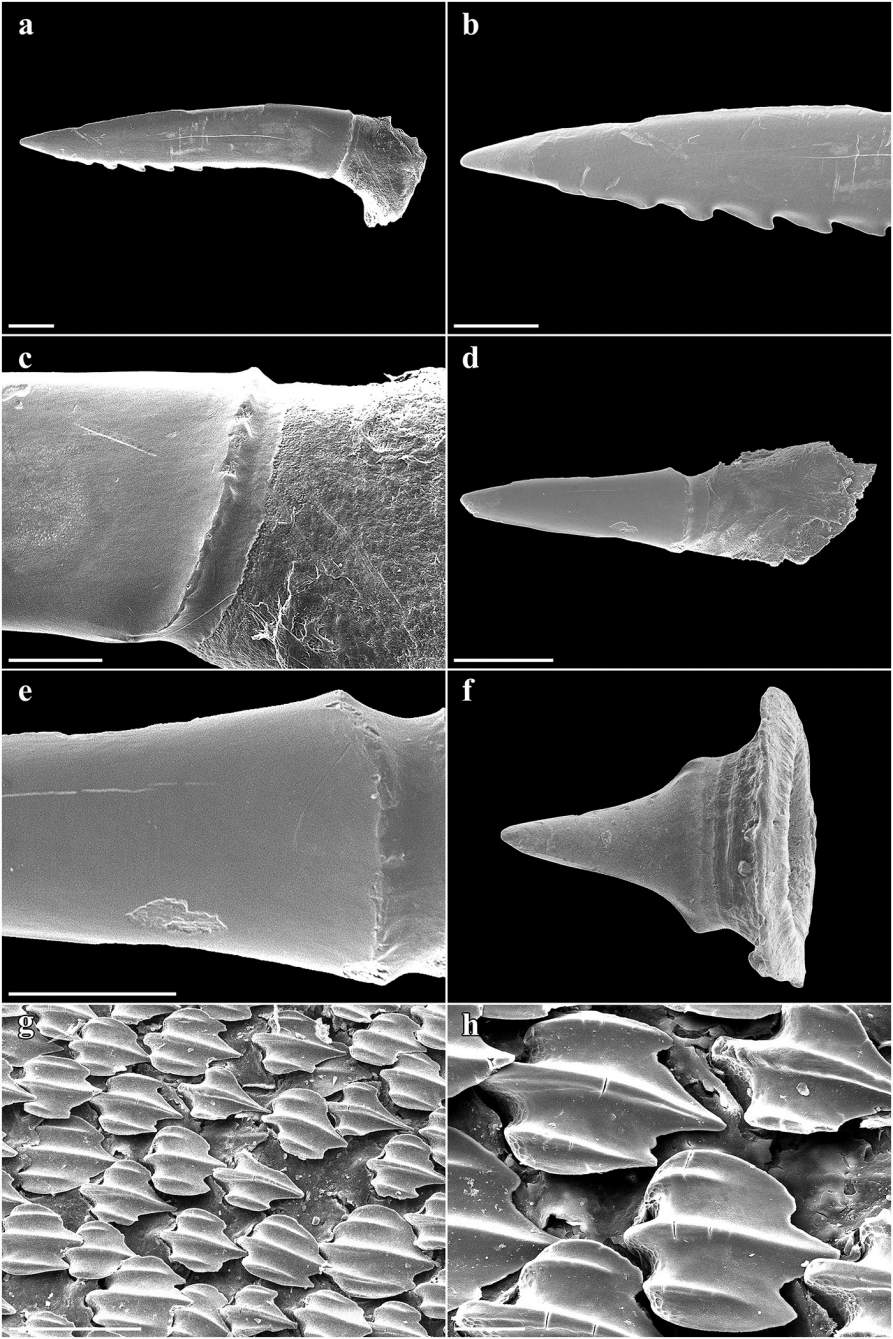
*Pliotrema warreni*, SEM images of rostral teeth and lateral trunk dermal denticles. **a–e**,**g**,**h** SAIAB 208021, female, 925 mm TL; **f** RBINS uncatalogued, adult female, 1300 mm TL. **a–c** large lateral rostral tooth in **a** total and **b**,**c** close-up views; **d**,**e** small interstitial lateral rostral teeth in **d** total and **e** close-up views; **f** ventral rostral spine in total view; **g**,**h** lateral trunk dermal denticles in apical views. Scale bars: **a**,**b**,**d** 1 mm, **c**,**e**,**g** 500 μm, **h** 100 μm. Image 28f was kindly provided by Frederik Mollen, Elasmobranch Research Belgium, from Herman & Ladeuze [29].

**Fig 29:**
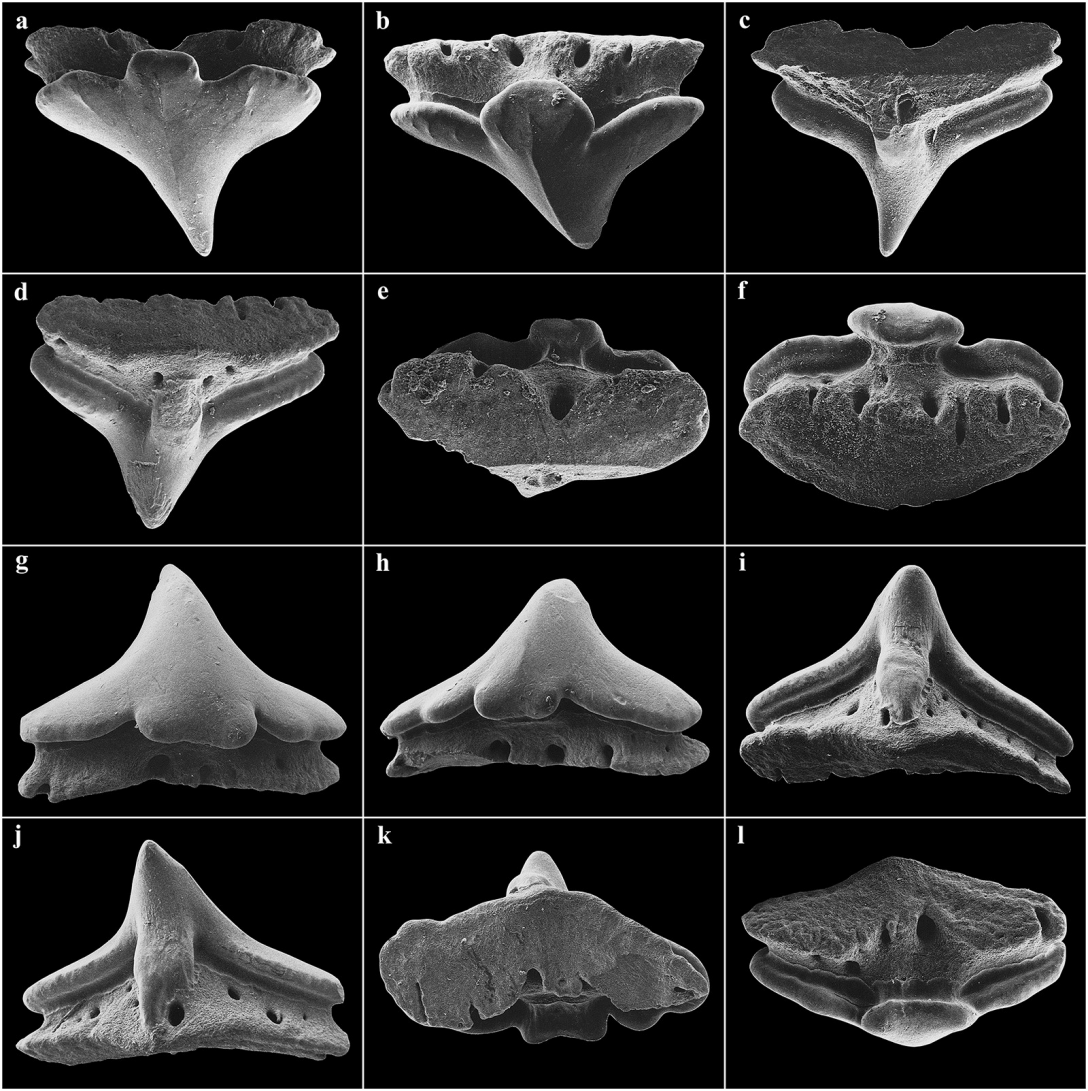
*Pliotrema warreni*, RBINS uncatalogued, adult female, 1300 mm TL, SEM images of oral teeth. **a**–**f** upper anterolateral teeth in **a**,**b** labial, **c**,**d** lingual, and **e**,**f** basal views; **g**–**l** lower anterolateral teeth in **g**,**h** labial, **i**,**j** lingual, and **k**,**l** basal views. The images were kindly provided by Frederik Mollen, Elasmobranch Research Belgium, from Herman & Ladeuze [29].

**Fig 30:**
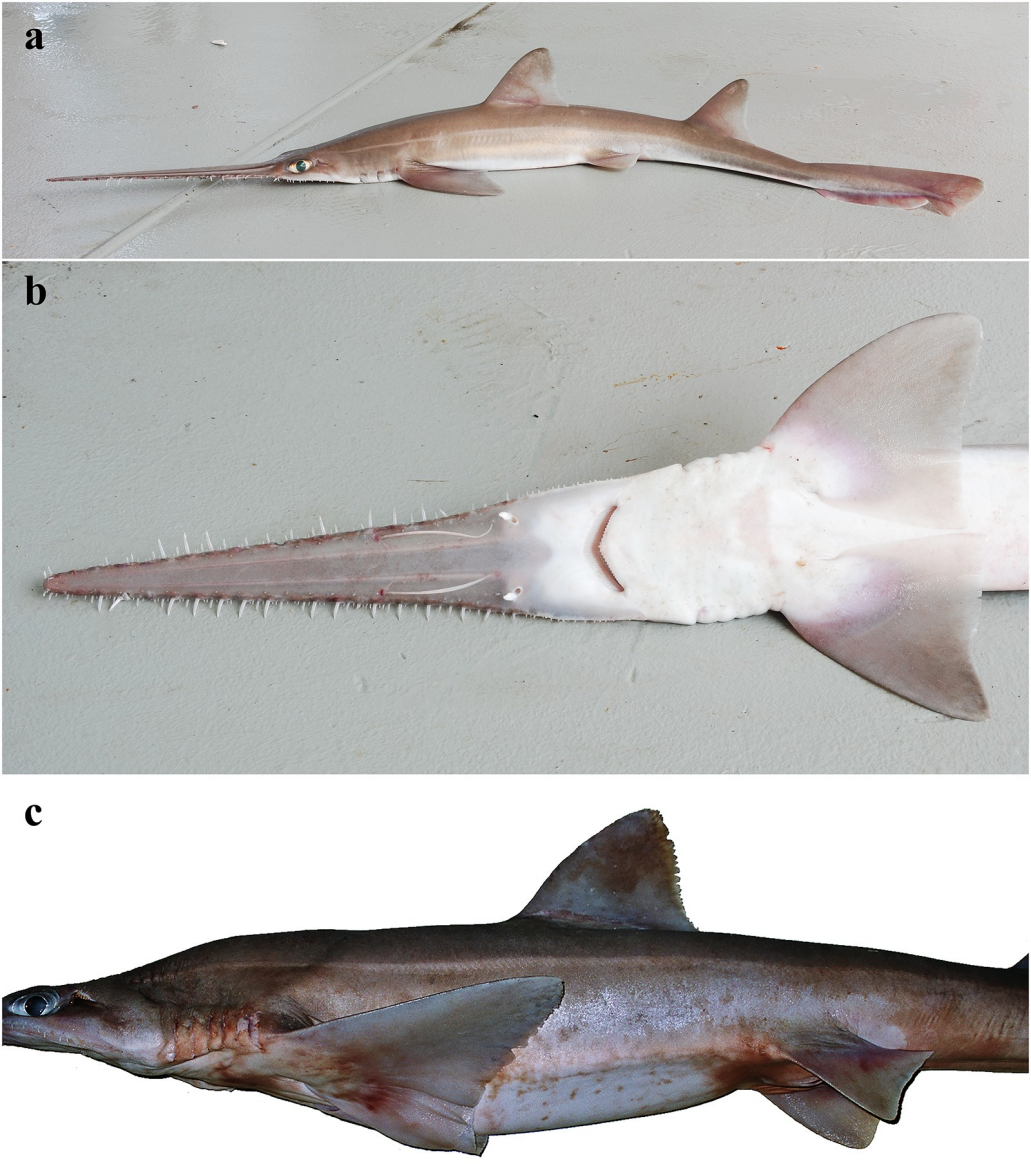
*Pliotrema warreni*, images of specimens in fresh condition. **a**,**b** uncatalogued female, 920 mm TL fresh, in **a** total dorsal and **b** ventral head views, **c** ERB 1105, adult female, 1310 mm TL. Note yellowish longitudinal stripe in **a,c**. The photographs were taken and kindly provided by Oddgeir Berg Alvheim, Institute of Marine Research, Bergen, Norway (a,b) and Frederik Mollen, Elasmobranch Research Belgium (c).

Figs 26–30; Table 4.

*Material examined* (18 specimens). Syntype (**BMNH 1905.6.8.9**): female, 805 mm TL, off the coast of Natal (kwaZulu–Natal), 73 m depth, received from Dr. E. Warren of the Natal Government Museum. Syntype (**BMNH 1899.2.10.4**): heavily dissected female (skeletal parts and remains of flesh and fins only), ~704 mm TL, False Bay, Cape of Good Hope, received from Dr. J.D.F. Gilchrist. **DAFF01**: adult male, 1022 mm TL, FRS ‘Africana’ Cruise AND00003, Station D00331 (off South Africa: 34.29°S 25.97°E), collected on 15 May 2014, trawl 081, grid ID S3659, depth 108 m. **DAFF02**: presumably adult female, 1176 mm TL, FRS ‘Africana’ Cruise CCH00009, Station D00743 (off South Africa: 35.59°S 20.82°E), collected on 01 May 2016, trawl 014, grid ID S2058, depth 94 m. **DAFF03**: gravid female, 1277 mm TL, FRS ‘Africana’ Cruise CCH00009, Station D00802 (off South Africa: 33.90°S 26.45°E), collected on 17 May 2016, trawl 071, grid ID S2326, depth 89 m. **DMM I-E/4946**: female, 785 mm TL, RV ‘Ernst Haeckel’ Cruise 51, off Mozambique, June to September 1980. **ERB 1105**: adult female, 1310 mm TL, FRS ‘Africana’ Cruise Afr-200, Station A23549 (off South Africa: 35°30’S 20°20’E), collected on 23 September 2004, trawl no. 018, grid no. 3069, trawl duration 30 min, 137 m depth (photographs only). **ERB 1106**: subadult male, 945 mm TL, Prawn Trawler, Tugela Bank, 29°14’S 31°31’E, 27 May to 02 June 2006, 10–25 m depth (photographs only). **RBINS uncatalogued**: adult female, 1300 mm TL, off South Africa, Zululand (SEM images only). **SAIAB 186452**: juvenile male, 456.4 mm TL, off KwaZulu-Natal, South Africa, 29°10’49.5”S 32°06’24.6”E, 18 August 2010. **SAIAB 189132**: juvenile female, 405.9 mm TL, off KwaZulu-Natal (Tugela Bank), South Africa, 29°07’30”S 31°45’E, 15 August 2009. **SAIAB 208021**: female, 925 mm TL, Great fish River mouth, 33°29’43"S 27°08’06"E, Eastern Cape, South Africa, found stranded on the beach, Warren Potts, 09 June 2019. **SAM 33313**: 1 specimen, taken off Mozambique, 26°19’0.12”S 33°08’60”E, trawl, 366 m depth, 09 June 1994 (photographs only). **SAM 37244**: two specimens, taken off Mozambique, 22°36’28.8”S 35°42’46.08”E, bottom trawl, 264 m depth, 16 October 2007 (photographs only). **Uncatalogued**: female, 920 mm TL fresh, RV ‘Dr. Fridtjof Nansen’, Survey 2007409, Station 61, off Mozambique, 22°36.48’S 35°42.77’E, 261–264 m depth, bottom trawl # 18, duration 28.4 minutes, 16 Oct 2007 (taken together with three further specimens) (photographs only). **USNM 199741**: adult female, 1350 mm TL, RV ‘Anton Bruun’, Cruise 8, Station 396B, International Indian Ocean Expedition Seychelle Islands Program, 1964, off Delagoa Bay, Mozambique, 25.517°S 33.442°E, 450–455 m depth, 40-ft shrimp trawl, 28 Sep 1964, collector L.W. Knapp (radiographs only). **USNM 353830**: one specimen, RV ‘Africana’, Cruise 106, Station A13997, off South Africa, 33.92°S 26.68°E, 101 m depth, otter trawl, 20 Sep 1992, collectors L.W. Knapp and P.C. Heemstra (radiographs only).

#### Diagnosis

A large six-gilled sawshark with the following characters: barbel origin to anterior nostrils 1.4–1.6 times anterior nostrils to symphysis upper jaw; prenarial length 1.3–1.4 times prebarbel length; preoral length 1.8–2.3 times interdorsal space; pectoral-fin anterior margin 1.4–1.5 times dorsal–caudal space; mouth width 3.1–3.9 times spiracle length. First dorsal fin originates about opposite pectoral-fin free rear tips. Lateral trunk dermal denticles tricuspidate, rather flat and imbricated. Color medium to dark brown dorsally with a pronounced yellowish longitudinal stripe; uniform white ventrally; dorsal rostrum surface with two distinct longitudinal dark stripes, lateral rostral teeth dark-edged. Monospondylous centra 53–56; precaudal diplospondylous centra 49–51; total vertebral centra 154–158. *Pliotrema warreni* clearly differs from both new species in a rostrum that is not constricted between barbel origin and nostrils and barbels that are situated about two thirds way from rostral tip to mouth, with prebarbel length about twice, i.e. 1.7–2.1 times, distance from barbel origin to symphysis of upper jaw (vs. barbels situated about half way from rostral tip to mouth, with prebarbel length about equal, i.e. 1.0–1.1 times, distance from barbel origin to symphysis of upper jaw), prebarbel length 60.2–68.0% vs. 49.4–52.9% of preoral length, preoral length 1.5–1.7 vs. 1.9–2.0 times prebarbel length, and prenarial length 1.3–1.4 vs. 1.5–1.7 times prebarbel length.

#### Description

The description is based on the intact syntype BMNH 1905.6.8.9, as well as the four specimens DMM I-E/4946, SAIAB 186452, SAIAB 189132, and SAIAB 208021. Where relevant, ratios are based on horizontal measurements unless otherwise stated. Morphometric measurements and meristics are given in Table 4.

**Table 4:** *Pliotrema warreni*, morphometrics and meristics of the intact syntype BMNH 1905.6.8.9 and the four specimens DMM I-E/4946, SAIAB 186452, SAIAB 189132, and SAIAB 208021, as well as ranges and means for the five specimens. Proportional values are expressed as percentages of total length (TL) 70% ethanol preserved except for minimum, maximum, and mean of TL in mm.

	*Pliotrema warreni*, intact female syntype, BMNH 1905.6.8.9		*Pliotrema warreni*, female, DMM I-E/4946		*Pliotrema warreni*, juvenile male, SAIAB 186452		*Pliotrema warreni*, juvenile female, SAIAB 189132		*Pliotrema warreni*, female, SAIAB 208021		Minimum (n = 5)	Maximum (n = 5)	Mean (n = 5)
	mm	% TL	mm	% TL	mm	% TL	mm	% TL	mm	% TL	% TL	% TL	% TL
TL, total length	805.0	100.0	785.0	100.0	456.4	100.0	405.9	100.0	925.0	100.0	405.9	925.0	675.5
PRC, precaudal length, dorsally	660.0	82.0	635.0	80.9	373.4	81.8	323.5	79.7	750.0	81.1	79.7	82.0	81.1
PRVC, precaudal length, ventrally	670.0	83.2	643.0	81.9	360.4	79.0	327.3	80.6	745.0	80.5	79.0	83.2	81.1
PD2, pre-D2-length	552.0	68.6	530.0	67.5	310.9	68.1	270.2	66.6	620.0	67.0	66.6	68.6	67.6
PD1, pre-D1-length	378.0	47.0	370.0	47.1	217.8	47.7	198.7	49.0	420.0	45.4	45.4	49.0	47.2
HDL, head length (to end of last gill slit), horizontally	304.0	37.8	285.0	36.3	178.8	39.2	162.6	40.1	328.0	35.5	35.5	40.1	37.8
HDL, head length (to end of last gill slit), point to point	309.0	38.4	287.5	36.6	177.6	38.9	163.1	40.2	333.0	36.0	36.0	40.2	38.0
PG1, prebranchial length, horizontally	277.0	34.4	264.0	33.6	163.3	35.8	144.6	35.6	297.0	32.1	32.1	35.8	34.3
PG1, prebranchial length, point to point	280.0	34.8	266.0	33.9	163.7	35.9	147.2	36.3	299.0	32.3	32.3	36.3	34.6
PSP, prespiracular length, horizontally	242.0	30.1	226.0	28.8	140.3	30.7	125.3	30.9	252.0	27.2	27.2	30.9	29.5
PSP, prespiracular length, point to point	245.0	30.4	228.5	29.1	140.8	30.9	128.0	31.5	253.0	27.4	27.4	31.5	29.9
POB, preorbital length, horizontally	211.0	26.2	196.0	25.0	122.6	26.9	107.1	26.4	223.0	24.1	24.1	26.9	25.7
POB, preorbital length, point to point	216.0	26.8	200.0	25.5	123.5	27.1	109.8	27.1	235.0	25.4	25.4	27.1	26.4
PP1, prepectoral length, horizontally	300.0	37.3	285.0	36.3	177.8	39.0	158.7	39.1	327.0	35.4	35.4	39.1	37.4
PP2, prepelvic length, horizontally	458.0	56.9	450.0	57.3	265.0	58.1	228.0	56.2	521.0	56.3	56.2	58.1	57.0
SVL, snout–anterior vent length	475.0	59.0	466.0	59.4	273.8	60.0	236.0	58.1	540.0	58.4	58.1	60.0	59.0
IDS, interdorsal space	118.0	14.7	113.1	14.4	66.0	14.5	53.6	13.2	135.0	14.6	13.2	14.7	14.3
DCS, dorsal (D2)–caudal space	70.0	8.7	63.6	8.1	34.2	7.5	32.9	8.1	77.0	8.3	7.5	8.7	8.1
PPS, pectoral–pelvic space	171.0	21.2	151.7	19.3	81.3	17.8	57.2	14.1	174.0	18.8	14.1	21.2	18.3
PCA, pelvic–caudal space	177.0	22.0	157.8	20.1	87.0	19.1	83.8	20.6	190.0	20.5	19.1	22.0	20.5
VCL, anterior vent–caudal tip length	330.0	41.0	320.0	40.8	184.5	40.4	166.2	40.9	386.0	41.7	40.4	41.7	41.0
PRN, prenarial length, horizontally	205.0	25.5	189.5	24.1	116.6	25.5	103.3	25.4	209.0	22.6	22.6	25.5	24.6
POR, preoral length	237.0	29.4	220.5	28.1	137.8	30.2	121.3	29.9	247.0	26.7	26.7	30.2	28.9
EYL, eye length	24.0	3.0	24.3	3.1	16.6	3.6	16.1	4.0	26.6	2.9	2.9	4.0	3.3
EYH, eye height	10.0	1.2	9.7	1.2	7.0	1.5	6.6	1.6	13.6	1.5	1.2	1.6	1.4
ING, intergill length 1st to last slit	23.0	2.9	24.8	3.2	16.5	3.6	14.7	3.6	28.4	3.1	2.9	3.6	3.3
GS1, 1st gill slit height (unspread)	9.0	1.1	10.7	1.4	6.4	1.4	5.7	1.4	11.6	1.3	1.1	1.4	1.3
GS2, 2nd gill slit height	10.0	1.2	11.0	1.4	6.3	1.4	5.6	1.4	12.2	1.3	1.2	1.4	1.3
GS3, 3rd gill slit height	10.0	1.2	11.3	1.4	6.6	1.4	5.0	1.2	11.7	1.3	1.2	1.4	1.3
GS4, 4th gill slit height	10.0	1.2	11.9	1.5	5.8	1.3	4.6	1.1	12.1	1.3	1.1	1.5	1.3
GS5, 5th gill slit height	10.0	1.2	11.4	1.5	6.1	1.3	4.9	1.2	13.8	1.5	1.2	1.5	1.3
GS6, 6th gill slit height	10.0	1.2	10.4	1.3	5.8	1.3	4.6	1.1	13.3	1.4	1.1	1.4	1.3
P1A, pectoral anterior margin length	96.0	11.9	94.6	12.1	48.9	10.7	47.1	11.6	112.6	12.2	10.7	12.2	11.7
P1B, pectoral base length	25.0	3.1	25.7	3.3	13.8	3.0	12.8	3.2	30.8	3.3	3.0	3.3	3.2
P1I, pectoral inner margin length	62.0	7.7	60.4	7.7	37.8	8.3	32.1	7.9	77.0	8.3	7.7	8.3	8.0
P1P, pectoral posterior margin length	75.0	9.3	81.6	10.4	40.9	9.0	38.6	9.5	103.5	11.2	9.0	11.2	9.9
P1H, pectoral height, base end to tip	91.0	11.3	88.7	11.3	52.5	11.5	46.4	11.4	118.1	12.8	11.3	12.8	11.7
P1L, P length anterior base to posterior tip	83.0	10.3	82.4	10.5	47.5	10.4	40.9	10.1	97.3	10.5	10.1	10.5	10.4
CDM, dorsal caudal margin length	145.0	18.0	148.2	18.9	84.7	18.6	76.7	18.9	176.6	19.1	18.0	19.1	18.7
CST, subterminal caudal margin length	23.0	2.9	24.1	3.1	12.8	2.8	13.8	3.4	25.0	2.7	2.7	3.4	3.0
CSW, subterminal caudal width	21.0	2.6	23.7	3.0	12.5	2.7	12.1	3.0	26.8	2.9	2.6	3.0	2.8
CTR, terminal caudal margin length	27.0	3.4	29.4	3.7	17.8	3.9	14.2	3.5	48.1	5.2	3.4	5.2	3.9
CTL, terminal caudal lobe length	43.0	5.3	43.0	5.5	26.9	5.9	24.5	6.0	53.9	5.8	5.3	6.0	5.7
D1L, D1 total length	78.0	9.7	79.9	10.2	41.1	9.0	37.3	9.2	95.1	10.3	9.0	10.3	9.7
D1A, D1 anterior margin length	86.0	10.7	83.9	10.7	46.2	10.1	41.0	10.1	94.4	10.2	10.1	10.7	10.4
D1B, D1 base length	55.0	6.8	55.7	7.1	28.9	6.3	24.4	6.0	62.2	6.7	6.0	7.1	6.6
D1H, D1 vertical height	44.0	5.5	51.7	6.6	35.1	7.7	26.8	6.6	65.4	7.1	5.5	7.7	6.7
D1I, D1 inner margin length	26.0	3.2	26.7	3.4	13.3	2.9	12.2	3.0	29.3	3.2	2.9	3.4	3.1
D1P, D1 posterior margin length	44.0	5.5	49.6	6.3	34.7	7.6	29.6	7.3	64.3	7.0	5.5	7.6	6.7
D2L, D2 total length	71.0	8.8	69.2	8.8	38.1	8.3	22.0	5.4	80.0	8.6	5.4	8.8	8.0
D2A, D2 anterior margin length	77.0	9.6	79.7	10.1	45.3	9.9	36.6	9.0	87.8	9.5	9.0	10.1	9.6
D2B, D2 base length	48.0	6.0	46.8	6.0	26.4	5.8	22.1	5.4	51.8	5.6	5.4	6.0	5.7
D2H, D2 vertical height	42.0	5.2	48.3	6.2	29.4	6.4	25.2	6.2	55.9	6.0	5.2	6.4	6.0
D2I, D2 inner margin length	24.0	3.0	24.1	3.1	12.3	2.7	11.4	2.8	28.1	3.0	2.7	3.1	2.9
D2P, D2 posterior margin length	37.0	4.6	44.8	5.7	25.0	5.5	22.0	5.4	58.4	6.3	4.6	6.3	5.5
P2L, pelvic total length	63.0	7.8	64.8	8.3	36.9	8.1	31.1	7.6	76.4	8.3	7.6	8.3	8.0
P2A, pelvic anterior margin length	47.0	5.8	51.0	6.5	28.4	6.2	25.3	6.2	62.7	6.8	5.8	6.8	6.3
P2B, pelvic base length	34.0	4.2	33.5	4.3	16.8	3.7	16.0	3.9	46.6	5.0	3.7	5.0	4.2
P2H, pelvic height = max. width (excl. clasper)	38.0	4.7	39.7	5.1	17.8	3.9	17.1	4.2	49.2	5.3	3.9	5.3	4.6
P2I, pelvic inner margin length	30.0	3.7	32.4	4.1	18.7	4.1	14.9	3.7	38.0	4.1	3.7	4.1	3.9
P2P, pelvic posterior margin length	43.0	5.3	42.1	5.4	22.8	5.0	19.2	4.7	52.9	5.7	4.7	5.7	5.2
HDH, head height at P origin	41.0	5.1	39.5	5.0	26.0	5.7	22.2	5.5	51.4	5.6	5.0	5.7	5.4
TRH, trunk height at P base end	46.0	5.7	43.0	5.5	26.7	5.8	25.2	6.2	54.8	5.9	5.5	6.2	5.8
ABH, abdomen height at D1 base end	45.0	5.6	40.3	5.1	29.4	6.4	21.6	5.3	55.4	6.0	5.1	6.4	5.7
TAH, tail height at pelvic base end	33.0	4.1	35.1	4.5	19.3	4.2	14.9	3.7	42.8	4.6	3.7	4.6	4.2
CPH, caudal peduncle height at dorsal caudal-fin origin	15.0	1.9	17.6	2.2	9.9	2.2	9.1	2.2	20.2	2.2	1.9	2.2	2.1
DPI, D1 midpoint–pectoral base end	90.0	11.2	86.4	11.0	44.9	9.8	37.8	9.3	103.3	11.2	9.3	11.2	10.5
DPO, D1 midpoint–pelvic origin	52.0	6.5	49.6	6.3	22.9	5.0	22.0	5.4	60.8	6.6	5.0	6.6	6.0
PDI, pelvic midpoint–D1 base end	59.0	7.3	56.8	7.2	28.7	6.3	16.4	4.0	55.4	6.0	4.0	7.3	6.2
PDO, pelvic midpoint–D2 origin	66.0	8.2	63.4	8.1	38.0	8.3	32.7	8.1	70.4	7.6	7.6	8.3	8.1
MOL, mouth length (arc radius)	9.0	1.1	9.2	1.2	8.9	1.9	4.3	1.0	7.7	0.8	0.8	1.9	1.2
MOW, mouth width	34.0	4.2	33.7	4.3	22.0	4.8	20.1	4.9	39.8	4.3	4.2	4.9	4.5
ULA, upper labial furrow length	0.0	0.0	0.0	0.0	0.0	0.0	0.0	0.0	0.0	0.0	0.0	0.0	0.0
LLA, lower labial furrow length	4.0	0.5	4.2	0.5	2.7	0.6	2.6	0.6	5.8	0.6	0.5	0.6	0.6
NOW, nostril width	5.0	0.6	5.6	0.7	5.0	1.1	4.5	1.1	7.2	0.8	0.6	1.1	0.9
INW, internarial width	27.0	3.4	25.0	3.2	17.7	3.9	13.9	3.4	28.1	3.0	3.0	3.9	3.4
ANF, anterior nasal flap length	6.0	0.7	5.6	0.7	3.8	0.8	3.9	0.9	7.1	0.8	0.7	0.9	0.8
INOI, interorbital space, integumental	33.0	4.1	31.7	4.0	23.0	5.0	20.8	5.1	33.1	3.6	3.6	5.1	4.4
INOS, interorbital space, skeletal	18.0	2.2	20.7	2.6	14.0	3.1	12.2	3.0	25.7	2.8	2.2	3.1	2.7
SPL, spiracle length	11.0	1.4	10.1	1.3	5.7	1.2	5.3	1.3	11.8	1.3	1.2	1.4	1.3
ESL, eye–spiracle space	4.0	0.5	4.5	0.6	3.5	0.8	3.3	0.8	4.3	0.5	0.5	0.8	0.6
HDW, head width at middle gill slits	54.0	6.7	49.6	6.3	31.9	7.0	27.5	6.8	65.8	7.1	6.3	7.1	6.8
TRW, trunk width at P base ends	48.0	6.0	55.5	7.1	31.3	6.8	23.9	5.9	62.9	6.8	5.9	7.1	6.5
ABW, abdomen width at D1 base end	43.0	5.3	50.8	6.5	32.4	7.1	22.0	5.4	56.3	6.1	5.3	7.1	6.1
TAW, tail width at pelvic base ends	33.0	4.1	36.5	4.6	20.1	4.4	18.1	4.4	46.9	5.1	4.1	5.1	4.5
CPW, C peduncle width at dorsal caudal-fin origin	10.0	1.2	13.3	1.7	7.6	1.7	7.4	1.8	16.3	1.8	1.2	1.8	1.6
CLO, clasper outer margin length	-	-	-	-	2.0	0.4	-	-	-	-	0.4	0.4	0.4
CLI, clasper inner margin length	-	-	-	-	9.5	2.1	-	-	-	-	2.1	2.1	2.1
CLB, clasper base width	-	-	-	-	3.3	0.7	-	-	-	-	0.7	0.7	0.7
BAL, Barbel length	45.0	5.6	48.1	6.1	39.6	8.7	39.0	9.6	52.9	5.7	5.6	9.6	7.1
PBL, Prebarbel length, horizontally	157.0	19.5	149.9	19.1	92.1	20.2	73.0	18.0	152.9	16.5	16.5	20.2	18.7
BSJ, Barbel origin to symphysis upper jaw	81.0	10.1	72.4	9.2	49.1	10.8	44.2	10.9	91.7	9.9	9.2	10.9	10.2
BAN, Barbel origin to anterior nostrils	50.0	6.2	42.8	5.5	29.2	6.4	26.8	6.6	56.0	6.1	5.5	6.6	6.1
ANJ, Anterior nostrils to symphysis upper jaw	32.0	4.0	30.3	3.9	21.5	4.7	19.8	4.9	35.7	3.9	3.9	4.9	4.3
INS, Interspiracular space	32.0	4.0	32.3	4.1	20.7	4.5	18.5	4.5	37.7	4.1	4.0	4.5	4.2
RWN, Rostral width at anterior nostrils	46.0	5.7	44.9	5.7	29.6	6.5	27.4	6.7	48.7	5.3	5.3	6.7	6.0
RWB, Rostral width at origin of barbels	31.0	3.9	32.7	4.2	22.4	4.9	20.6	5.1	34.3	3.7	3.7	5.1	4.3
RTAL, Rostral tooth length (anterior of nostrils): Length of longest tooth immediately anterior to barbel	5.0	0.6	3.6	0.5	5.2	1.1	5.5	1.3	5.0	0.5	0.5	1.3	0.8
RTAW, Rostral tooth width (anterior of nostrils): Width of exposed base of above tooth	1.0	0.1	0.8	0.1	0.9	0.2	1.2	0.3	2.2	0.2	0.1	0.3	0.2
RTIS, 1° rostral tooth interspace: First complete interspace anterior to barbels	9.0	1.1	6.8	0.9	3.2	0.7	3.9	0.9	8.9	1.0	0.7	1.1	0.9
RTIL, 2° rostral tooth length: Longest complete tooth within above primary interspace	3.0	0.4	1.6	0.2	2.0	0.4	2.5	0.6	2.4	0.3	0.2	0.6	0.4
RTPL, Rostral tooth length (posterior of nostrils): Longest rostral tooth in this region	2.0	0.2	2.6	0.3	2.9	0.6	3.3	0.8	2.2	0.2	0.2	0.8	0.4
spiracle folds left/right	13/13		10/10		11/13		11/11		12/11		10/10	13/13	11.4/11.6
total large lateral rostral teeth l./r.	23/22		23/23		34/34		34/34		21/21		21/21	34/34	27.0/26.8
large lateral rostral teeth anterior to barbels l./r.	17/16		17/18		16/16		15/16		15/14		15/14	17/18	16.0/16.0
large lateral rostral teeth posterior to barbels l./r.	6/6		6/5		18/18		19/18		6/7		6/5	19/18	11.0/10.8
ventral rostral spines anterior to nostrils l./r.	19/19		21/21		22/22		24/24		17/16		17/16	24/24	20.6/20.4
ventral rostral spines anterior to barbel origin l./r.	14/14		16/16		15/15		15/14		11/10		11/10	16/16	14.2/13.8
tooth rows, upper jaw	43		37		37		38		44		37	44	39.8
tooth rows, lower jaw	34		34		33		35		41		33	41	35.4
Vtr, monospondylous trunk vertebrae centra	56		53		55		55		53		53	56	54.4
dipl. VprC, diplospondylous precaudal vertebrae centra	49		50		51		49		50		49	51	49.8
VprC, total precaudal vertebrae centra	105		103		106		104		103		103	106	104.2
VtermC, caudal vertebrae centra	50		55		52		50		54		50	55	52.2
total vertebrae centra	155		158		158		154		157		154	158	156.4

External morphology. Body firm and slender, depressed forward of gills, abdomen subcircular in cross-section, tail subtriangular in cross-section, deepest at abdomen; not tapering gradually and evenly beyond pectoral fins; snout flattened, greatly extended, saw-like; abdomen elongate, horizontal head length 0.6–0.7 times snout–anterior vent length, pectoral–pelvic space 14.1–21.2% TL; pelvic–caudal space 2.4–2.8 times pelvic-fin length; tail flattened ventrally, elongate, snout–anterior vent length 1.4–1.5 times anterior vent–caudal tip length; caudal peduncle short, dorsal–caudal space 7.5–8.7% TL, caudal peduncle height 3.5–4.7 times in dorsal–caudal space and width 1.2–1.5 times in height; ventrolateral keels well developed, extending from slightly behind level of free rear tip of pelvic fins to beyond origin of ventral lobe of caudal fin, converging strongly near their posterior extremity; no precaudal pit; no median predorsal, postdorsal or preventral caudal grooves (Fig 26).

Head narrow, subtriangular and deepest at sixth gill slit, strongly depressed above eyes, head width 6.3–7.1% TL, 1.2–1.3 times head height. Snout forming a very elongate, blade-like rostrum. Rostrum triangular in dorsal view; not constricted between barbel origin and nostrils, sides of rostrum nearly straight from tip to origin of orbit; tip narrowly rounded; rostrum extending laterally below eyes as a well-defined suborbital ridge along ventrolateral edge of head, terminating somewhat behind level of posterior edge of spiracle (Fig 27).

A slender, filamentous, dorsoventrally flattened barbel originating on the ventrolateral margin about two thirds way from rostral tip to mouth on each side, with prebarbel length 1.7–2.1 times distance from barbel origin to symphysis of upper jaw, 60.2–68.0% of preoral length and 16.5–20.2% TL. Barbel length 1.9–3.5 times in prebarbel length and 1.1–1.8 times in length from barbel origin to symphysis of upper jaw. Preorbital length, horizontally 5.3–6.2 times mouth width, 18.9–21.5 times spiracle length, 2.3–3.0 times first dorsal-fin length, 3.9–4.6 times rostral width at anterior nostrils; extremely narrow in lateral view; preoral length 26.7–30.2% TL, 3.8–4.4 times head width, 4.4–5.2 times rostral width at anterior nostrils, 5.9–7.6 times rostral width at origin of barbels, 1.5–1.7 times prebarbel length, 1.2–1.2 times prenarial length, and 1.8–2.3 times interdorsal space (Fig 27).

Large lateral rostral teeth of prenarial portion of rostrum variable in length, curved, rather stout, serrated, longest about half way from apex of rostrum to barbel origin; longest tooth immediately anterior to barbels shorter than spiracle length, length 0.5–1.3% TL and 0.5–1.7 times first complete interspace anterior to barbels, width 0.1–0.3% TL; anteriormost tooth close to tip of rostrum small, followed by the first large tooth; large teeth shortest near nostrils, longest rostral tooth posterior to nostrils 0.2–0.8% TL; large teeth absent behind nostrils but interstitial-like teeth present, short to very short and closely set, partially directed almost ventrally, particularly near mouth. Interspaces between large rostral teeth rather regularly sized, about as long as adjacent teeth, with 2–4 smaller, variable interstitial teeth. Rostral tooth counts mostly symmetrical between left and right hand sides; left side with ~21–~34 large teeth, right side with ~21–~34); anterior to barbels left side with ~15–~17 large rostral teeth, right side with ~14–~18, posterior to barbels left side with ~6–~19 large rostral teeth, right side with ~5–~18; anterior to nostrils left side with ~17–~24 ventral spines, right side with ~16–~27, anterior to barbel origin left side with ~11–~15 ventral spines, right side with ~10–~19; one enlarged ventral spine, distinctly larger than the other ventral spines, present just in front of each nostril. Large rostral teeth (Fig 28a–28c) with elongated crown and oval-shaped base, slightly bent to the rear and flattened towards the apex, forming anterior and posterior cutting edges at front and rear, the latter serrated by barbed hooks. Crown base with numerous short longitudinal ridges forming a pronounced transversal crest. Both, anterior and posterior faces of the root are curved outwards from the junction of crown and root towards the base of the root. The basal face shows a deep v-shaped median groove that is antero-posteriorly directed and has an oval-shaped cavity in the center. Large interstitial rostral teeth similar but with somewhat less pronounced serration in specimens of 704 mm TL (heavily dissected syntype BMNH 1899.2.10.4) or larger. Large interstitial rostral teeth without serration in specimens of 456.4 mm TL (juvenile male SAIAB 186452) or smaller. Small interstitial teeth (Fig 28d and 28e) with blade-shaped crown and without serration in all specimens. Crown of ventral spines (Fig 28f) elongated cone-shaped with a pronounced transversal basal ridge, root with roundish and pedestal-like base. The basal face has a large and deep roundish foramen in the center.

Eyes lateral on head, large, oval, length 2.9–4.0)% TL; skeletal interorbital space 0.8–1.0 times eye length, 8.7–11.7 times in horizontal preorbital length; posterior eye notches and suborbital grooves present. Spiracles moderately large, length 1.2–1.4% TL and 0.3–0.5 times eye length, left spiracle with 10–13 folds, right one with 10–13; spiracles strongly crescentic, oblique, directed posteroventrally from top to bottom, located just posterior to posterior eye notch, separated by a narrow but deep vertical groove along posterior margin of orbit, shorter than eye; upper edge below level of top of eye. Gill slits small, upright, weakly pleated, lateral on head, close to ventral surface, extending slightly onto ventral surface, subequal in length, sixth slit arches around pectoral-fin origin. Mouth large, strongly inferior, broadly arched, symphysis about level with posterior edge of eye, width 4.2–4.9% TL and 1.4–1.7 times in head width; upper labial furrows absent, lower furrows short, 0.5–0.6% TL; corner of mouth partly concealed by lateral muscles of jaw (Fig 27). Teeth unicuspidate, in well-defined series, bases oval and flattened with short but pronounced, narrow median cusp near middle of jaw, no lateral cusps; cusps diminishing in height towards jaw angles, indistinct near jaw corners; about 4–5 series of functional teeth (Fig 29). Median cusp with labial face slightly convex and with both mesial and distal cutting edges weakly bent mesially and distally in occlusal view, respectively. The mesial and distal crown base parts somewhat curve apically. A pronounced and broad, irregularly shaped apron overlaps the junction of crown and root, building a notch at the junction with both mesial and distal crown base parts. Basal ornamentation, striae or reticulations absent, sharp folds present in upper but absent in lower jaw teeth. The lingual face of the cusp is strongly convex, a well-developed uvula is present at the central crown base. The mesial/distal latero-lingual crown faces curve strongly towards the apex of the crown, forming a sharp notch with the uvula. The root is anaulacorhizid and slightly arched without lobation. The outer surface of the root shows up to four large basal foramina, which are mostly oval-shaped. The inner face of the root shows up to six well-developed foramina along the crown-root junction at each side of the uvula. The basal face of the root is flat, partly showing some outer foramina.

Nostrils small, widely separated, subcircular; nostril width 0.6–1.1% TL, 3.1–5.4 times in internarial width, 4.4–6.8 times in mouth width, 5.9–9.2 times in width of rostrum at nostrils; located distinctly forward of level of anterior margin of eye; distance from anterior nostrils to symphysis of upper jaw 1.2–1.4 times internarial space, distance from barbel origin to anterior nostrils 5.5–6.6)% TL. Anterior nasal flaps well developed, leaf-like, extended ventrally beyond nostrils; incurrent and excurrent apertures surrounded by pronounced marginal lobes; no nasoral or circumnarial grooves; no dermal lobes (Fig 27).

Lateral trunk dermal denticles densely set and slightly overlapping, with flat, tricuspidate crowns (Fig 28g and 28h). The lateral cusps are rather weakly pronounced but situated quite far anteriorly so that the median cusp is not much longer than the lateral cusps. The median ridge is strongly pronounced and reaches the tip of the median cusp. The lateral ridges are less pronounced and rarely reach the tips of the lateral cusps. The surface of the denticles is only weakly structured by reticulations close to base. Dermal denticles on rostrum fan-shaped, with an obtusely angled, weakly pronounced median cusp and no lateral cusps but with 6–7 strongly pronounced ridges. The surface of the rostral dermal denticles is only weakly structured by reticulations very close to base.

Pectoral fins large, anterior margin weakly convex, 10.7–12.2% TL and 1.3–1.6 times inner margin; apex narrowly rounded; posterior margin weakly concave, directed across horizontal axis at about origin of first dorsal fin; inner margin convex and strongly notched basally; free rear tip angular (Fig 26). Pelvic fins moderately large, anterior margin almost straight to slightly convex, 5.8–6.8% TL, 1.5–1.8 times in first dorsal-fin anterior margin, and 1.4–1.6 times in second dorsal-fin anterior margin; apex narrowly rounded; posterior margin concave; inner margin weakly convex and slightly notched basally; free rear tip broadly rounded; origin distinctly posterior to level free tip of first dorsal fin and well forward of level second dorsal fin origin (Fig 26).

First dorsal fin broad, semifalcate, anterior margin slightly convex; apex narrowly rounded; posterior margin slanting posteroventrally, slightly convex distally, strongly concave in basal three quarters; inner margin straight, free rear tip narrowly pointed; origin about opposite pectoral-fin free rear tips; insertion and free rear tip clearly anterior to level pelvic-fin origins (Fig 26). Second dorsal fin somewhat smaller than first but of similar shape, anterior margin weakly convex, apex very narrowly rounded; posterior margin weakly convex distally, strongly concave near basal three quarters; inner margin straight, free rear tip narrowly pointed; origin clearly behind level pelvic insertions; interdorsal space 1.4–1.6 times first dorsal-fin length, 1.6–1.9 times dorsal–caudal space; second dorsal-fin inner margin 0.8–1.1 times subterminal caudal-fin margin (Fig 26).

Caudal fin short, dorsal margin slightly convex, length 18.0–19.1% TL, 1.0–1.2 times in pelvic–caudal space and 3.7–5.4 times terminal caudal margin; lower post-ventral lobe absent, upper post-ventral margin slightly convex; terminal lobe well developed, caudal terminal margin slightly concave, apices angular (Fig 26). Ventral origin of caudal fin situated anteriorly due to low anterior fin ridge (Fig 26c).

Cranium: five anterior-most basiventral cartilages laterally expanded, with curved, dorsally reflected margins. Chondrocranium and cranial nerves highly modified to accomodate the elongated rostrum. Foramen magnum surrounded by crescent-shaped occipital condyles. Dorsal fenestra of the precerebral fossa egg-shaped, notched anteriorly and posteriorly (Fig 12c).

Skeletal meristics (from radiographs): monospondylous trunk vertebral centra: 53–56; diplospondylous precaudal centra: 49–51; total precaudal centra: 103–106; caudal centra: 50–55; total centra: 154–158.

Coloration. Fresh, prior to preservation (ERB 1105, ERB 1106, SAIAB 208021 and Uncatalogued; Fig 30): ground color medium to dark brown dorsally with a pronounced yellowish longitudinal stripe; uniform white ventrally; fins translucent dusky, upper post-ventral caudal-fin and pelvic-fin posterior margins narrowly edged white, weak white edges also present at posterior margins of pectoral and dorsal fins, as well as terminal caudal-fin margin; rostrum translucent dusky, dark edged and with two distinct longitudinal stripes dorsally; lateral rostral teeth dark-edged; ventrolateral keels white. Color in preservative (other material examined): coloration similar to fresh coloration but yellowish longitudinal dorsal stripe not detectable in all specimens, particularly after long-time storage in ethanol; ventral coloration uniform yellowish instead of white as usual, ventrolateral keels also yellowish; dark edging of rostrum and lateral rostral teeth still pronounced in most specimens but hardly detectable in the intact syntype which is more than 100 years old; longitudinal dorsal rostral stripes still conspicuous in all specimens including the intact syntype. Fresh photographs of one specimen caught off Mozambique and kindly provided by Oddgeir Berg Alvheim, as well as a photograph of one specimen from off South Africa, taken and kindly provided by Frederik Mollen, Elasmobranch Research Belgium, are shown in Fig 30.

#### Size

A large sawshark species reaching at least 1360 mm TL but possibly attaining 1700 mm TL [14,15]. Males are adolescent at 700 to 740 mm, mature at 830 mm and grow to at least 1120 mm TL, females are adolescent at around 950 to 1100 mm TL, are mature when over 1100 mm TL and attain at least 1360 mm TL [30]. The male specimen **ERB 1106** is subadult at 945 mm TL. The size at birth is about 350 mm TL, the litter size 5–7 pups, but up to 17 developing eggs recorded [18,30].

#### Distribution

Known from off South Africa and southern Mozambique in depths from 26 to 500 m (Fig 14). However, the maximum depth of 500 m is apparently based on erroneous data for the holotype of *Pliotrema kajae* sp. nov., indicating that the verified maximum depth of *P*. *warreni* was 430 m [30]. Nevertheless, *P*. *warreni* can be found in waters shallower and deeper than this based on specimens ERB 1105 (caught in 10–25 m depth) and one specimen from 915 m depth in the iSAM collection (SAM 33308, catch location 35.035°S 24.0217°E). Accordingly, the updated depth range for *P*. *warreni* is 10–915 m, albeit the species is usually found in 60–430 m depth [27,30]. *Pliotrema* sp. possibly occurs down to 1080 m depth [28] but it is impossible to assign this maximum depth to a certain species nor any verified specimen of *Pliotrema*. The northernmost verified records of *P*. *warreni* are from off southern Mozambique at about 22°S latitude.

#### Remarks

There are several morphometric differences between the small and large examined specimens of *Pliotrema warreni*, which might be of ontogenetic nature. However, the number of specimens and coverage of different sizes is too small to reliably detect ontogenetic differences. Nevertheless, like in *P*. *kajae*, the total number of large lateral rostral teeth and the number of large lateral rostral teeth posterior to barbels clearly differ between the smaller (405.9–456.4 mm TL) and larger (785–925 mm TL) specimens of *P*. *warreni* (total number 34–34/34–34 vs. 21–23/21–23; number posterior to barbels 18–19/18–18 vs. 6–6/ 5–7). Like in *P*. *kajae*, the large interstitial rostral teeth are serrated in large specimens of *P*. *warreni* (704 mm TL or larger), whereas all interstitial teeth are unserrated in specimens of 456.4 mm TL or smaller.

## Discussion

The three species of *Pliotrema* are apparently allopatric with the distribution of *P*. *kajae* being restricted to Madagascar and the Mascarene Ridge, *P*. *annae* known only from off Zanzibar, and *P*. *warreni* occurring off South Africa and southern Mozambique. They can further be differentiated based on numerous morphological, morphometric, and meristic characteristics.

Both new species differ from *Pliotrema warreni* in barbels that are situated about half way from rostral tip to mouth, with prebarbel length about equal to distance from barbel origin to symphysis of upper jaw (1.0–1.1 times in *P*. *kajae* and 1.0 times in *P*. *annae*) vs. barbels about two thirds way from rostral tip to mouth, with prebarbel length about twice, i.e. 1.7–2.1 times, distance from barbel origin to symphysis of upper jaw in *P*. *warreni*, prebarbel length 49.4–52.9% of preoral length in *P*. *kajae* and 50.7–51.1% in *P*. *annae* vs. 60.2–68.0% in *P*. *warreni*, preoral length 1.9–2.0 and 2.0 vs. 1.5–1.7 times prebarbel length, prenarial length 1.5–1.7 and 1.6–1.7 vs. 1.3–1.4 times prebarbel length.

All three species differ in the following characters: barbel origin to anterior nostrils 8.5–10.5% TL in *P*. *kajae* vs. 7.9–8.3% TL in *P*. *annae* vs. 5.5–6.6% TL in *P*. *warreni*, sharp folds present in both upper and lower jaw teeth vs. folds absent in upper and lower jaw teeth vs. folds present in upper but absent in lower jaw teeth, a rostrum that is clearly constricted between barbel origin and nostrils vs. slightly constricted vs. not constricted, and the shape of the dorsal fenestra of the precerebral fossa (teardrop-shaped, with posterior notch vs. spindle-shaped, elongate and long, notched anteriorly and posteriorly vs. egg-shaped, notched anteriorly and posteriorly; Fig 12). Furthermore, the three species differ in the anterior vent–caudal tip length (38.7–40.4% TL in *P*. *kajae* vs. 43.3–43.4% TL in *P*. *annae* vs. 40.4–41.7% TL in *P*. *warreni*), lower labial furrow length (0.4–0.5% TL vs. 0.3% TL vs. 0.5–0.6% TL), prebarbel length (14.8–16.2% TL vs. 12.6–12.7% TL vs. 16.5–20.2% TL), barbel origin to symphysis upper jaw (13.7–15.0% TL vs. 12.1–12.3% TL vs. 9.2–10.9% TL), barbel origin to anterior nostrils (8.5–10.5% TL vs. 7.9–8.3% TL vs. 5.5–6.6% TL), snout–anterior vent length / anterior vent–caudal tip length (1.5–1.6 vs. 1.3 vs. 1.4–1.5), the number of large rostral teeth anterior to barbels (12–14 vs. 10–11 vs. 14–18), and the coloration (pale to light brown dorsally with two yellowish stripes, uniform white ventrally, posterior fin margins with narrow white edges vs. uniform medium to dark brown dorsally without longitudinal stripes, white ventrally but with few indistinct dark blotches on belly, posterior fin margins conspicuously white-edged vs. medium to dark brown dorsally with a pronounced yellowish longitudinal stripe; uniform white ventrally).

*Pliotrema kajae* differs from *P*. *annae* also in a shorter pectoral–pelvic space (13.5–18.5% TL vs. 19.0–19.9% TL), caudal peduncle height 2.8–4.0 vs. 4.5–4.9 times in dorsal–caudal space, preorbital length 2.7–3.6 vs. 2.1 times first dorsal-fin length, preoral length 2.0–2.7 vs. 1.5–1.7 times interdorsal space, a broader mouth (width 4.4–5.4% TL vs. 4.1–4.3% TL), and more spiracle folds (12–15 vs. 10–11).

Additionally, *Pliotrema annae* differs from both congeners in the short snout (pre-D1-length 43.3–43,7% TL in *P*. *annae* vs. 47.2–50.6% TL in *P*. *kajae* vs. 45.4–49.0% TL in *P*. *warreni*, head length 34.2–34.5% TL vs. 38.3–40.4% TL vs. 35.5–40.1% TL, prebranchial length 29.6–30.5% TL vs. 34.2–36.6% TL vs. 32.1–35.8% TL, prespiracular length 24.7–25.6% TL vs. 29.0–31.9% TL vs. 27.2–30.9% TL, preorbital length 21.7–22.0% TL vs. 25.7–27.3% TL vs. 24.1–26.9% TL, prepectoral length 33.5–33.6% TL vs. 38.2–40.5% TL vs. 35.4–39.1% TL, prepelvic length 54.2–55.3% TL vs. 56.0–59.8% TL vs. 56.2–58.1% TL, prenarial length 20.5–21.1% TL vs. 24.1–26.3% TL vs. 22.6–25.5% TL, preoral length 24.6–25.1% TL vs. 28.6–31.3% TL vs. 26.7–30.2% TL), smaller eyes (eye length 2.7–2.8% TL vs. 2.8–5.2% TL vs. 2.9–4.0% TL), slightly larger pectoral fins (anterior margin length 12.7–13.4% TL vs. 10.3–12.2% TL vs. 10.7–12.2% TL), a larger first dorsal fin (total length 10.5–10.7% TL vs. 7.2–9.8% TL vs. 9.0–10.3% TL, anterior margin length 11.2–11.5% TL vs. 9.0–11.4% TL vs. 10.1–10.7% TL, base length 7.4–7.9% TL vs. 4.9–6.9% TL vs. 6.0–7.1% TL), a larger second dorsal fin (total length 8.9–9.3% TL vs. 7.6–8.6% TL vs. 5.4–8.8% TL, base length 6.3–6.4% TL vs. 5.1–6.3% TL vs. 5.4–6.0% TL, vertical height 6.8–6.9% TL vs. 4.5–6.2% TL vs. 5.2–6.4% TL), a larger pelvic fin (anterior margin length 7.0–7.1% TL vs. 5.3–6.7% TL, height 5.4–5.6% TL vs. 4.1–4.8% TL vs. 3.9–5.3% TL), smaller anterior nasal flaps (length 0.5–0.6% TL vs. 0.7–1.6% TL vs. 0.7–0.9% TL), shorter barbels (length 5.6% TL vs. 6.6–18.1% TL vs. 5.6–9.6% TL), a shorter interspiracular space (3.9% TL vs. 4.1–5.7% TL vs. 4.0–4.5% TL), a narrower rostrum (width at anterior nostrils 5.1% TL vs. 5.3–7.5% TL vs. 5.3–6.7% TL, width at origin of barbels 3.3–3.4% TL vs. 3.6–5.1% TL vs. 3.7–5.1% TL), as well as fewer total large lateral rostral teeth (16–17 vs. 21–31 vs. 21–34), ventral rostral spines anterior to nostrils (15 vs. 19–24 vs. 16–27), and tooth rows in upper (35–37 vs. 38–43 vs. 37–44) and lower (32–34 vs. 35–37 vs. 33–41) jaws.

**Fig 31:**
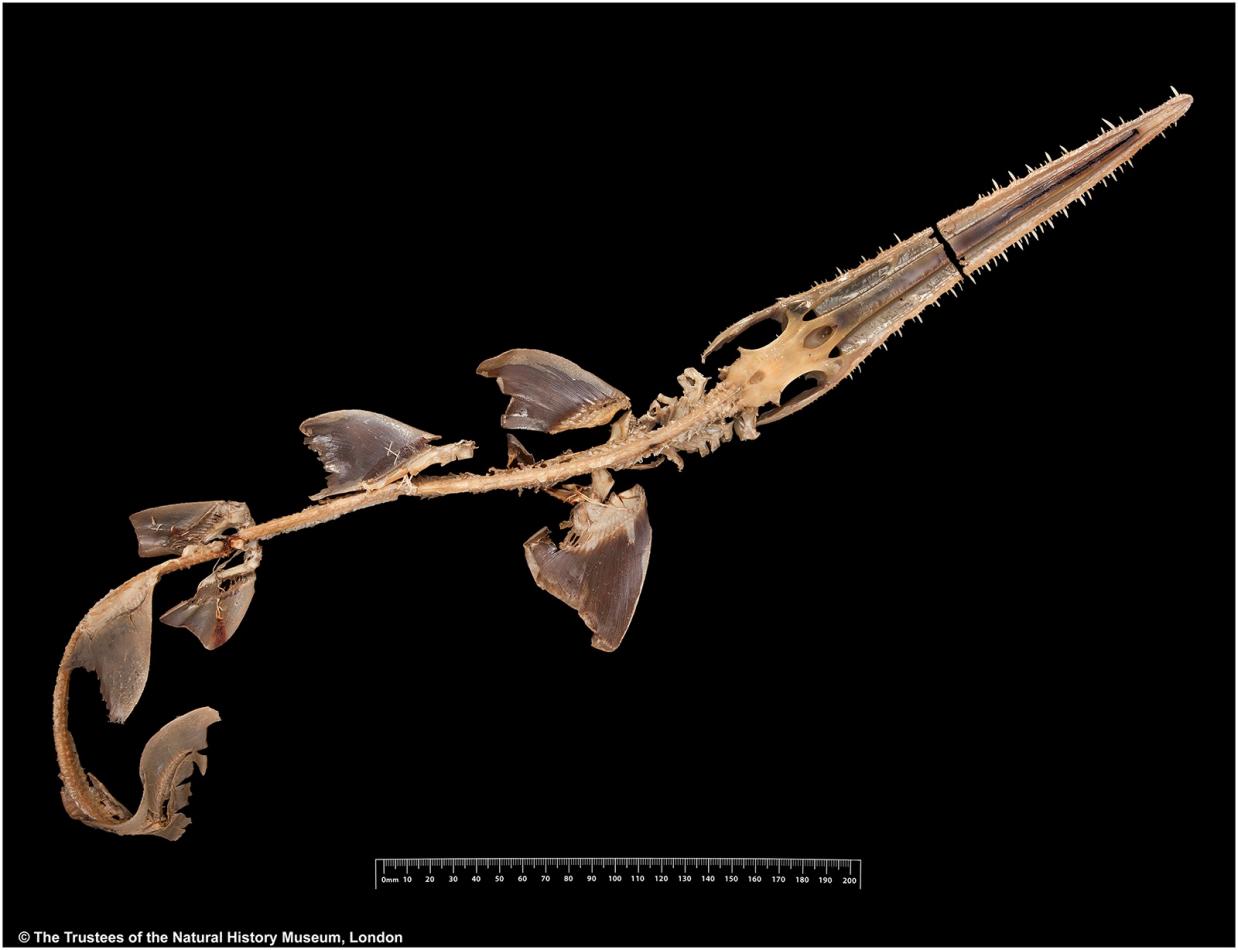
*Pliotrema warreni*, syntype, BMNH 1899.2.10.4, heavily dissected female, ~704 mm TL, in dorsal view. The photograph was taken and kindly provided by Kevin Webb.

The *Pliotrema warreni* syntype BMNH 1905.6.8.9 (Figs 26 and 27) was used as reference for characterizing the true *P*. *warreni*. The heavily dissected *P*. *warreni* syntype BMNH 1899.2.10.4 (Fig 31) could be identified as the same species despite its condition (only skeletal parts and remains of flesh and fins still exist), based on the measurements of horizontal prebarbel length (138 mm) and an estimation for the distance barbel origin to the symphysis of the (missing) upper jaw (~74 mm), resulting in a ratio of 1.9. This value is in line with *P*. *warreni* and clearly different from *P*. *kajae* and *P*. *annae*. The identification of the heavily dissected syntype is further evidenced by its catch location (False Bay, Cape of Good Hope), where both new species apparently do not occur. The identity of *Pliotrema* specimens occurring off Mozambique and, thereby, the possible presence of one of the new species in this area was investigated by examination of specimens from the DMM, iSAM, and USNM collections. As all specimens examined were clearly identified as *P*. *warreni* based, amongst other things, on the rostral morphology, it is assumed that *P*. *kajae* and *P*. *annae* do not occur off Mozambique and have a distribution allopatric to that of *P*. *warreni*. Nevertheless, the occurrence of *P*. *annae* off Zanzibar, close to the African mainland, indicates that the species may be distributed along the eastern African coast and overlap with *P*. *warreni* appears possible. It remains unclear if *Pliotrema* indeed occurs off Kenya and/or Somalia at all, as described for *P*. *warreni* in Gubanov [28]. Due to the proximity to Zanzibar it is conceivable that *Pliotrema* (i.e. *P*. *annae*) might be found in these areas but nevertheless would likely be very rare. *Pliotrema annae* appears to be rare also off Zanzibar and *P*. *kajae* also appears to be rare within its distribution area. The great catch depth of 915 m of one single specimen of *P*. *warreni* in the iSAM collection (and the possible maximum depth of 1080 m from Gubanov [28]) indicates that *Pliotrema* possibly enters depths beyond most fishing operations but such records appear to be very rare and both new species are likely affected by fishing operations in most of their bathymetric ranges. This assumption combined with the limited range and apparent rarity of both new species raises concerns that they are vulnerable to overfishing and might be in continuing decline, as has been previously suggested for *P*. *warreni* [2]. This could be particularly alarming for *P*. *annae* due to its very small known range, rarity and occurrence in shallow waters (the species is only known from depths of 20 to 35 m).

## Key to the valid species of *Pliotrema*

**1a**. Five pairs of gill openings, large lateral rostral teeth smooth ……………………………………………………………………………‥……. *Pristiophorus*

**1b**. Six pairs of gill openings, large lateral rostral teeth serrated (***Pliotrema***) …….………………‥………………………………………………………………………‥ **2**.

**2a**. Barbels about two thirds way from rostral tip to mouth, with prebarbel length about twice distance from barbel origin to symphysis of upper jaw; rostrum not constricted between barbel origin and nostrils; a pronounced yellowish longitudinal stripe on dorsal surface …………………………………… *Pliotrema warreni* [South Africa, southern Mozambique].

**2b**. Barbels about half way from rostral tip to mouth, with prebarbel length about equal to distance from barbel origin to symphysis of upper jaw; rostrum constricted between barbel origin and nostrils; dorsal surface without or with two longitudinal stripes ………………………………………‥ **3**.

**3a**. Snout long, head length 38.3–40.4% TL, preorbital length 25.7–27.3% TL, preoral length 28.6–31.3% TL, prebarbel length 14.8–16.2% TL, barbel origin to symphysis upper jaw 13.7–15.0% TL; rostrum clearly constricted between barbel origin and nostrils; 21–31 large lateral rostral teeth; 38–43 upper jaw tooth rows, jaw teeth with sharp basal folds; pale to light brown dorsally with two yellowish stripes, uniform white ventrally, posterior fin margins with narrow white edges ………… ***Pliotrema kajae* sp. nov**. [Madagascar, Mascarene Ridge].

**3b**. Snout short, head length 34.2–34.5% TL, preorbital length 21.7–22.0% TL, preoral length 24.6–25.1% TL, prebarbel length 12.6–12.7% TL, barbel origin to symphysis upper jaw 12.1–12.3% TL; rostrum only slightly constricted between barbel origin and nostrils; 16–17 large lateral rostral teeth; 35–37 upper jaw tooth rows, jaw teeth without basal folds; uniform medium to dark brown dorsally without longitudinal stripes, white ventrally but with few indistinct dark blotches on belly, posterior fin margins conspicuously white-edged ………………………………………………………‥… ***Pliotrema annae* sp. nov**. [Zanzibar].
